# Meso–macroporous hydrogel for direct litre-scale isolation of extracellular vesicles

**DOI:** 10.1038/s41565-025-02011-1

**Published:** 2025-09-24

**Authors:** Junbeom Kim, Minjin Kang, Geonhee Han, Sujin Hyung, Mina Kim, Minjeong Jang, Han Kyul Lee, Yunhee Seo, Ki Cheol Gil, Changheon Kim, Sojin Song, Seonghyeok Jeong, Seongchan Kim, Min Soo Kim, Ji Sung Shim, Sung Gu Kang, Young Chan Lee, Seok Chung, Il-Joo Cho, Tae Soup Shim, Kwang Hoon Song, Jouha Min, Hyejeong Seong, Kyungeun Lee, Jeeyun Lee, Cheolju Lee, Hong Nam Kim, Hyojin Lee, Sun Hwa Kim, Ji Yoon Kang, Ki Wan Bong, Nakwon Choi

**Affiliations:** 1 Brain Science Institute, Korea Institute of Science and Technology (KIST), Seoul, Korea; 2 Department of Chemical and Biological Engineering, Korea University, Seoul, Korea; 3 Department of Chemical Engineering, University of Michigan, Ann Arbor, MI USA; 4 Institute of Human Genetics, College of Medicine, Korea University, Seoul, Korea; 5 Medicinal Materials Research Center, Biomedical Research Division, Korea Institute of Science and Technology (KIST), Seoul, Korea; 6 Precision Medicine Research Institute, Samsung Medical Center, Seoul, Korea; 7 Division of Hematology-Oncology, Department of Medicine, Samsung Medical Center, Sungkyunkwan University School of Medicine, Seoul, Korea; 8 KU-KIST Graduate School of Converging Science and Technology, Korea University, Seoul, Korea; 9 Division of Radiation Biomedical Research, Korea Institute of Radiological & Medical Sciences, Seoul, Korea; 10 Chemical & Biological Integrative Research Center, Biomedical Research Division, Korea Institute of Science and Technology (KIST), Seoul, Korea; 11 KHU-KIST Department of Converging Science and Technology, Kyung Hee University, Seoul, Korea; 12 Advanced Analysis and Data Center, Research Resources Division, Korea Institute of Science and Technology (KIST), Seoul, Korea; 13 College of Pharmacy and Research Institute of Pharmaceutical Sciences, Gyeongsang National University, Jinju, Korea; 14 Division of Bio-Medical Science and Technology, KIST School, University of Science and Technology (UST), Seoul, Korea; 15 Department of Urology, College of Medicine, Korea University, Seoul, Korea; 16 Department of Otolaryngology-Head and Neck Surgery, Kyung Hee University College of Medicine, Kyung Hee University Hospital at Gangdong, Seoul, Korea; 17 School of Mechanical Engineering, Korea University, Seoul, Korea; 18 Department of Biomedical Sciences, College of Medicine, Korea University, Seoul, Korea; 19 Department of Convergence Medicine, College of Medicine, Korea University, Seoul, Korea; 20 Department of Anatomy, College of Medicine, Korea University, Seoul, Korea; 21 Department of Chemical Engineering, Ajou University, Suwon, Korea; 22 Department of Energy Systems Research, Ajou University, Suwon, Korea; 23 Department of Nano-Bioengineering, Incheon National University, Incheon, Korea; 24 Department of Biomedical Engineering, University of Michigan, Ann Arbor, MI USA; 25 Department of Macromolecular Science and Engineering, University of Michigan, Ann Arbor, MI USA; 26 Rogel Cancer Center, University of Michigan, Ann Arbor, MI USA; 27 Weil Institute for Critical Care Research and Innovation, University of Michigan, Ann Arbor, MI USA; 28 Biomaterials Research Center, Biomedical Research Division, Korea Institute of Science and Technology (KIST), Seoul, Korea; 29 Research Institute for Convergence Biomedical Science, College of Medicine, Korea University, Seoul, Korea; 30 Bio-Medical Research Center, Korea University Guro Hospital, Seoul, Korea

**Keywords:** Nanopores, Biomaterials, Synthesis and processing, Biomedical engineering, Characterization and analytical techniques

## Abstract

Extracellular vesicles are cell-originated lipid bilayer membrane vesicles that play vital roles in cell-to-cell communications. While extracellular vesicles hold substantial biomedical potential, conventional methodologies for isolating extracellular vesicles require elaborate preprocessing and, therefore, remain labour intensive and limited by throughput. To overcome these challenges, we present a facile fabrication route for generating a meso–macroporous hydrogel matrix with pores of ~400 nm for customizable extracellular vesicle isolation. By combining surface charge-selective capture of extracellular vesicles within the hydrogel matrix and their recovery by high ionic strength, we report direct extracellular vesicle isolation with a throughput range from microlitre to litre scales, without preprocessing, for various biofluids, including whole blood, plasma, ascites, saliva, urine, bovine milk and cell culture media. Furthermore, we demonstrate that the meso–macroporous hydrogel also serves as a solid-phase matrix for preserving extracellular vesicles for on-demand downstream analyses, making it applicable for therapeutics, cosmeceuticals and disease diagnostics.

## Abstract (web summary)

A hydrogel matrix fabricated by cryo-photocrosslinking enables the direct, scalable isolation of extracellular vesicles from diverse biofluids without preprocessing, allowing for in-gel preservation for long-term storage and biomedical applications.

## Main

Extracellular vesicles (EVs) are lipid bilayer membrane vesicles heterogeneously ranging from 30 to 1,000 nm that are secreted by cells^1,2^ in body fluids and the systemic circulation^3,4^. EVs play a role in cell-to-cell communication^5,6^, as their contents reflect compositions of the cells of origin, including RNAs, DNAs, lipids and proteins^7^. Therefore, EVs are considered carriers of biomarkers for various diseases, including cancer^8–10^ and neurodegenerative diseases^11^. In biomedicine, the applications of EVs have expanded into therapeutics as delivery cargos, such as mesenchymal-stem-cell-derived therapeutic components for injury mitigation^12^ and drugs that cross biological barriers (for example, the blood–brain barrier) to enhance efficacy^13^.

While the relevance of EVs has become prominent in various academic and industrial fields in a cross-disciplinary manner, the need for a readily accessible yet efficient and customizable method to isolate EVs has also grown, alongside an unmet need for scale-up^14^. Despite numerous reported methodologies for isolating EVs^15–17^, no single approach satisfies all four aspects of efficiency, scalability, accessibility and customizability simultaneously, hindering the use of EVs in further investigations, including clinical applicability. Ultracentrifugation (UC) and density gradient UC, which are traditionally considered the gold standard for EV isolation, primarily require expensive equipment and are accompanied by time-consuming and complicated processes. Although size exclusion chromatography has shown its potential utility as a UC-free alternative^18^, it is not possible to concentrate EV isolates^19^ with this method because of its passive sieving nature (Supplementary Discussion). Moreover, size exclusion chromatography is only optimal in a limited capacity range and requires relatively high costs. While emerging technologies, such as asymmetrical flow field-flow fractionation^20^, tangential flow filtration^21^, electrochemical fluidic stimulation^22^ and double-coupled harmonic oscillation (that is, EXODUS)^23^ could also serve as appealing alternatives (Supplementary Discussion), it is notable that all conventional and contemporary EV isolations require preprocessing of samples, such as serial centrifugation and microfiltration. We aimed to address the four influential aspects mentioned above with a unique combination of a hydrogel possessing pores permeable to EVs and active-capture-based isolation within the hydrogel matrix. Strikingly, the definition of macroporous has been vaguely broad, from 50 nm to 1 µm, based on the classification by size^24,25^. This study presents a meso–macroporous hydrogel matrix with pores of ~400 nm for facile and scalable isolation of EVs, up to litres, without preprocessing for various biofluids, including whole blood, plasma, ascites, saliva (oral swirl), urine, bovine milk and cell culture media, facilitating a rapid access to downstream analyses. Moreover, the customizability extends to preserving EVs within the hydrogel for long-term storage and enriching them on demand upon recovery at the end user’s convenience.

## Meso–macroporous hydrogel particles for direct EV isolation

**Fig. 1:**
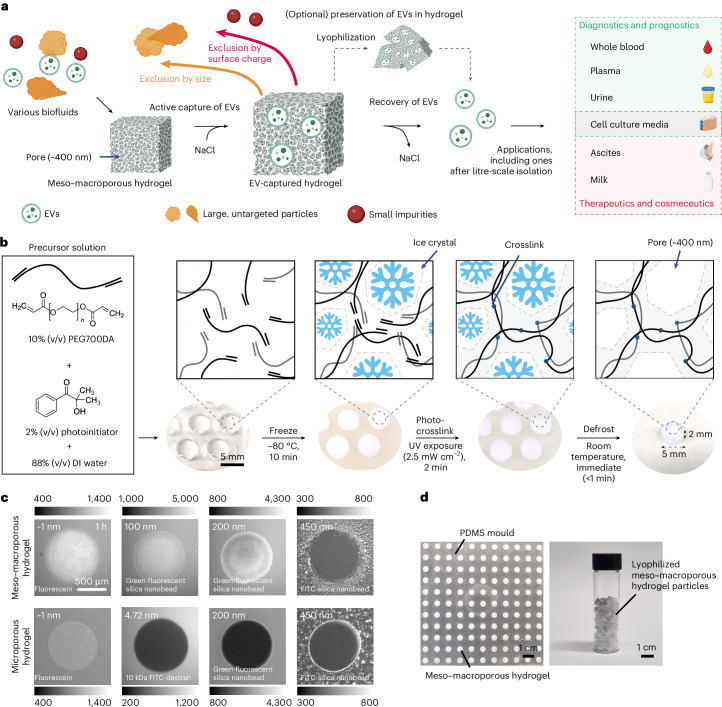
Overview of hydrogel-based direct EV isolation and fabrication and porosity characterization of meso–macroporous hydrogel particles. **a**, Schematic overview depicting direct EV isolation with meso–macroporous hydrogel from various biofluids and its versatile applications in diagnostics, prognostics, therapeutics and cosmeceutics, including litre-scale isolation and preservation of EVs within the hydrogel. **b**, Schematic diagram displaying cryo-photocrosslinking process to fabricate meso–macroporous PEG700DA particles. DI, deionized. **c**, Fluorescence images of meso–macroporous (top) and microporous (bottom) particles during one-dimensional (1D) radial diffusion of various fluorescent indicators at 1 h: fluorescein (~1 nm), 10 kDa dextran labelled with fluorescein isothiocyanate (FITC; 4.72 nm), green-fluorescent silica nanobeads (100 and 200 nm) and FITC-silica nanobeads (450 nm). Intensity scale bars are identical to each indicator. **d**, Photographs showing an 11 × 11 array snapshot of meso–macroporous hydrogel particles (left) and lyophilized meso–macroporous particles in a glass vial for storage (right). **a** created with BioRender.com.

We used polyethylene glycol diacrylate (PEGDA) hydrogel particles as a convenient, gel-phase medium that actively captures EVs (Fig. 1a). To fabricate the meso–macroporous hydrogel particles, we cryo-photocrosslinked an aqueous PEGDA precursor solution (Fig. 1b). We chose PEGDA with a molecular weight (*M*_n_) of 700 Da (PEG700DA) due to its having the highest *M*_n_ in the liquid phase at room temperature, its commercial availability and its affordability (Supplementary Table 1). To verify the meso–macroporosity, we diffused fluorescent indicators of various sizes, ranging from ~1 nm to 450 nm, into cryo-photocrosslinked and microporous hydrogel posts. The indicators up to 200 nm diffused well within 1 h, whereas the 450 nm nanobeads were impermeable. By contrast, a 4.72 nm indicator and the larger ones remained impenetrable to a microporous hydrogel photocrosslinked without pre-freezing (Fig. 1c). To demonstrate the feasibility of the scalable production of these meso–macroporous hydrogel particles, we simultaneously fabricated 121 particles per batch using a polydimethylsiloxane (PDMS) well array under wide-field exposure to ultraviolet (UV; 365 nm; Fig. 1d).

## Underlying principles of hydrogel-based EV isolation

**Fig. 2:**
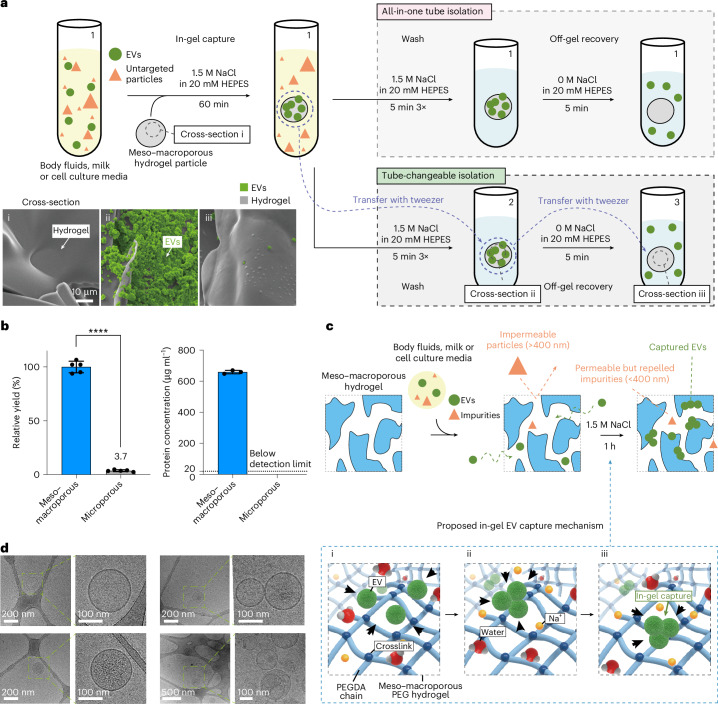
Procedure and underlying principles of meso–macroporous-hydrogel-based direct EV isolation. **a**, Schematic diagram displaying EV isolation procedure with meso–macroporous hydrogel particles: all-in-one tube isolation (pink-shaded box) or tube-changeable isolation (green-shaded box). Labels 1, 2 and 3 represent successive transfers of the sample into fresh tubes at each step of the procedure. Each SEM image represents a cross-section of meso–macroporous particles before EV isolation (i), after in-gel capture of EVs (ii) or after off-gel recovery of EVs (iii). **b**, Yield and protein concentration of EVs with meso–macroporous (blue) and microporous (grey) hydrogel particles (technical replicates, *n* = 5 for yield and 3 for protein concentration). Statistical significance, ****P < 0.0001. **c**, Schematic illustrations depicting the underlying principles of active in-gel capture of EVs mediated by meso–macropores of hydrogel and a salt (NaCl), rendering the additional exclusion of permeable but repelled impurities (<400 nm). Dashed boxes (i–iii) illustrate dynamic snapshots of the proposed in-gel EV capture mechanism. **d**, Cryogenic TEM images of EVs isolated with meso–macroporous hydrogel, showing lipid bilayer structure. Zoomed-in views are indicated by the green dashed boxes. Source data

Simply applying the meso–macroporous hydrogel particles and salt into an EV-containing sample of interest allowed the isolation of EVs with neither preprocessing nor equipment in three steps: (1) in-gel capture, (2) wash and (3) off-gel recovery (Fig. 2a). All the capture, wash and recovery buffers maintained constant pH at 7.4 with 20 mM *N*-2-hydroxyethylpiperazine-*N*-2-ethane sulfonic acid (HEPES). Depending on applications, end users can choose either an all-in-one tube isolation (pink dotted box at top of Fig. 2a) or a tube-changeable isolation (green dotted box at bottom of Fig. 2a); both approaches showed indistinguishable yield (Supplementary Fig. 1). Also, we intentionally designed the particles of 5 mm in diameter for those without technical expertise to be able to transfer the particles with tweezers into new tubes; in other words, extra centrifugation to harvest them is unnecessary. Scanning electron microscopy (SEM), transmission electron microscopy (TEM) and correlative light and electron microscopy (CLEM) of cross-sections (Fig. 2a(i)–(iii) and Supplementary Figs. 2 and 3) revealed that the hydrogel initially with no EVs contained human plasma EVs after the in-gel capture and multiple washes under the high salt condition and that the removal of the salt led to the successful recovery of EVs. Evidently, the meso–macroporosity provided a foundation for enabling our hydrogel-based EV isolation, resulting in an approximately 28-fold increase in yield compared with the conventional microporous hydrogel (Fig. 2b). Similarly, the protein concentration of isolates with the meso–macroporous hydrogel was measurable, whereas that with the microporous hydrogel was below the detection limit.

**Extended Data Fig. 1:**
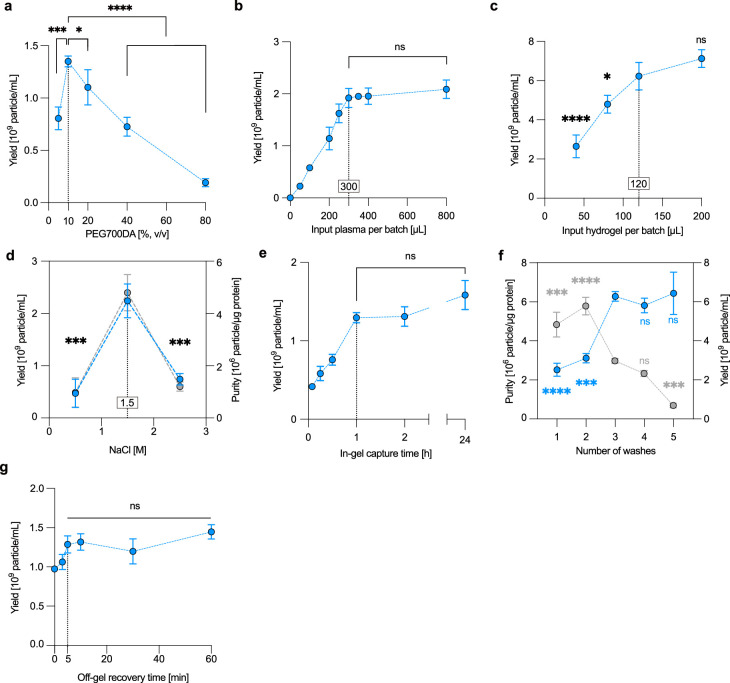
Optimization of meso–macroporous hydrogel-based direct EV isolation. **a**, Yield of human plasma EVs by varying the volumetric composition of PEG700DA in precursor solutions. Statistical significance, ***P = 0.0002, *P = 0.0443 and ****P < 0.0001. **b**, Yield of EVs by varying input volume per batch while fixing the volume of the meso–macroporous hydrogel as 40 µL (1 particle). Statistical significance to 300 µL, NS *P* > 0.9999 (350 µL), *P* = 0.9998 (400 µL) and *P* = 0.3997 (800 µL). **c**, Yield of EVs by varying the volume of meso–macroporous hydrogel (that is, the number of hydrogel particles; 40 µL each) per batch while fixing the input plasma volume as 800 µL. Statistical significance to 120 µL (3 particles), ****P < 0.0001, *P = 0.0343 and NS *P* = 0.2161. **d**, Yield (blue) and purity (grey) of EVs depending on ionic strength (that is, NaCl concentration) during the in-gel capture. Statistical significance to 1.5 M, ***P = 0.0003 (0.5 M) and 0.0006 (2.5 M) for yield; ***P = 0.0002 (0.5 M) and 0.0003 (2.5 M) for purity. **e**, Yield of EVs depending on in-gel capture time. Statistical significance to 1 h, NS *P* = 0.9998 (2 h) and 0.0626 (24 h). **f**, Purity (blue) and yield (grey) of EVs depending on the number of washes. Statistical significance to 3 washes, ****P < 0.0001 (1 wash), ***P = 0.0001 (2 washes) and NS *P* = 0.6999 (4 washes) and 0.9896 (5 washes) for purity; ***P = 0.0006 (1 wash), ****P < 0.0001 (2 washes), NS *P* = 0.2559 (4 washes) and ***P = 0.0001 (5 washes) for yield. **g**, Yield of EVs depending on off-gel recovery time. Statistical significance to 5 min, NS *P* = 0.9961 (10 min), 0.7515 (30 min) and 0.2854 (60 min). For all these plots, error bars indicate mean ± s.d. (technical replicates, *n* = 3 for **a**, **c**, **e**, **f** and **g** and 5 for **b** and **d**). Source data

To establish this framework of the meso–macroporous hydrogel-based EV isolation, we experimentally optimized six critical parameters with human plasma (Extended Data Fig. 1, Supplementary Fig. 4 and Supplementary Results): (1) the composition of the PEGDA precursor solution (that is, 10% (v/v) PEG700DA), (2) the volumetric processing throughput (that is, 300 μl plasma per 40 μl hydrogel), (3) ionic strength during the in-gel capture and washes (that is, 1.5 M NaCl), (4) in-gel capture time (that is, 1 h), (5) the number of washes (that is, three times) and (6) off-gel recovery time (that is, 5 min).

During the in-gel capture, our meso–macroporous hydrogel allowed permeation of nanoparticles, including EVs, under ~400 nm and excluded impermeable particles. Meanwhile, PEG chains in the gel phase served as a three-dimensional (3D) mesh, actively capturing only EVs with a specific surface charge^26^, aggregated by the surrounding high ionic strength (Fig. 2c). More specifically, regarding the underlying principle, the transport of EVs into the meso–macropores would be equivalent to the local encompassment of EVs by gel-phase PEG chains (Fig. 2c(i)), which resembles a situation in which EVs engage each other more closely by the steric shielding effect of mobile liquid-phase PEG chains. Simultaneously, a high concentration of Na^+^ diminishes the repulsive interaction between the EVs by replacing surrounding water molecules that exist near hydrophilic PEG, making inter-EV attraction more favourable^26^ (Fig. 2c(ii)). Consequently, the combined effect of each component would induce aggregation and capture of EVs in the meso–macroporous hydrogel (Fig. 2c(iii)).

Because permeable impurities such as cholesterol, including very low-density lipoprotein (VLDL) and low-density lipoprotein (LDL), possess different surface charges compared with EVs^27,28^, 1.5 M NaCl would exhibit nearly no effect on the aggregation of these small impurities; we experimentally confirmed repelling (V)LDL (apolipoprotein B (APOB) bands in Extended Data Fig. 3a,e). Furthermore, we performed Western blotting to verify whether isolates from various biofluids might contain calnexin (CNX), APOB, apolipoprotein A1 (APOA1), golgin subfamily A member 2 (GOLGA2; 130 kDa *cis*-Golgi matrix protein 1) and casein alpha S1 (CSN1S1), which showed that non-EV impurities were either undetectable or markedly diminished compared with positive controls (Extended Data Fig. 3e).

Morphological characteristics of off-gel recovered nanoparticles, as observed by cryogenic TEM, displayed a lipid bilayer structure (Fig. 2d), consistent with the morphology of EVs reported elsewhere. To further examine whether our methodology cleans EV-containing biofluids from contaminants, we performed negative-stain TEM on (1) various native biofluids, (2) off-gel recovered isolates and (3) remains (that is, residual EV isolation medium after in-gel capture before washes). Negative-stain TEM images revealed that isolated EVs were almost free of visually identifiable contaminants, such as lipoproteins^29^ and viruses^30^, while all the native biofluids and remains contained mostly large (>500 nm) aggregates (Extended Data Fig. 4). These data affirmed the effectiveness of our hydrogel-based EV isolation in filtering out undesired impurities.

In alignment with the minimal information for studies of extracellular vesicles (MISEV) 2024 guidelines^31^, we further performed in-depth characterizations of off-gel recovered EVs and compared them with established methods using various analytical techniques (Supplementary Results), including nanoparticle tracking analysis (Extended Data Fig. 3b,d), Bradford assay (Extended Data Fig. 3c), Western blot (Extended Data Fig. 3a,e), proteomics (Extended Data Fig. 5) and RNA sequencing (Extended Data Fig. 6). These results verified that the off-gel recovered nanoparticles were indeed EVs and that our approach exhibited competently higher or comparable isolation efficiency (Supplementary Figs. 5 and 6) and purity, with the similar protein composition indicating EV subpopulations similar to those from existing methodologies (Extended Data Fig. 5).

**Extended Data Fig. 2:**
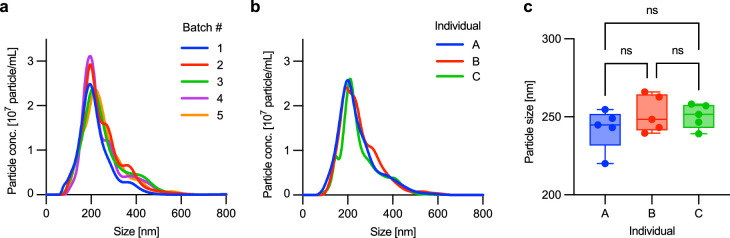
Reproducibility of meso–macroporous hydrogel-based isolation across batches and individuals. **a**,**b**, Size distributions of human plasma EVs isolated by hydrogel across five batches (**a**) and three individuals (**b**). **c**, Size of EVs isolated by different individuals. Statistical significance, NS *P* = 0.3908 (A-B), 0.5503 (A-C) and 0.9771 (B-C). Box plots display the interquartile range (box), with the horizontal line inside each box representing the median and the whiskers indicating the minimum and maximum values (technical replicates, *n* = 5). Source data

To assess the reproducibility when accounting for batch-to-batch and person-to-person variations, we prepared multiple batches of the hydrogel-based EV isolation, including some by one experimenter and some by different individuals who independently performed the process, from fabricating hydrogel particles to EV isolation. We found very similar profiles of the isolated EV populations across five batches (Extended Data Fig. 2a) and three different individuals (Extended Data Fig. 2b), including statistically indistinguishable size distributions (Extended Data Fig. 2c). Therefore, the established procedure exhibited robust reproducibility.

## Litre-scale EV isolation with meso–macroporous hydrogel

**Fig. 3:**
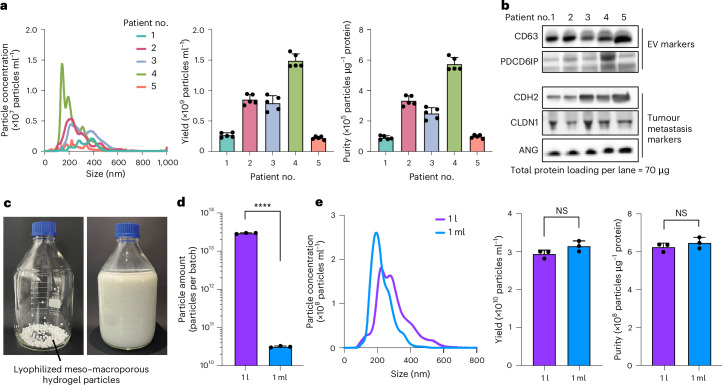
Litre-scale direct EV isolation with meso–macroporous hydrogel particles. **a**,**e**, Size distribution, yield and purity of gastric cancer patients’ ascites EVs (**a**) and bovine milk EVs (**e**) isolated by hydrogel. Statistical significance, not significant (NS) *P* = 0.1119 (yield in **e**), *P* = 0.3422 (purity in **e**). Error bars indicate mean ± s.d. (technical replicates, *n* = 5 for **a** and 3 for **e**; biological replicates in **a**, *n* = 5). **b**, Western blot images showing the expression of EV-positive (CD63 and PDCD6IP) and tumour metastasis (CDH2, CLDN1 and ANG) markers from the gastric cancer patients’ ascites EVs. **c**, Photographs showing lyophilized hydrogel particles (left) in a glass bottle and the bottle with 1 l of milk poured in (right). **d**, Amount of milk EVs isolated from 1 l (purple) and 1 ml (blue). Statistical significance, ****P < 0.0001. Error bars indicate mean ± s.d. (technical replicates, *n* = 3). Source data

By scaling up our hydrogel-based EV isolation with a proportionally increased number of hydrogel particles, we isolated EVs from 1 l of ascites fluid extracted from a gastric patient and from 1 l of bovine milk. Ascites EVs isolated from each of five advanced gastric cancer patients (Supplementary Table 2) showed ordinary EV-like size profiles, yields and purities, considering patient-to-patient variations (Fig. 3a). Also, we verified that all the patients’ ascites EVs displayed EV-positive markers of CD63 and PDCD6IP (ALIX) proteins and tumour metastasis markers of *N*-cadherin (CDH2), claudin-1 (CLDN1) and angiogenin (ANG; Fig. 3b). When isolating EVs from the scaled-up batch with 1 l of milk (Fig. 3c), we acquired three orders of magnitude more, a considerable amount, than with 1 ml through the identical isolation process and time (Fig. 3d). The size distribution, yield and purity of milk EVs were almost the same regardless of the batch volume (Fig. 3e), which indicates consistent characteristics across varying sample volumes.

Our approach would foster EV-associated biomedical research moving forward towards the industrialization stage. To elaborate on the sustainability of our technology, we verified the reusability of our meso–macroporous particles. Notably, we found that the yield of EVs isolated with reused hydrogel particles was statistically indistinguishable (Extended Data Fig. 7). Therefore, the reusability of meso–macroporous hydrogel particles would enable more sustainable and cost-effective EV isolation, facilitating the scaling up of this approach for industrial applications.

## Meso–macroporous hydrogel as EV-preserving carrier

**Fig. 4:**
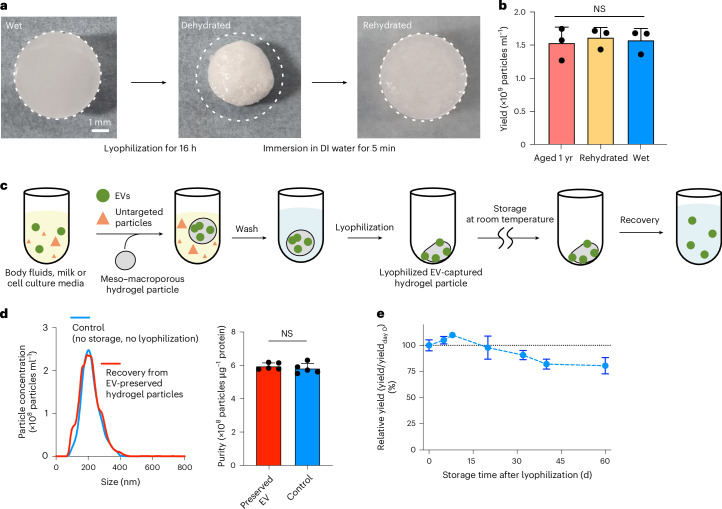
Meso–macroporous hydrogel as EV-preserving carrier. **a**, Photographs of meso–macroporous hydrogel particles in wet (left), dehydrated (middle) and rehydrated (right) states. White dashed circles indicate the particle size in the wet state before dehydration through lyophilization. **b**, Yield of EVs isolated with meso–macroporous particles in the aged state (red; 1 year equivalent acceleration), rehydrated-after-dehydration state (yellow) and wet state (blue). Statistical significance, NS *P* = 0.8578. **c**, Schematic diagram displaying the procedure to isolate EVs from lyophilized EV-captured meso–macroporous hydrogel particles after storage at room temperature. **d**, Size distribution and purity of EVs recovered immediately (control, blue) and from lyophilized EV-captured hydrogel particles after storage for 5 days (red). Statistical significance, NS *P* = 0.4445. **e**, Relative yield (the yield divided by the yield on day 0) of isolated EVs over storage time after lyophilization. Error bars indicate mean ± s.d. (technical replicates, *n* = 3 for **b** and 5 for **d** and **e**). Source data

Unlike macroporous hydrogels, which generally suffer from excessive swelling due to the irrevocability of mechanical strength after dehydration^32^, our meso–macroporous hydrogel particles sustained their structural integrity after cycles of dehydration and rehydration (Fig. 4a and Supplementary Fig. 7). We also tested their long-term stability by isolating EVs with hydrogel particles in three states: (1) aged 1 year through an accelerating condition at 94 °C for 3 days, (2) rehydrated after lyophilization and (3) immediately fabricated (wet). We considered that the elevated temperature provided an acceleration factor of 128 (2^7^) compared with storage at 24 °C (ref. ^33^). The result showed no statistically significant differences in the yield of plasma EVs (Fig. 4b), confirming the remarkable stability even under extreme conditions.

In connection with this long-term stability, our meso–macroporous hydrogel offers a practical solution as a solid-phase EV-preserving carrier, extending beyond the EV-isolating matrix and transcending the spatiotemporal limits of EV use. As a prime example of preserving EVs within the hydrogel, we lyophilized hydrogel particles after the in-gel capture and washes, and before the off-gel recovery (Fig. 4c). We found no difference in size distribution, yield or purity between EVs recovered immediately and those from lyophilized EV-captured hydrogel particles after storage at room temperature for several days (Fig. 4d). Long-term storage at room temperature for up to 60 days showed that the yield remained relatively stable for up to 32 days and gradually decreased by ~20% through day 60 (Fig. 4e). These results demonstrate better performance in long-term storage even without a cold chain; by contrast, Görgens et al.^34^ reported yields declining by ~80% when stored at –20 °C and ~40% at –80 °C at 8 weeks.

## Customizability and versatility of hydrogel-based isolation

**Fig. 5:**
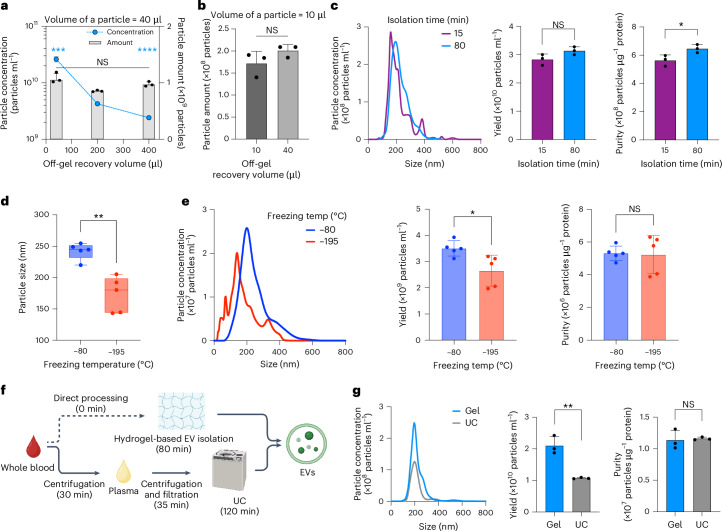
Customizability and versatility of hydrogel-based EV isolation. **a**, Yield (blue) and amount (grey) of EVs by varying off-gel recovery volume. Statistical significance to 200 µl, ***P = 0.0002 and ****P < 0.0001 for yield; NS *P* = 0.3949 (40 µl) and 0.2104 (400 µl) for amount. **b**,**c**, Amount of EVs isolated with 10 µl hydrogel particles by varying off-gel recovery volume (**b**), and size distribution, yield and purity by varying isolation time (**c**). Statistical significance, NS *P* = 0.1779 (**b**) and 0.0894 (yield in **c**) and *P = 0.0403 (purity in **c**). **d**, Size of EVs by varying freezing temperatures during cryo-photocrosslinking of hydrogel precursor. Statistical significance, **P = 0.0011. **e**, Size distribution, yield and purity of EVs isolated by hydrogel particles frozen at –80 °C (blue) and –195 °C (red) during cryo-photocrosslinking. Statistical significance, *P = 0.0204 (yield) and NS *P* = 0.8866 (purity). **f**, Comparative schematic diagrams depicting the procedure of EV isolation from human whole blood with timelines. **g**, Size distribution, yield and purity of whole blood EVs isolated by hydrogel (blue) and UC (grey). Statistical significance, **P = 0.0035 (yield) and NS *P* = 0.7986 (purity). Error bars indicate mean ± s.d. (technical replicates, *n* = 3 for **a**–**c** and **g** and 5 for **d** and **e**). **f** created with BioRender.com. Source data

Because we could precisely adjust the off-gel recovery volume while retaining the same amount of EVs, the hydrogel-particle-based isolation enabled custom enrichment of EVs on demand (Fig. 5a). This EV enrichment was also achievable by cryo-photocrosslinking smaller hydrogel particles (Supplementary Fig. 8), with which we reduced the recovery volume further to 10 μl without losing EVs (Fig. 5b). Moreover, these smaller particles manifested an additional capability, finishing EV isolation in 15 min; we observed a similar performance when applying the typical 80 min protocol (Fig. 5c). The slight decrease in purity (*P = 0.0403) caused by the 15 min isolation originated from the very short wash time (that is, <2 s) of the three washes, leading to less effective impurity removal. In other words, we immediately finished each wash without incubating for 5 min, prioritizing a faster isolation process over maximizing purity. When carrying out each wash for 5 min, we observed progressively improved purity, which became statistically indistinguishable from that of the 80 min isolation (Extended Data Fig. 8).

Furthermore, we explored the possibility of tuning the meso–macroporosity by controlling the freezing temperature to introduce a compelling insight for isolating EV subpopulations of different sizes. Accordingly, we could isolate a subpopulation of smaller EVs (Fig. 5d) with slightly reduced yield and retained purity (Fig. 5e), due to the more rapid formation of smaller ice crystals at a much lower temperature. This finding adds notable value, consistently originating from the customizability of our methodology.

The meso–macroporous hydrogel particles offer versatile applicability to any EV source of biofluids beyond human plasma, including whole blood, oral swirls and cell culture media. In particular, we emphasize our EV isolation from whole blood, in the presence of red blood cells, white blood cells and platelets. In other words, all existing methodologies obligatorily require the elimination of the blood cells and platelets, for instance, by serial centrifugations and filtration (Fig. 5f). We demonstrated an approximately two times higher yield and shorter isolation time from human whole blood while keeping purity nearly the same, compared with UC (Fig. 5g). We also found that off-gel recovered nanoparticles included a similar portion of small EVs (30–200 nm)^9^, compared with those isolated by UC (Supplementary Fig. 9). For mouse whole blood, yield and purity by the hydrogel particles were 3 and 1.2 times higher, respectively (Extended Data Fig. 9a).

Oral swirls and stem cell culture media have become increasingly of interest for their non-invasive diagnostic and therapeutic potential^12,35^, respectively. However, lower EV concentrations, by ~10–100-fold relative to other biofluids tested in this study, and the high viscosity characteristic of saliva pose additional challenges in isolating EVs using conventional techniques. Our approach was readily adaptable to overcoming these challenges, resulting in a comparable or higher yield and purity compared with UC for human saliva (Extended Data Fig. 9b) and human embryonic stem cell (hESC) culture media (Extended Data Fig. 9c). Moreover, the hydrogel-particle-based isolation yielded a higher proportion of small EVs, in contrast with UC, often leading to the aggregation of particles, conceivably attributed to undesirably high shear stress^36^.

## Downstream analyses for therapy and diagnosis

**Fig. 6:**
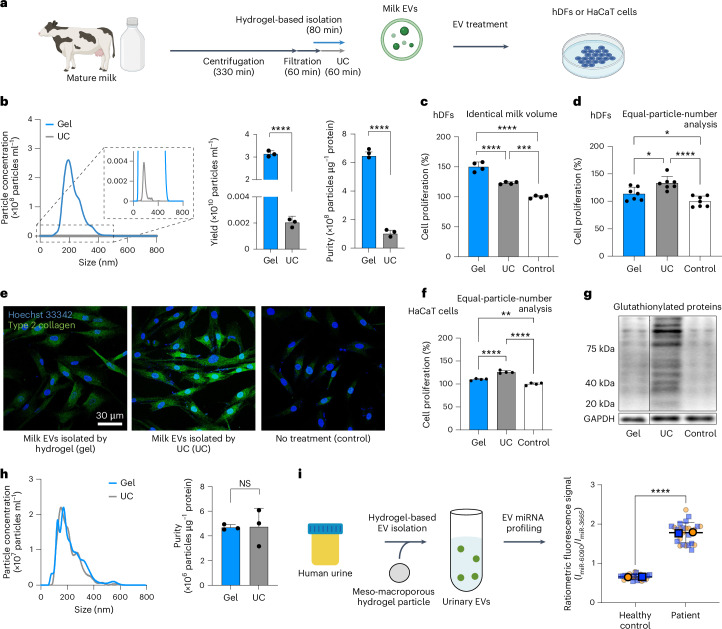
Downstream analyses for therapy and diagnosis. **a**, Comparative schematic diagram depicting the procedure of EV isolation from bovine milk with timelines. **b**, Size distribution, yield and purity of milk EVs isolated by hydrogel (blue) and UC (grey). Statistical significance, ****P < 0.0001. Error bars indicate mean ± s.d. (technical replicates, *n* = 3). **c**,**d**, Identical-milk-volume (**c**) and equal-particle-number (**d**) analyses for the proliferation of hDFs at 3 days in vitro (DIV 3) after treating milk EVs isolated by hydrogel (blue) and UC (grey) for 24 h, relative to proliferation without milk EVs (white). Statistical significance, *P = 0.0110 (gel–UC in **d**) and 0.0348 (gel–control in **d**), ***P = 0.0003 and ****P < 0.0001. Error bars indicate mean ± s.d. (technical replicates, *n* = 4 for **c** and 7 for **d**). **e**, Fluorescence images showing hDFs’ nuclei (blue) and expression of collagen II (green), depending on the treatment with milk EVs—isolated by hydrogel (left), isolated by UC (middle) and no treatment (right)—as part of an equal-particle-number analysis. **f**, Equal-particle-number analysis for the proliferation of HaCaT cells at DIV 3 after treating with milk EVs isolated by hydrogel (blue) and UC (grey) for 24 h, relative to proliferation without milk EVs (white). Statistical significance, **P = 0.0014 and ****P < 0.0001. Error bars indicate mean ± s.d. (technical replicates, *n* = 4). **g**, Western blot image showing glutathionylated proteins and GAPDH protein expression by HaCaT cells as part of an equal-particle-number analysis. **h**, Size distribution and purity of human urine EVs isolated by hydrogel (blue) and UC (grey). Statistical significance, NS *P* = 0.7522. Error bars indicate mean ± s.d. (technical replicates, *n* = 3). **i**, Schematic diagram of profiling urinary EV miRNAs as a diagnostic downstream analysis, and a SuperPlot displaying ratiometric fluorescence signals (intensity *I* of miR-6090 divided by *I* of miR-3665) from 12 healthy controls and 12 prostate cancer patients. Statistical significance, ****P < 0.0001. Error bars indicate mean ± s.d. (biological replicates pooled from 12 controls or patients, *n* = 2; technical replicates, the number of conventional hydrogel particles (Supplementary Fig. 10), *n* = 13). **a** created with BioRender.com. Source data

We applied the hydrogel-based EV isolation to two distinct downstream domains: (1) therapeutics and cosmeceutics and (2) diagnostics and prognostics. EVs in bovine milk have recently gained considerable attention due to their therapeutic potential^37,38^. Like other biofluids, UC has been the prevailing method to isolate EVs from milk. But the preprocessing for milk EV isolation involves stepwise elimination of impurities through seven steps of serial centrifugations and filtrations, which last over 6 h (ref. ^39^). Our hydrogel-based approach not only eliminated the need for tedious preprocessing, thereby saving isolation time by a factor of ~5.6 (Fig. 6a), but also exhibited a markedly superior performance in yield and purity, acquiring 1,539 times more nanoparticles from an identical volume of milk (Fig. 6b).

To demonstrate milk EVs’ therapeutic potential, we first applied them to human dermal fibroblasts (hDFs) in vitro. When we treated hDFs with an equal volume (1 ml) of milk EV isolates from an identical source volume (50 ml), as an identical-sample-volume analysis, EVs isolated by the meso–macroporous hydrogel increased hDFs’ proliferation by 22% compared with those isolated by UC and by 50% compared with the control without milk EVs (Fig. 6c). In an equal-protein-amount analysis (0.2 mg protein per millilitre culture media), milk EVs isolated by both hydrogel and UC enhanced hDFs’ proliferation (Extended Data Fig. 10a) and the expression of collagen II (Extended Data Fig. 10b) to almost the same extent.

To demonstrate the potential application of our methodology in therapeutic settings, we conducted functional experiments using human keratinocytes (HaCaT cells). By the equal-protein-amount analysis, we verified in-gel isolated milk EVs’ capability to stimulate cell proliferation and protect against oxidative damage. More specifically, milk EVs isolated by hydrogel promoted the proliferation of HaCaT cells (Extended Data Fig. 10c) and elevated the expression of glutathionylated proteins^38^ to a similar extent as milk EVs isolated by UC (Extended Data Fig. 10d). We observed indistinguishable cell proliferation and glutathionylated protein expression between HaCaT cells treated with EV-depleted milk (0.2 mg protein per millilitre) and non-treated cells (Extended Data Fig. 10e,f), validating with evidence that the proliferative and anti-oxidative effects originated from the milk EVs themselves rather than from contaminating proteins in the EV-depleted milk, consistent with previous observations^40,41^.

In the equal-particle-number analysis (5 × 10^8^ particles per millilitre), milk EVs isolated by the hydrogel and UC enhanced the proliferation of hDFs and HaCaT cells. Given that a corresponding protein concentration was ~6.7 times lower (0.03 mg ml^−1^) than that for the equal-protein-amount analysis, hydrogel-isolated EVs resulted in a slightly reduced cell proliferation (Fig. 6d,f). Also, the expression of collagen II by hDFs and the glutathionylated proteins by HaCaT cells corresponded to cell proliferation (Fig. 6e,g). These data indicate that milk EVs isolated by UC exhibit slightly better functionality than those isolated by hydrogel for cell proliferation, suggesting that not only the quantity but also the quality of isolated EVs is an important factor. Nevertheless, these results do not diminish the effectiveness of our method when considering the yield of EVs. Because the hydrogel-based isolation allowed for the acquisition of three orders of magnitude more EVs (Fig. 6b), our approach would lead to more favourable therapeutic outcomes by overcoming the insufficient nanoparticle numbers required to maximize EV functionality.

Urine is a readily available body fluid with relatively large volumes and has increasingly been adapted for early in vitro diagnostics. Simply by adding hydrogel particles, we successfully isolated EVs from urine (Fig. 6h), thereby validating the retrospective diagnosis of prostate cancer using human urinary EVs (Fig. 6i). Based on our previous work on EV microRNA (miRNA) detection^8^, we applied multiplex detection of miR-6090 and miR-3665 from lysates of off-gel recovered EVs. Using pooled urine samples (8 ml each from 12 subjects), the ratiometric profiling of the two EV miRNAs consistently showed a significant differentiation between prostate cancer patients and healthy controls (Fig. 6i and Supplementary Fig. 10).

## Conclusions

We have presented the development of meso–macroporous PEGDA hydrogel particles for the direct isolation of EVs, scalable to litres of source material. We achieved the unique pore characteristic by cryo-photocrosslinking PEG700DA without functionalization. Our exploitation of the gel-phase PEG, with only the addition of NaCl, enabled better or comparable performance in EV isolations, compared with currently established methodologies across various human body fluids, bovine milk and human stem cell culture media. Our method also applies to downstream analyses, including the two distinct but interconnected domains of diagnostics and therapeutics. This system offers several advantages over conventional isolation techniques, particularly regarding efficiency, scalability, accessibility, customizability and versatility (Supplementary Discussion).

Beyond batch processes for isolation, we could conceive of a column-based continuous process, filling the column with meso–macroporous hydrogel particles (that is, a packed-bed reactor). For this chromatography-combined application, the meso–macropores are crucial for the high EV specificity because we exploit the gel phase’s entire bulk (not just its outer surfaces), through which EVs should diffuse via the meso–macropores. While our technology offers the potential for industrial adaptation, the realization of mass fabrication of the meso–macroporous particles remains a challenge. Conversion from batch to continuous and automation of the cryo-photocrosslinking process would enable pilot studies in industrial settings, such as the development of EV-based therapeutics by contract development and manufacturing organizations. We believe that our meso–macroporous hydrogel technology will serve as a broadly impactful platform to encourage diverse EV studies, ranging from fundamental investigations to practical applications.

## Methods

### Fabrication of meso–macroporous PEGDA hydrogel particles

A PDMS (Sylgard 184; Dow Corning) mould with an array of wells was prepared by pouring a degassed mixture of PDMS and curing agent (10:1 w/w) onto a milling-machined duralumin master. After curing the PDMS mixture at 58 °C for 3 h, the PDMS replica was peeled off. Each well measured 5 mm in diameter and 2 mm in depth, corresponding to a volume of 40 µl per particle.

The PEGDA precursor solution comprised 10% (v/v) PEG700DA (catalogue no. 455008; Sigma-Aldrich), 88% (v/v) DI water and 2% (v/v) 2-hydroxy-2-methylpropiophenone (Irgacure 1173; catalogue no. 405655; Sigma-Aldrich) as a photoinitiator. For experiments in Extended Data Fig. 1a, the PEG700DA volume fraction was varied while maintaining a total composition of 98% (v/v) with DI water.

A volume of 40 µl of the precursor solution was dispensed into each PDMS well. To minimize temperature-induced freezing variations, the precursor-loaded mould was optionally chilled at 4 °C for 10 min. Freezing was then performed at –80 °C for 10 min, followed by immediate UV exposure in a chamber (MT-GJ09; Minuta) for 2 min at 2.5 mW cm^−2^ (300 mJ cm^−2^). The resulting cryo-photocrosslinked PEGDA particles were defrosted by rinsing with excess DI water for ~1 min. This step also facilitated washing out the photoinitiator and uncrosslinked PEG700DA and enabled harvesting of free meso–macroporous PEGDA particles.

The meso–macroporous hydrogel particles could be stored either dry (after lyophilization, recommended) or wet in DI water. For the lyophilization, particles were immersed in liquid nitrogen and freeze-dried overnight (TFD5503; IlShinBioBase).

Meso–macroporous hydrogel particles with a smaller volume (10 µl per particle) were fabricated by dispensing 10 µl droplets on parafilm and performing cryo-photocrosslinking as described above (Supplementary Fig. 8). After defrosting, these smaller particles spontaneously detached from the parafilm.

To produce hydrogel particles with tunable meso–macroporosity (Fig. 5d,e), the precursor solution was frozen in liquid nitrogen at –195 °C for 10 min, followed by the identical cryo-photocrosslinking procedure.

### Diffusion of fluorescent indicators to PEGDA particles

The porosity of meso–macroporous and microporous PEGDA particles was assessed indirectly by allowing 1D radial diffusion of fluorescent indicators into cylindrical PEGDA posts, constrained by impermeable top and bottom boundaries. Indicators included fluorescein (347.3 Da; ~1 nm; catalogue no. 201626; Sigma-Aldrich), FITC-dextran (10 kDa; 4.72 nm; catalogue no. FD10S; Sigma-Aldrich), green-fluorescent silica nanobeads (100 and 200 nm; catalogue nos. DNG-L015 and DNG-L019; CD Bioparticles) and custom-synthesized FITC-conjugated silica nanobeads (450 nm).

Cylindrical PEGDA posts (900 μm in diameter) were fabricated by photocrosslinking the precursor solution with or without the pre-freezing step, sandwiched between two 1-mm-thick glass slides separated by ~250 μm double-sided tape. The desired diameter was defined using a field diaphragm as a photomask on an inverted microscope (Axio Observer.A1; Zeiss). After cryo-photocrosslinking or standard photocrosslinking, PEGDA posts were rinsed with DI water to remove uncrosslinked polymer and photoinitiator.

Each fluorescent indicator was loaded into the gap between the glass slides and allowed to diffuse for 1 h, with the assembly sealed using parafilm to prevent evaporation. Fluorescence images were acquired as 16-bit monochrome images on the inverted microscope using an light-emitting diode (LED) light source (SOLA SM 2; Lumencor Light Engine), a filter cube set (89101x, 89101m and 89100bs; Chroma Technology) and a scientific complementary metal–oxide–semiconductor (sCMOS) camera (optiMOS; QImaging), with an exposure time of 10 ms.

### Direct isolation of EVs with meso–macroporous PEGDA hydrogel particles

EV isolation was performed using either an all-in-one tube or a tube-changeable approach (Fig. 2a). The protocol consisted of the following steps:

1. Prepare three required buffers:

- ×2 in-gel capture buffer (3 M NaCl, 40 mM HEPES in DI water)
- Wash buffer (1.5 M NaCl, 20 mM HEPES in DI water)
- Off-gel recovery buffer (20 mM HEPES in DI water, without NaCl)

In this study, the ×2 in-gel capture buffer was mixed at a 1:1 (v/v) ratio with the biofluid sample to yield a final concentration of 1.5 M NaCl and 20 mM HEPES, which implies that end users can readily adjust the concentration of the in-gel capture buffer on demand.

2. Prepare biofluid samples (human whole blood, plasma, saliva, urine, bovine milk or hESC/human induced pluripotent stem cell (hiPSC) culture media) in desired volumes.
3. Add meso–macroporous PEGDA particles at the following dosages:

- 4 particles ml^−1^ for whole blood, plasma and pre-concentrated hiPSC culture media (for Western blot)
- 1 particle ml^−1^ for saliva, urine, milk and ascites
- 0.25 particle ml^−1^ for hESC culture media

4. Add an equal volume of ×2 in-gel capture buffer to the biofluid sample, forming the EV isolation medium. It is recommended to add this buffer immediately before isolation begins.
5. Incubate the mixture at 4 °C for 1 h under rotation (10 rpm; B008DZ2VUG; finePCR).
6. Wash by aspirating the medium, adding wash buffer and rotating at 4 °C for 5 min. Wash volume was at least five times the total hydrogel-particle volume. Repeat this step three times.
7. Recover EVs by adding the off-gel recovery buffer and rotating for 5 min at 4 °C. This step removed residual high salt concentrations that could interfere with downstream analyses. End users can adjust the off-gel recovery buffer volume to achieve desired EV concentrations.
8. Collect EV isolates by extracting the recovery buffer or removing the hydrogel particles for downstream analyses.

For processing 1 l of ascites or milk, 1 particle ml^−1^ was used (that is, ~1,000 particles total). For smaller hydrogel particles (10 µl per particle), a dosage of 1 particle per 50 µl was used for plasma (Fig. 5b) and 4 particles per 200 µl for milk (Fig. 5c). To improve purity for the reduced isolation time shorter than 80 min (that is, 15 + 15 min; Extended Data Fig. 8c and Fig. 5c), 10 µl hydrogel particles were washed three times for 5 min each instead of <2 s.

For assessing hydrogel reusability (Extended Data Fig. 7), hydrogel particles previously used for plasma EV isolation were incubated in radioimmunoprecipitation assay (RIPA) buffer for 30 min at room temperature to disrupt the lipid bilayer membrane and soluble proteins. Particles were then washed thoroughly with DI water before reuse.

### Acquisition of biofluids

Whole blood and plasma were purchased as single-donor human whole blood (IWB1K2E; Innovative Research) and pooled human plasma (IPLAWBK2E; Innovative Research), both containing K2 EDTA as an anticoagulant.

Ascites samples were obtained from five gastric cancer patients under the Institutional Review Board (IRB) approval from Samsung Medical Center (IRB no. 2021-09-052). The study adhered to the principles of the Declaration of Helsinki and the Guidelines for Good Clinical Practice (ClinicalTrials.gov identifier, NCT02589496).

Commercial low-fat (15 mg ml^−1^) milk was sourced from Sangha Farm Organic.

Urine collection followed IRB-approved protocols at Korea University Anam Hospital (IRB no. 2017AN0036). All male participants underwent urological evaluation for elevated prostate-specific antigen (PSA) level, abnormal findings on digital rectal examination (DRE) or hypoechoic lesions in the prostate gland through transrectal ultrasonography. Urine was collected during initial admission for prostate biopsy after DRE. All the subjects underwent transrectal ultrasound-guided prostate biopsies and were classified as prostate cancer patients or healthy controls after pathologic examination of specimens. Samples from patients with urothelial carcinoma or other malignancies were excluded. Urine (5 ml) was centrifuged at 2,000*g* for 10 min at 4 °C, and 1.8 ml of the upper layer was stored at –80 °C. Samples were provided by the Biobank of Korea University Anam Hospital (KUAH2017-02).

Saliva was collected by swirling 12 ml of sterile DI water in the mouth for 1 min and expectorating into sterile tubes. Large debris (for example, food residues) was removed by centrifugation at 2,000*g* for 10 min at 4 °C. The upper 10 ml was stored at –80 °C before EV isolation.

**Extended Data Fig. 3:**
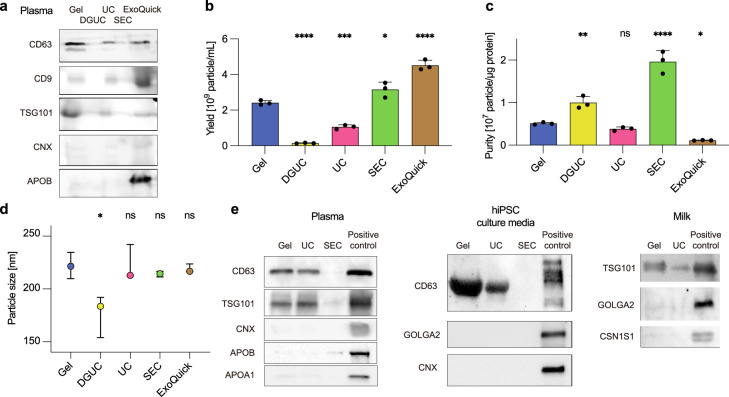
Characterizations for human plasma EVs isolated with meso–macroporous hydrogel. **a**, Western blot images showing the expression of EV-positive (CD63, CD9 and TSG101) and EV-negative (CNX and APOB) markers from the isolated nanoparticles by meso–macroporous hydrogel and other conventional methods: density gradient ultracentrifugation (UC), UC, size exclusion chromatography and ExoQuick. **b**,**c**, Yield (**b**) and purity (**c**) of nanoparticles isolated by the four methodologies. Statistical significance of the conventional methods to hydrogel, ****P < 0.0001, ***P = 0.0002 and *P = 0.0122 for yield (**b**); **P = 0.0045, NS *P* = 0.6296, ****P < 0.0001 and *P = 0.0164 for purity (**c**). Error bars indicate mean ± s.d. (technical replicates, *n* = 3). **d**, Median particle size and its range. Statistical significance of the conventional methods to hydrogel, *P = 0.0116, NS *P* > 0.9999 (UC), = 0.9444 (SEC) and 0.9985 (ExoQuick). Plots display the median size (circle) and the whiskers indicating the minimum and maximum values (technical replicates, *n* = 3). **e**, Western blot images showing the expression of EV-positive (CD63 and TSG101) and EV-negative (CNX, APOB, APOA1, GOLGA2 and CSN1S1) markers in nanoparticles isolated from human plasma (left), hiPSC culture media (middle) and bovine milk (right) by meso–macroporous hydrogel, UC, size exclusion chromatography and positive controls. Source data

**Extended Data Fig. 4:**
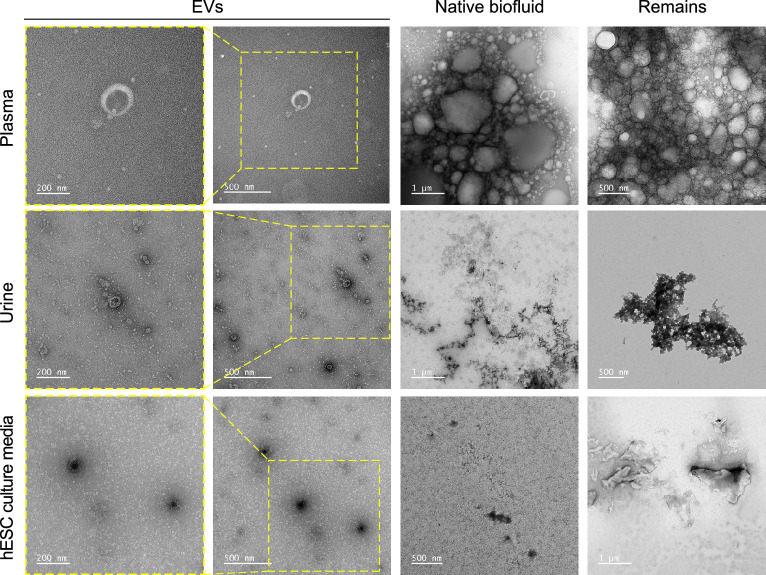
Negative-stain TEM to visually identify contaminants. Negative-stain TEM images showing EVs isolated with meso–macroporous hydrogel (columns 1 and 2), native biofluid before isolation (column 3) and remains of the sample left over after isolation (column 4) for human plasma (top), urine (middle) and hESC culture media (bottom).

For EV isolation from stem cell culture media, the H1 hESC line (H1ESC) and hiPSC line (WTC-11; GM25256; Coriell Institute for Medical Research) were cultured in six-well plates on Matrigel-coated surfaces (1:25 dilution in DMEM F-12; catalogue no. 11320033; Gibco). Cultures were maintained in mTeSR1 (a feeder- and serum-free maintenance medium for hESCs and hiPSCs; catalogue no. 85850; STEMCELL Technologies) at 37 °C and 5% CO_2_. Culture medium was harvested after 5–7 days, when H1ESC colonies reached ~0.5 mm in diameter. Media were collected without further washing, distributed into 15 ml tubes and stored at –80 °C before EV isolation. For Western blotting (Extended Data Fig. 3e), 130 ml of hiPSC culture media was concentrated to 35 ml using a 10 kDa molecular weight cut-off (MWCO) centrifugal filter (Amicon Ultra; Merck Millipore).

### EV preservation in hydrogel carriers

To evaluate the potential of meso–macroporous PEGDA hydrogel particles as EV-preserving carriers, the EV isolation protocol (steps 1–6) for milk was performed. Instead of immediate recovery, EV-captured hydrogel particles were lyophilized overnight and stored at room temperature for up to 60 days. EVs were subsequently recovered, and size distribution, yield and purity were analysed by nanoparticle tracking analysis and Bradford assay.

### Milk EV treatment of human cells and downstream analyses

The potential cosmeceutical (therapeutic) effects of milk EVs were assessed in primary dermal fibroblasts (hDFs; PCS-201-012; American Type Culture Collection (ATCC)) and keratinocytes (HaCaT cells; CRL-2404; ATCC). Cells were plated at ~2 × 10^5^ hDFs in 35 mm (diameter) confocal dishes for collagen II staining, or ~5 × 10^5^ HaCaT cells for glutathionylated protein analysis. Cells were cultured in DMEM (CM002-050; GenDEPOT) with 10% (v/v) foetal bovine serum (16000044; Gibco) and 1% (v/v) antibiotic–antimycotic (CA002-010; GenDEPOT) at 37 °C and 5% CO_2_ for 12 h (hDFs) or 24 h (HaCaT cells). Culture media were replaced with fresh media containing either hydrogel-isolated or UC-isolated milk EVs, or EV-depleted milk, for analyses based on either equal protein concentration (0.2 mg ml^−1^) or equal particle concentration (5 × 10^8^ particles ml^−1^). Following a further 24 h incubation, cells were washed with phosphate-buffered saline (PBS).

For immunostaining, EV-treated hDFs were fixed with 4% (w/v) paraformaldehyde for 10 min, washed three times with ice-cold PBS, permeabilized with 0.25% (v/v) Triton X-100 in PBS (PBST) for 5 min, blocked with 1% (w/v) bovine serum albumin in PBST for 30 min and incubated overnight at 4 °C with anti-collagen II primary antibody (ab34712; Abcam; 1:500). Following washes with PBST, cells were incubated with secondary antibody (ab205718; Abcam; 1:5,000) for 1 h in the dark at room temperature. Fluorescence imaging was performed on a confocal microscope (Leica TCS SP6; Leica).

For Western blotting, EV-treated HaCaT cells were lysed in RIPA buffer (89900; Thermo Fisher Scientific) with 1% (v/v) protease inhibitor at 4 °C for 30 min. Lysates were centrifuged at 14,000*g* for 15 min, and protein content was determined by bicinchoninic acid (BCA) assay (23227; Thermo Fisher Scientific). Proteins were denatured in sodium dodecyl sulfate–polyacrylamide gel electrophoresis (SDS-PAGE) loading buffer (SF2002-110-00; Biosesang) and separated in 10% (w/v) SDS–polyacrylamide gels. After transfer to nitrocellulose membranes, blocking was performed with 5% (w/v) skim milk at 25 °C for 30 min. Membranes were incubated overnight at 4 °C with primary antibodies (glutathione, ab19534; GAPDH, ab9485; Abcam; both 1:1,000), rinsed several times with Tris-buffered saline with 0.1% (w/v) Tween 20 detergent for 15 min. Washed membranes were incubated in 5% (w/v) skim milk with horseradish-peroxidase-tagged secondary antibodies (1706516, 1706515; Bio-Rad; both 1:1,000) at 25 °C for 1 h. Bands were visualized using a luminescent image analyser (iBright CL750; Invitrogen).

For cell proliferation assays, hDFs and HaCaT cells were plated in 96-well plates at 5 × 10^3^ and 1 × 10^4^ cells per well, respectively. Following the same EV treatments, media were replaced and 10% (v/v) CCK-8 solution (CK04; Dojindo) was added for 60 min. Absorbance was measured at 450 nm using a microplate reader (SpectraMax 34; Molecular Devices).

EV-depleted milk was prepared by serial centrifugation and UC at 4 °C, followed by filtration. Milk fat globules, somatic cells and cell debris were removed by centrifugation at 5,000*g* for 30 min and 12,000*g* for 1 h. Defatted milk was stored at –80 °C before use. Upon thawing, residual fat and casein were removed by UC at 35,000*g* for 1 h and 70,000*g* for 3 h (Optima XE-100 Ultracentrifuge; Beckman Instruments). Supernatant was sequentially filtered through 0.80, 0.45 and 0.2 μm filters (Sartorius) and further ultracentrifuged at 100,000*g* for 1 h and 200,000*g* for 3 h to yield EV-depleted milk.

### Urinary EV miRNA profiling by hydrogel-based hybridization chain reaction

Multiplex profiling of urinary EV miRNAs (miR-6090 and miR-3665) was performed by hydrogel-based hybridization chain reaction (HCR) as previously described^8^, with modified sample preparation. Urine from 12 individuals per group (prostate cancer and healthy) was pooled (8 ml per group). EVs were isolated with meso–macroporous hydrogel particles at a 1 particle ml^−1^ dosage. Total RNA was extracted and dissolved in 10 μl nuclease-free water with TRIzol (Invitrogen).

The 10 µl of urinary EV RNAs were mixed with shape-encoded conventional intraplex PEGDA microparticles, hybridized at 55 °C for 90 min and ligated to biotinylated adaptors at 21.5 °C for 30 min. Neutravidin was bound at 21.5 °C for 90 min. HCR amplification was performed by adding biotinylated universal initiators and pairs of biotinylated hairpins at 37 °C for 4 h, followed by streptavidin–phycoerythrin tagging at 21.5 °C for 45 min. The entire HCR was performed in a thermal shaker with agitation at 1,500 rpm.

Fluorescence images (16-bit monochrome) were acquired on an inverted microscope (Axio Observer.A1; Zeiss) equipped with a SOLA SM 2 LED, Chroma filter cubes and an optiMOS sCMOS camera. Ratiometric fluorescence intensity (*MIR6090*/*MIR3665*) was analysed to distinguish prostate cancer samples from healthy controls.

### Statistical analysis

All statistical analyses were performed in Prism (GraphPad Software). Analysis of variance (ANOVA) was used for comparisons involving more than two groups (Figs. 4b, 5a and 6c,d,f, Extended Data Figs. 1a–g, 2c, 3b–d and 10a,c and Supplementary Figs. 5d,f and 7b), and *t*-tests were used for comparisons between two groups (Figs. 2b, 3e, 4d, 5b–e,g and 6b,h,i, Extended Data Figs. 7b, 8b,c, 9 and 10e and Supplementary Figs. 1, 4b and 6). Parametric two-tailed unpaired *t*-tests and one-way ANOVA with Dunnett’s multiple comparisons were applied. Statistical significance was denoted as NS (*P* > 0.05), *(*P* < 0.05), ***(*P* < 0.001) and ****(*P* < 0.0001). Full statistical details appear in Supplementary Table 4. No statistical methods were used to predetermine sample sizes, but our sample sizes were similar to those reported in previous publications^23^. Data distribution was assumed to be normal, but this was not formally tested. Samples were randomly assigned to experimental groups, and experiments were performed in randomized order to minimize bias. Data collection and analysis were not performed blind to the conditions of the experiments.

### Statistics and reproducibility

For imaging experiments (Figs. 1c, 2d, 4a and 6e, Extended Data Figs. 4 and 10b and Supplementary Figs. 2, 3, 4a, 7a and 10), at least three images were acquired with consistent results. Representative images are shown in the figures.

### Reporting summary

Further information on research design is available in the Nature Portfolio Reporting Summary linked to this article.

## Online content

Any methods, additional references, Nature Portfolio reporting summaries, source data, extended data, supplementary information, acknowledgements, peer review information; details of author contributions and competing interests; and statements of data and code availability are available at 10.1038/s41565-025-02011-1.

## Supplementary information

Supplementary Information Supplementary Figs. 1–10, Tables 1–4, Results, Discussion and Methods.

Reporting Summary

## Source data

Source Data Fig. 2 Statistical source data.

Source Data Fig. 3 Statistical source data.

Source Data Fig. 3 Unprocessed western blots.

Source Data Fig. 4 Statistical source data.

Source Data Fig. 5 Statistical source data.

Source Data Fig. 6 Statistical source data.

Source Data Fig. 6 Unprocessed western blots.

Source Data Extended Data Fig. 1 Statistical source data.

Source Data Extended Data Fig. 2 Statistical source data.

Source Data Extended Data Fig. 3 Statistical source data.

Source Data Extended Data Fig. 3a,e Unprocessed western blots.

**Extended Data Fig. 5:**
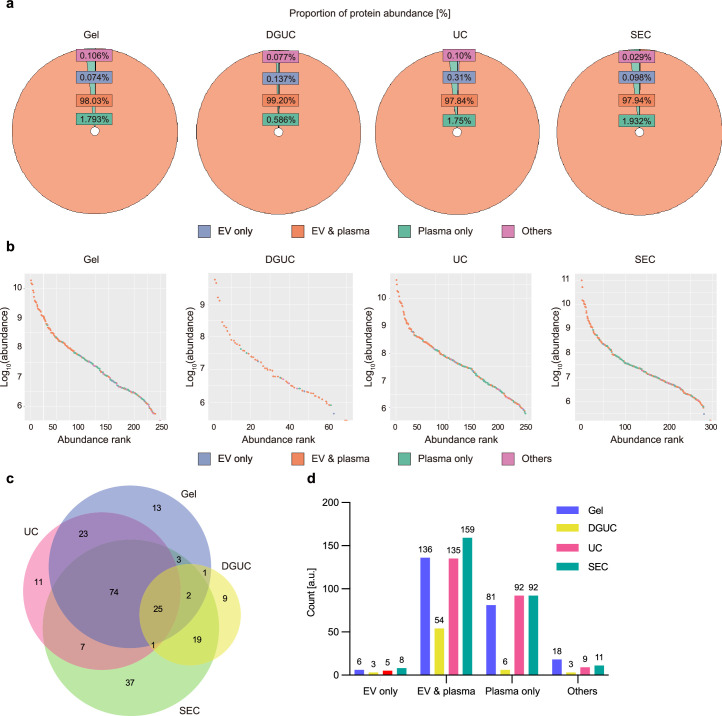
Proteomic analysis of isolates acquired by meso–macroporous hydrogel and conventional methodologies. **a**,**b**, Doughnut charts (**a**) and abundance rank plots (**b**) for proteins identified and classified in four categories: EV only, EV & plasma, plasma only and others. **c**, Number of proteins classified in the four categories over various methodologies, including the meso–macroporous hydrogel-based isolation. **d**, Venn diagram displaying proteomic composition (that is, the number of EV proteins) of EVs isolated by meso–macroporous hydrogel, density gradient UC, UC and SEC. Source data

**Extended Data Fig. 6:**
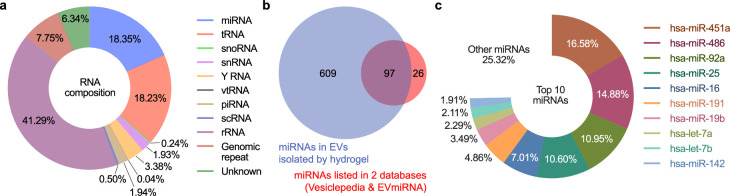
RNA sequencing of human plasma EVs isolated by meso–macroporous hydrogel. **a**, Doughnut chart displaying the composition of plasma EV RNAs. **b**, Venn diagram showing the number of miRNAs in EVs isolated by hydrogel (blue) and those listed in two databases of Vesiclepedia and EVmiRNA (red). **c**, Doughnut chart displaying plasma EV miRNAs with top 10 reads. Source data

Source Data Extended Data Fig. 5 Statistical source data.

Source Data Extended Data Fig. 6 source data for plasma EV-RNA sequencing reads.

**Extended Data Fig. 7:**
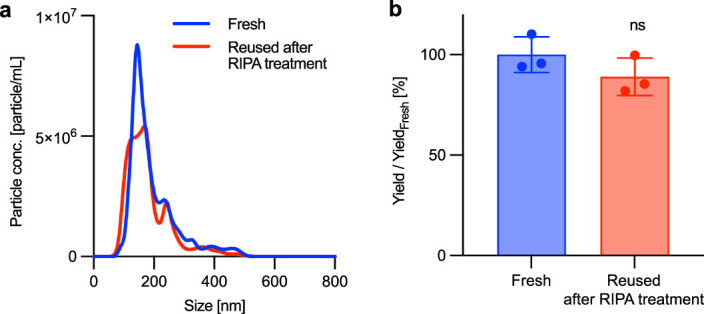
Reusability of meso–macroporous hydrogel particles for EV isolation. **a**,**b**, Size distribution (**a**) and relative yield (Yield / Yield_Fresh_; **b**) of EVs isolated by fresh (blue) and reused hydrogel particles after RIPA treatment (red). Statistical significance, *P* = 0.2139. Error bars indicate mean ± s.d. (technical replicates, *n* = 3). Source data

Source Data Extended Data Fig. 7 Statistical source data.

**Extended Data Fig. 8:**
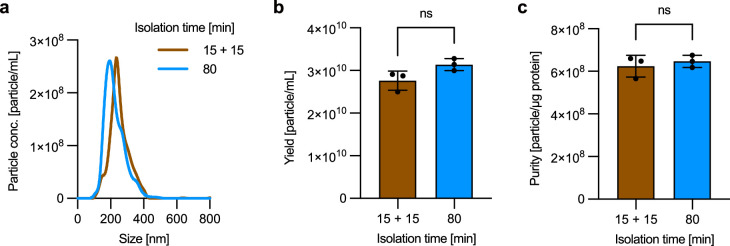
Enhancement of purity for reduced isolation time. **a**–**c**, Size distribution (**a**), yield (**b**) and purity (**c**) of EVs isolated by 10 µL-hydrogel particles for 15 min with extended wash steps (three times each for 5 min; brown) and 80 min (blue). Statistical significance, NS *P* = 0.1096 (**b**) and 0.7830 (**c**). Error bars indicate mean ± s.d. (technical replicates, *n* = 3). Source data

Source Data Extended Data Fig. 8 Statistical source data.

**Extended Data Fig. 9:**
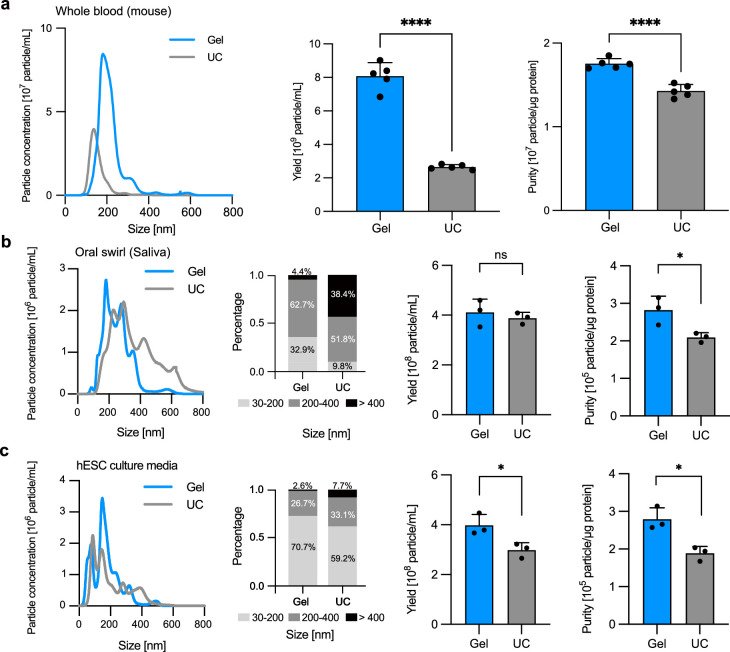
Direct EV isolation from various biofluids. **a**, Size distribution, yield and purity of mouse whole blood EVs isolated by hydrogel (blue) and UC (grey). Statistical significance, ****P < 0.0001. Error bars indicate mean ± s.d. (technical replicates, *n* = 5). **b**,**c**, EV isolations from human saliva (**b**) and hESC culture media (**c**) by hydrogel (blue) and UC (grey): size distribution, percentage of the area under the size distribution curves between 30 and 400 nm, yield and purity. Statistical significance, *P = 0.0304 (yield in **b**) and *P = 0.0117 (purity in **b**) for hESC cell culture media; NS *P* = 0.5364 (yield in **c**) and *P = 0.0316 (purity in **c**) for saliva. Error bars indicate mean ± s.d. (technical replicates, *n* = 3). Source data

Source Data Extended Data Fig. 9 Statistical source data.

**Extended Data Fig. 10:**
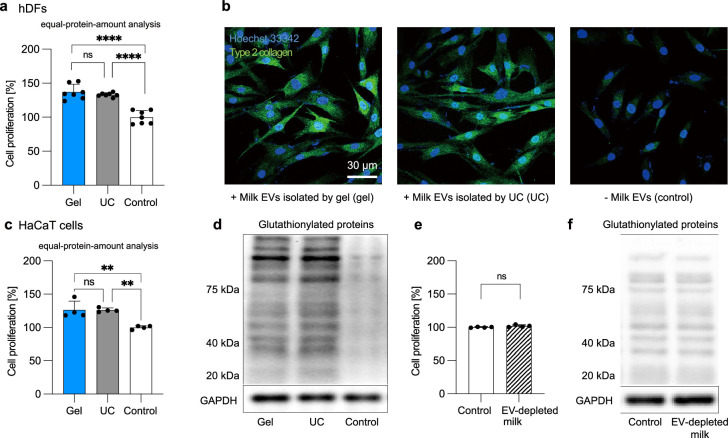
Equal-protein-amount analyses for bovine milk EVs’ cosmeceutical (therapeutic) potential. **a**, Equal-protein-amount analysis for the proliferation of human dermal fibroblasts (hDFs) at three days in vitro (DIV 3) after treating milk EVs isolated by hydrogel (blue) and UC (grey) for 24 h, relative to proliferation without milk EVs (white). Statistical significance NS *P* = 0.6465 and ****P < 0.0001. Error bars indicate mean ± s.d. (technical replicates, *n* = 7). **b**, Fluorescence images showing hDFs’ nuclei (blue) and expression of collagen II (green), depending on the treatment of milk EVs isolated by hydrogel (left), UC (middle) and no treatment (right) as part of the equal-protein-amount analysis. **c**, Equal-protein-amount analysis for the proliferation of human keratinocytes (HaCaT cells) at DIV 3 after treating milk EVs isolated by hydrogel (blue), UC (grey) for 24 h, relative to proliferation without milk EVs (white). Statistical significance, NS *P* > 0.9994 (gel-UC), **P = 0.0031 (gel-control) and 0.0033 (UC-control). Error bars indicate mean ± s.d. (technical replicates, *n* = 4). **d**,**f**, Western blot image showing glutathionylated proteins and GAPDH expression by HaCaT cells. **e**, Proliferation of HaCaT cells at DIV 3 after treating EV-depleted milk (hatched) for 24 h, relative to proliferation without milk EVs (white). Statistical significance, NS *P* = 0.0844. Error bars indicate mean ± s.d. (technical replicates, *n* = 4). Source data

Source Data Extended Data Fig. 10 Statistical source data.

Source Data Extended Data Fig. 10d,f Unprocessed western blots.

## Data Availability

The data supporting this study’s findings are available from the manuscript, its Supplementary Information or the corresponding authors upon request. The proteomic dataset for EV proteins in human plasma analysed by liquid chromatography–tandem mass spectrometry (LC–MS/MS) is available via Zenodo at 10.5281/zenodo.15794986 (ref. ^42^). The dataset for EV RNAs in human plasma analysed by RNA sequencing is available via Zenodo at 10.5281/zenodo.15796138 (ref. ^43^). Source data are provided with this paper.
