# Supplementary material for: Meso–macroporous hydrogel for direct litre-scale isolation of extracellular vesicles
Source: Nat Nanotechnol. 2025 Sep 24;20(11):1678–87. doi: 10.1038/s41565-025-02011-1 (PMC12623240; doi:10.1038/s41565-025-02011-1)
Supplement: Supplementary file 1 — Supplementary Figs. 1–10, Tables 1–4, Results, Discussion and Methods. [file 41565_2025_2011_MOESM1_ESM.pdf]

nature nanotechnology

Supplementary information

https://doi.org/10.1038/s41565-025-02011-1

Meso–macroporous hydrogel for direct litre-scale isolation of extracellular vesicles

In the format provided by the authors and unedited

Table of contents

Supplementary Figures 1
- Supplementary Fig. 1 1
- Supplementary Fig. 2 2
- Supplementary Fig. 3 3
- Supplementary Fig. 4 4
- Supplementary Fig. 5 5
- Supplementary Fig. 6 6
- Supplementary Fig. 7 7
- Supplementary Fig. 8 8
- Supplementary Fig. 9 9
- Supplementary Fig. 10 10

Supplementary Tables 11
- Supplementary Table 1 11
- Supplementary Table 2 12
- Supplementary Table 3 13
- Supplementary Table 4 14

Supplementary Results 16

Supplementary Discussion 21

Supplementary Methods 26

References 33

Supplementary Figures

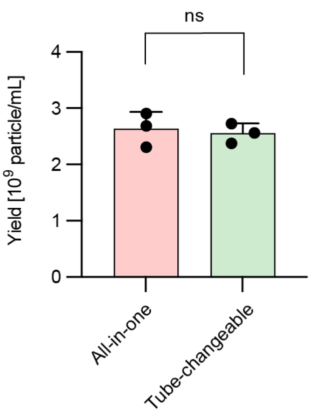

Supplementary Fig. 1 | Performance comparison between the all-in-one tube and tube-changeable EV isolations. Yield of the all-in-one tube (pink) and tube-changeable (green) isolations from human plasma. Statistical significance: ns*P* = 0.7171. Error bars indicate mean ± s.d. (technical replicates: the number of hydrogel particles, *n* = 3).

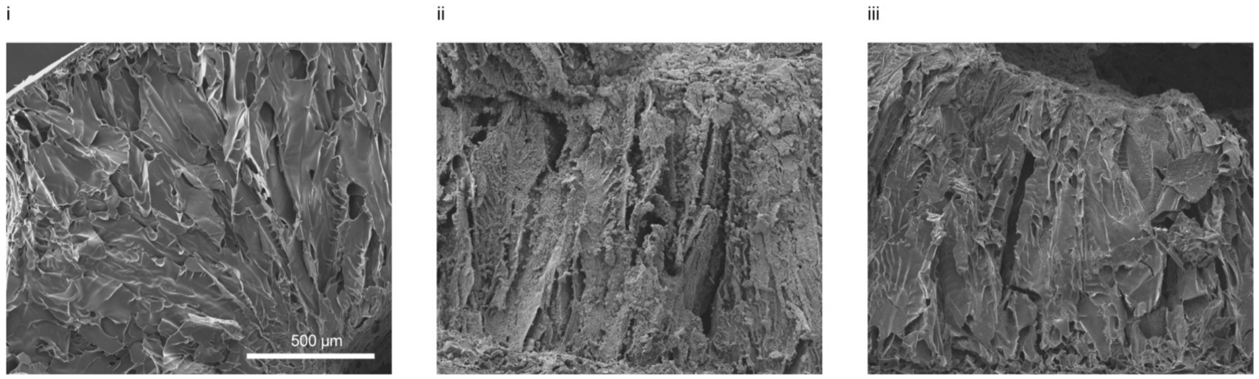

Supplementary Fig. 2 | Scanning electron microscopy (SEM) of cross-sections of meso-macroporous hydrogel particles during EV isolation. Each SEM image represents a cross-section of meso-macroporous particles before EV isolation (i), after in-gel capture of EVs (ii), and after off-gel recovery of EVs (iii). All images shown are representative of three independent experiments with consistent results.

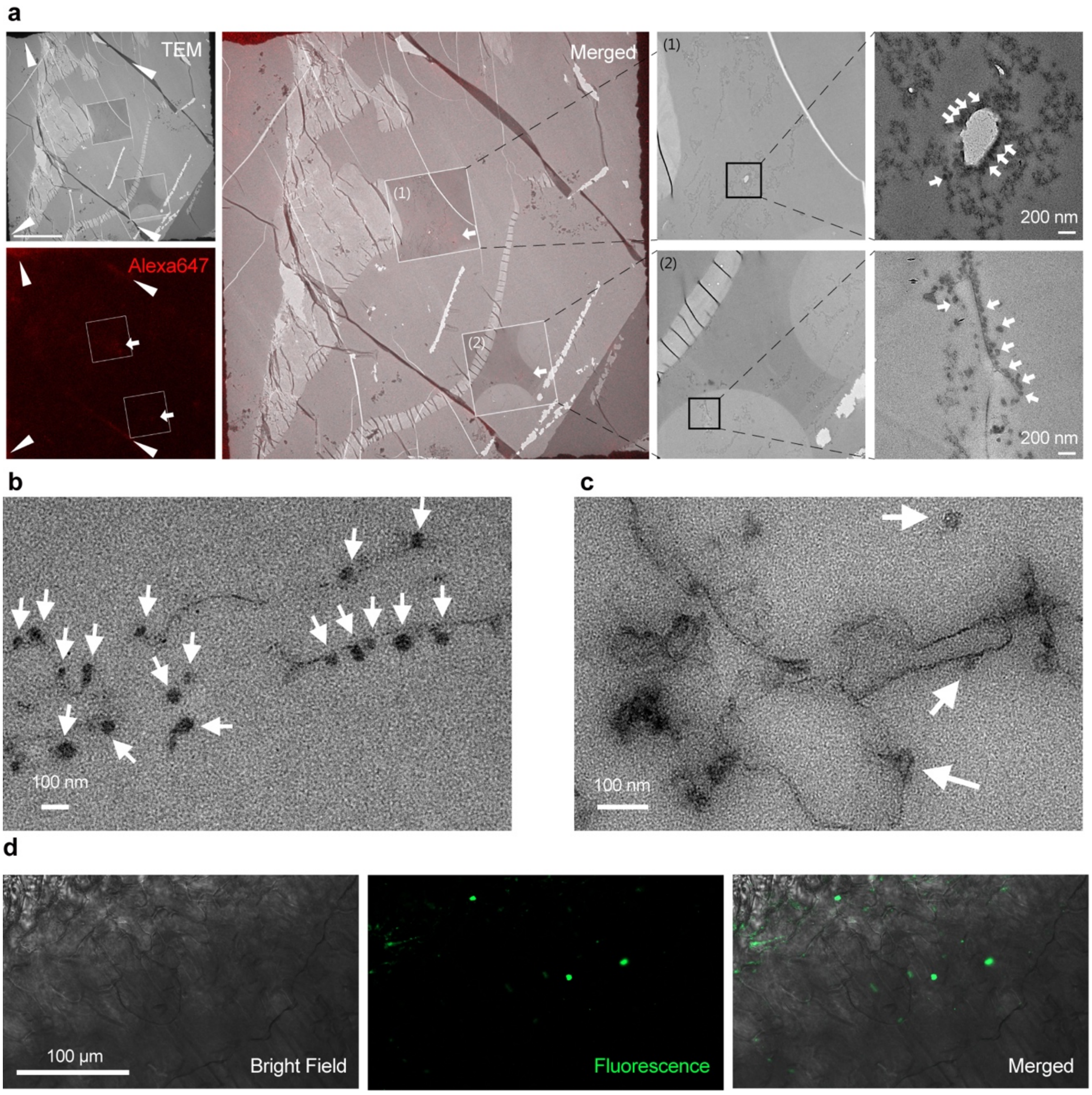

Supplementary Fig. 3 | Correlative light-electron microscopy (CLEM), cryo-transmission electron microscopy (cryo-TEM), and fluorescence imaging of EV-captured meso-macroporous hydrogel *in situ*. a, Representative CLEM images showing a cross-section of meso-macroporous hydrogel after in-gel capture of EVs immunostained with anti-CD63 conjugated with Alexa 647 (red). Triangular arrows without tails are on-image align markers for spatial correlation. Arrows indicate EVs. **b** and **c**, Representative cryo-TEM images showing a cross-section of meso-macroporous hydrogel after in-gel capture of EVs, fixed and stained with uranyl acetate (**b**) and osmium tetroxide (OsO_4; **c**). Arrows indicate EVs captured within the hydrogel. **d**, Representative fluorescence images showing DiO (green)-stained EVs captured within the hydrogel. All images shown are representative of three independent experiments with consistent results.

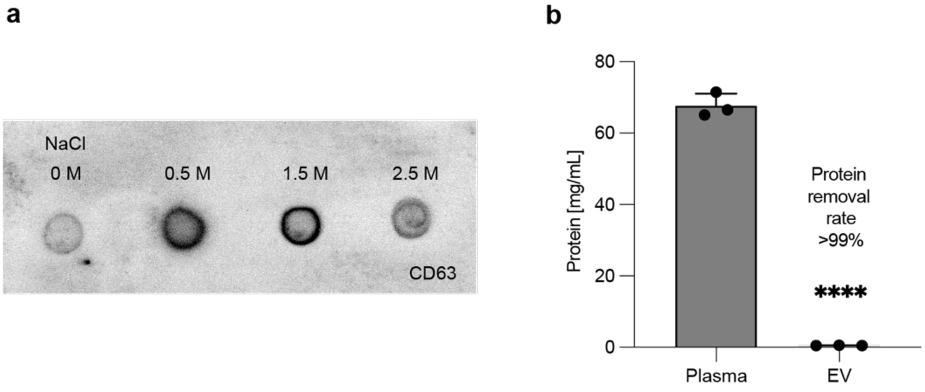

Supplementary Fig. 4 | Optimization of operational parameters for EV isolation with meso-macroporous hydrogel particles. a, Dot blot image showing expression of the EV-positive marker, CD63, in isolates acquired from human plasma by varying NaCl concentration. The image shown is representative of three independent experiments with consistent results. **b**, Concentration of proteins in human plasma (left) and off-gel recovered isolates (right). Statistical significance: ****$P < 0.0001$. Error bars indicate mean ± s.d. (technical replicates, $n = 3$).

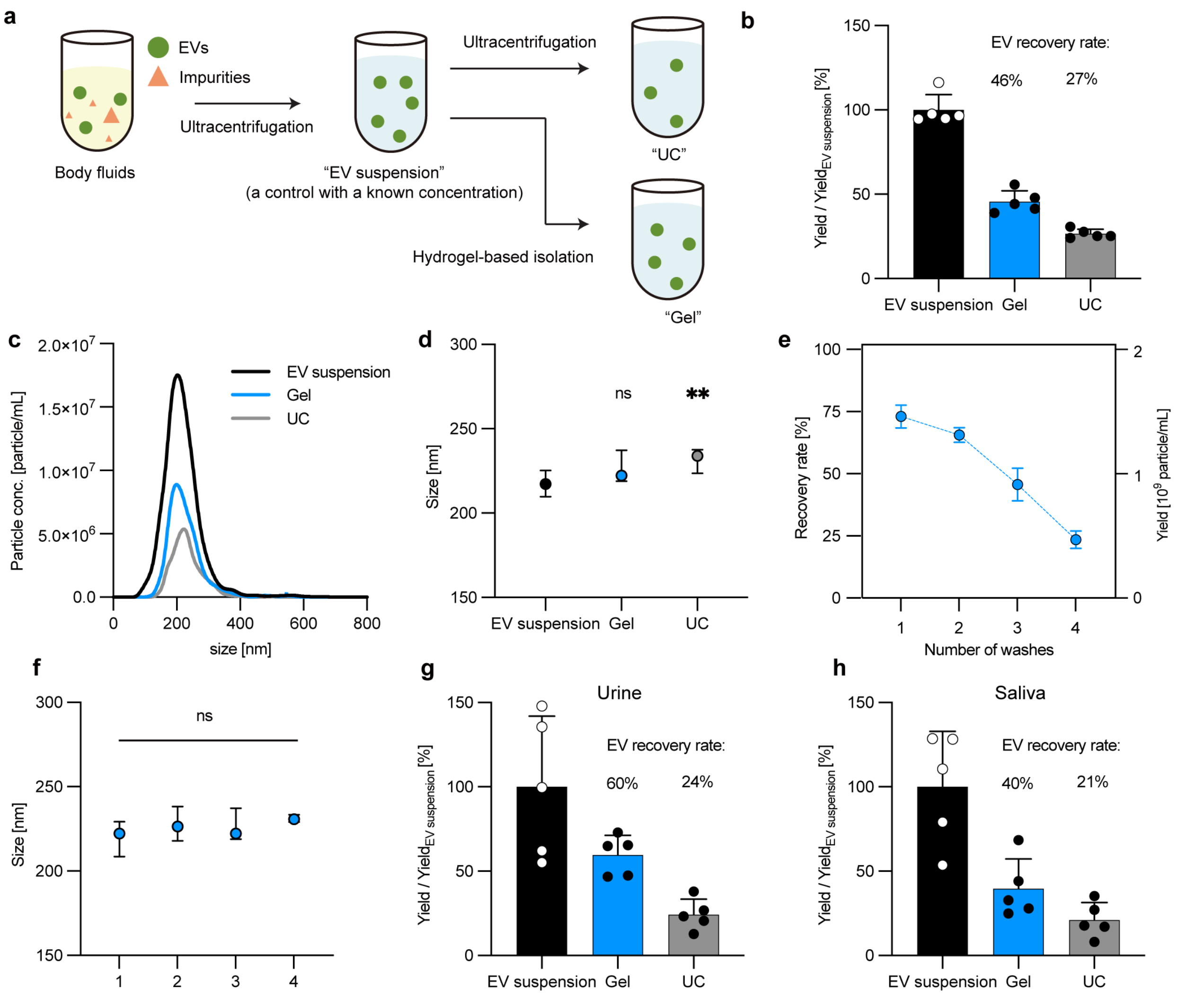

Supplementary Fig. 5 | Comparison of EV recovery rate. a, Schematic overview depicting a process for estimating the EV recovery rate. **b** - **d**, Relative yield (Yield / Yield$_{\text{EV suspension}}$) with EV recovery rate (**b**), size distribution (**c**), and median particle size (**d**) of EV suspension (i.e., known standard; black), off-gel recovered (blue), and ultracentrifugation (UC)-isolates (gray) from human plasma. Statistical significance to the EV suspension: ns$P$ = 0.0723, and **P = 0.0077. Error bars indicate mean ± s.d. (**b**) and a range from minimum to maximum sizes (**d**) (technical replicates, n = 5). **e**, Recovery rate and yield depending on the number of washes. Error bars indicate mean ± s.d. (technical replicates, n = 5). **f**, Median particle size depending on the number of washes. Statistical significance to three washes: ns$P$ = 0.4171 (1 wash), > 0.9999 (2 washes), and 0.8180 (4 washes). Error bars indicate a range from minimum to maximum sizes (technical replicates, n = 5). **g** and **h**, Relative yield (Yield / Yield$_{\text{EV suspension}}$) with EV recovery rate from human urine (**g**) and saliva (**h**). Error bars indicate mean ± s.d. (technical replicates, n = 5).

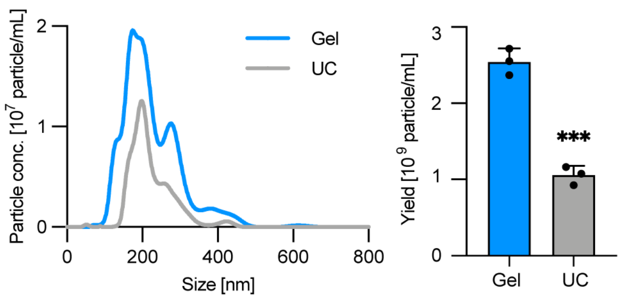

Supplementary Fig. 6 | Comparison of yield. Size distribution (left), and yield (right) of EVs isolated by hydrogel particles (blue) and ultracentrifugation (UC; gray). Statistical significance: ***P = 0.0003. Error bars indicate mean ± s.d. (technical replicates, n = 3).

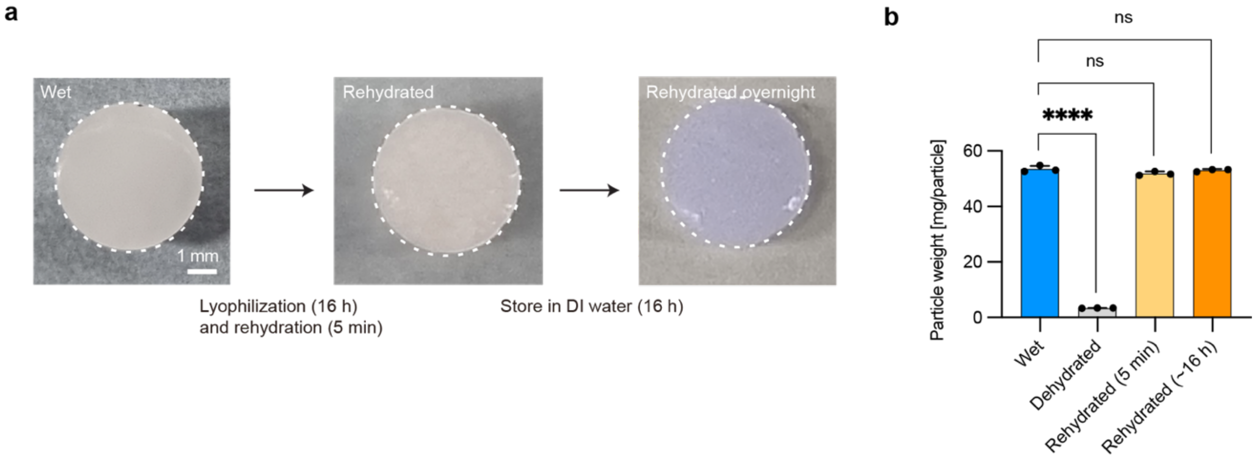

Supplementary Fig. 7 | Swelling and EV isolation performance of meso-macroporous hydrogel particles upon dehydration and rehydration. a, Photographs of a meso-macroporous hydrogel particle in three states: wet (left), rehydrated for 5 min after lyophilization (middle), and rehydrated for an additional 16 h (right). All images shown are representative of three independent experiments with consistent results. **b**, Weight of meso-macroporous hydrogel particles in the wet (blue), dehydrated (gray), rehydrated (for 5 min; yellow), and rehydrated (for 16 h; orange) states. Statistical significance to the wet state: ****$P < 0.0001$ (dehydrated), ns$P = 0.0614$ (rehydrated for 5 min), and 0.7781 (rehydrated for 16 h). Error bars indicate mean ± s.d. (technical replicates: $n = 3$).

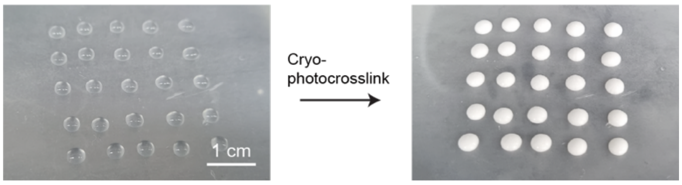

Supplementary Fig. 8 | Further reduction of volumes of meso-macroporous hydrogel particles. Photographs showing a 5×5 array of 10% [v/v] PEG700DA precursor drops (10 μL each) before (left) and after (right) cryo-photocrosslinking. All images shown are representative of three independent experiments with consistent results.

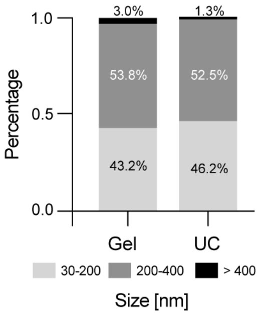

Supplementary Fig. 9 | Direct EV isolation from whole blood with meso-macroporous hydrogel. Percentage of the area under the size distribution curves (Fig. 5e) between 30 and 400 nm.

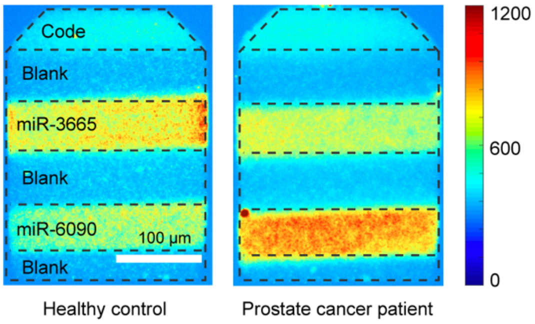

Supplementary Fig. 10 | Demonstration of downstream analysis: EV-miRNA profiling for diagnosis of prostate cancer. Representative fluorescence images of shape-encoded intraplex PEGDA hydrogel microparticles displaying multiplex detection of human urinary EV-miR-3665 and -6090 by hydrogel-based hybridization chain reaction (HCR) for signal amplification from healthy controls (left) and prostate cancer patients (right). All images shown are representative of 26 independent experiments with consistent results.

Supplementary Tables

Supplementary Table 1 | Costs of materials to fabricate a meso-macroporous hydrogel particle

Material	Catalog #	Amount	Price [USD]	Price per amount [USD/mL or g]
PEG700DA	455008-500ML	500 mL	138.24	0.28
Irgacure 1173	405655-250ML	250 mL	221.73	0.89
PEG1000DA	729086-1g	1 g	166.89	166.89
**Product**	**Composition**	**Price per volume [USD/mL]**	**Particle volume**	**Price per particle [USD/particle]**
Meso-macroporous hydrogel particle	PEG700DA 10% [v/v] + Irgacure 1173 2% [v/v]	0.045	40 µL	0.0018

Supplementary Table 2 | Information on clinical samples used in this study

Patient #	Cancer type	Ascites volume [L]	Sex	Age
1	Advanced gastric cancer (AGC)	1	Female	55
2	AGC	1	Male	62
3	AGC	1	Female	58
4	AGC	1	Male	50
5	AGC	1	Male	73

Supplementary Table 3 | Comparison of featured capabilities for EV isolation methods

	Meso-macroporous hydrogel	Ultra-centrifugation	Size exclusion chromatography (SEC)	Precipitation	Tangential flow filtration (TFF)
**Liter-scale isolation**	**O**	X	X	X	O
**Pre-processing -free**	**O**	X	X	X	X

Supplementary Table 4 | Statistical details

Figure	*t* value / *F* value	Degree of freedom	95% confidence interval	R^2
2b	39.61	8	-101.9 to -90.72	0.9949
3d	49.41	4	2.766e+13 to 3.096e+13	0.9984
3e (purity)	1.077	4	-80749521 to 35618444	0.2247
3e (yield)	2.033	4	-4835065473 to 747598813	0.5081
4b	0.1574	2,6	-	0.04984
4d	0.8043	8	-24768712 to 51299390	0.07481
5a (yield)	203.3	2,6	-	0.9855
5a (amount)	5.621	2,6	-	0.6520
5b	1.632	2.959	-0.2813 to 0.8644	0.4738
5c (purity)	2.992	4	-1.60e+08 to -5.96e+06	0.6911
5c (yield)	2.232	4	-6.89e+09 to 7.49e+08	0.5546
5d	4.965	8	-101.3 to -37.04	0.755
5e (purity)	0.1472	8	-1370151 to 1205754	0.0027
5e (yield)	2.883	8	-1.53e+09 to -1.70e+08	0.5096
5g (purity)	0.2726	4	-2.27e+06 to 2.76e+06	0.01824
5g (yield)	6.19	4	-1.49e+10 to -5.68e+09	0.9055
6b	24.98	4	-6.05e+08 to -4.84e+08	0.9936
6b (yield)	38.64	4	-3.36e+10 to -2.91e+10	0.9973
6c	105.1	2,9	-	0.9589
6d	15.17	2,18	-	0.6277
6f	96.36	2,9	-	0.9554
6h	0.07185	4	-2.47e+06 to 2.34e+06	0.001289
6i	21.46	50	1.016 to 1.226	0.902
E1a	54.59	4, 10	-	0.9562
E1b	0.9853	3,12	-	0.1976
E1c	36.70	3, 8	-	0.9323
E1d	49.59	2, 6	-	0.943
E1e	3.161	2,6	-	0.6599
E1f (purity)	34.05	4, 10	-	0.9316
E1f (yield)	94.82	4, 10	-	0.9743
E1g	2.231	3,8	-	0.4555
E2c	1.076	2, 12	-	0.1521
E3b	153.1	4, 10	-	0.9839
E3c	85.48	4, 10	-	0.9716

Figure	*t* value / *F* value	Degree of freedom	95% confidence interval	R^2
E7b	1.476	4	-31.72 to 9.698	0.3527
E8b	2.463	4	-8040834999 to 480771398	0.6027
E8c	0.6627	4	-116889269 to 71840165	0.09894
E9a (purity)	7.47	8	-4256415 to -2248288	0.8746
E9a (yield)	14.91	8	-6265021267 to -4586745836	0.9653
E9b (purity)	3.244	4	10479 to -4586745836	0.7246
E9b (yield)	0.6755	4	-70738902 to 116230089	0.1024
E9c (purity)	4.4	4	33523 to 148162	0.8288
E9c (yield)	0.3157	4	-74601668 to 93741564	0.02431
E10a	38.80	2,18	-	0.8117
E10c	14.25	2,9	-	0.76
E10e	2.066	6	-0.3492 to 4.134	0.4156

Supplementary Results

The pore size of meso-macroporous hydrogel

Our methodology in this study presents isolating EVs up to ~400 nm, owing to the unprecedented porosity of the cryo-photocrosslinked PEGDA hydrogel; therefore, we clarify that we do not claim any biological importance of EVs smaller or larger than 400 nm. While acknowledging that the most commonly accepted size-based classification of EVs includes small EVs (sEVs) between 30 and 150 nm and larger microvesicles (MVs) between 100 and 1,000 nm, we also recognize that the classification of EVs has still evolved continuously, and the MISEV 2023 recommends avoiding strict size-based classifications.

However, some studies reported that circulating MVs, which are relevant in systemic circulation and body fluids, could possess subpopulations of EVs smaller than 400 nm[1, 2]. Győry *et al.* reported that most microvesicles, isolated from blood plasma or synovial fluid, ranged from 80 to 400 nm[2]. Eitan *et al.* described EVs as nano-sized membranous vesicles ranging from 30 to 400 nm secreted by various cell types into circulation and other bodily fluids[1].

Depending on the end users' applications to investigate biological functions of subpopulations in different sizes, the mean size of isolated EVs, correspondingly the cut-off size, can be controlled by changing the freezing temperature for cryo-photocrosslinking. For instance, we could reduce the effective pore size of our meso-macroporous PEGDA hydrogel, as demonstrated in Fig. 5d, e.

Optimization of hydrogel-based direct EV isolation

We found the following six critical isolation parameters: 1) the volumetric composition of PEG700DA precursor solution for cryo-photocrosslinking, 2) optimal dosages of the meso-macroporous hydrogel particles (i.e., total volumes of hydrogel) per processing volume of biofluid, which represents volumetric throughput, 3) ionic strength during the in-gel capture and washes, 4) in-gel capture time, 5) the number of washes, and 6) off-gel recovery time.

Regarding the volumetric throughput (i.e., the second parameter), we measured the number of off-gel recovered nanoparticles depending on the volume of input plasma per batch. Our experimental data revealed that 40 μL (i.e., one hydrogel particle) possessed the isolation capacity to process up to 300 μL of plasma (i.e., 7.5 μL plasma/μL particle) with a

linear increase (Extended Data Fig. 1b). To cross-confirm this isolation capacity of a meso-macroporous hydrogel particle, we also performed a reverse test varying the volume of hydrogel (i.e., the number of hydrogel particles; 40 μL each) per batch to process 800 μL of plasma. We found that the number of isolated EVs proportionally increased until 120 μL (i.e., ~6.7 μL plasma/μL particle) (Extended Data Fig. 1c). Based on these data, we determined a sufficient dosage of 4 particle/mL plasma (1 particle/250 μL plasma). Since we used 10% [v/v] PEG700DA precursor solution, the liquid phase composition in wet hydrogel particles was approximately 90%. In contrast, dehydrated hydrogel particles did not contain any liquid phase until addition to biofluids. We confirmed indistinguishable performance between the wet and dry states of the meso-macroporous hydrogel particles (Fig. 4a, b, and Supplementary Fig. 7).

We also optimized the ionic strength to 1.5 M (Extended Data Fig. 1d and Supplementary Fig. 4), the in-gel capture time to 1 h (Extended Data Fig. 1e), the number of washes to three iterations (Extended Data Fig. 1f), and the off-gel recovery time to 5 min (Extended Data Fig. 1g).

Intriguingly, a non-monotonic change in isolation efficiency depending on the PEGDA composition (Extended Data Fig. 1a) conceivably originated from a superposition of competition between PEG-positive and -negative factors. Increased PEG (precipitant) was favorable for enhanced EV isolation, while more densely crosslinked PEG networks reduced the probability of EVs' transport into the hydrogel due to decreased porosity and tortuosity (i.e., connections between the pores). To determine a sufficient dosage of hydrogel particles (e.g., 4 particle/mL plasma), we designed two sets of experiments, varying the input plasma per hydrogel volume (40 μL; 1 particle; Extended Data Fig. 1b) and the hydrogel volume per input plasma (800 μL; Extended Data Fig. 1c). Because the presence of the salt played a pivotal role in capturing EVs in hydrogel actively, we explored an optimal ionic strength exhibiting the highest yield and purity (Extended Data Fig. 1d and Supplementary Fig. 4a). In determining the three washes, we prioritized purity toward removing proteins almost completely (Supplementary Fig. 4b) than yield (Extended Data Fig. 1f).

In-depth characterizations of EVs isolated by meso-macroporous hydrogel

In proposing a new methodology, in-depth characterizations of EVs isolated directly by

meso-macroporous hydrogel and comparisons with other established approaches are indispensable. Accordingly, we focused primarily on verifying that off-gel recovered nanoparticles were EVs using various analytical techniques. More specifically, in alignment with the minimal information for studies of extracellular vesicles (MISEV) guidelines[3,4], we employed nanoparticle tracking analysis (NTA), Bradford protein assay, cryo-transmission electron microscopy (cryo-TEM), and western blot. In addition, we conducted comparative analyses not only with ultracentrifugation (UC) but with density gradient ultracentrifugation (DGUC), size-exclusion chromatography (SEC), and precipitation (representatively ExoQuick). For consistency of this verification, we chose the commercially available human plasma as a model biofluid due to accessibility and affordability; plasma has been the most widely studied source of EVs.

Multiplex western blots revealed that the off-gel recovered nanoparticles possessed all three widely consented positive markers of EV-membrane proteins: CD63, CD9, and TSG101 (Extended Data Fig. 3a). In contrast, negative markers (i.e., non-EV-originated protein contaminants), CNX (calnexin) and APOB (apolipoprotein B; also a marker for low-density lipoprotein (LDL)) did not appear (Extended Data Fig. 3a). None of the conventional techniques showed these results except for ultracentrifugation, where bolts' intensities were relatively faint. To simultaneously assess the yield and purity of various isolation methods, we carried out western blots with an identical volume (13 μL) of isolates acquired with the equal volume (0.5 mL) of the recovery buffer, initially from the exact volume of 0.5 mL plasma. Notably, 0.5 mL is the maximum processing volume per batch of DGUC and SEC. Western blots from isolates by DGUC were nearly invisible conceivably because the amount of EVs isolated from 0.5 mL plasma was too small based on NTA (Extended Data Fig. 3a), which also reflects an inherent limitation that it would be extremely challenging to recover measurable EVs without substantial losses during the generation of multiple (four in our experiments) layers of density gradient from the standard maximum capacity of 0.5 mL per batch. We attribute nominally higher yield and purity – by definition, nanoparticle amount from NTA per protein amount – of SEC (Extended Data Fig. 3b, c) to non-EV particles because western blots' intensities for positive markers were faint, like DGUC. Other studies have reported similar observations elsewhere[5,6]. Notably, isolates by precipitation (i.e., ExoQuick) contained the non-EV-originated protein contaminants, much more pronounced for APOB. Size distributions

of nanoparticles isolated by the four existing methodologies were statistically insignificant except that those by DGUC were slightly smaller, probably due to the extraction of a portion in the four density gradient layers (Extended Data Fig. 3d). These results reaffirm that the off-gel recovered nanoparticles were indeed EVs and that our approach exhibited competently higher or comparable isolation efficiency and purity than the existing methods.

Furthermore, we performed proteomics by liquid chromatography-tandem mass spectrometry (LC-MS/MS) and RNA sequencing to examine compositions of off-gel recovered EVs. Firstly, we compared the proteomic profile of the hydrogel-based direct isolation with DGUC, ultracentrifugation, and SEC. By cross-checking all proteins identified with databases of the Vesiclepedia[7] for EVs and the PeptideAtlas[8] for human plasma, we classified the proteins into four categories: 1) EV & plasma, 2) EV only, 3) plasma only, and 4) others. Over 98% of protein abundance, calculated by summed areas of connected peptide peaks, consisted of EV proteins in all four isolation methodologies, predominantly EV & plasma proteins: 98.10% (hydrogel), 99.34% (DGUC), 98.15% (ultracentrifugation), and 98.04% (SEC) (Extended Data Fig. 5a, b). We observed the most significant proportion of overlaps among the hydrogel-based isolation, ultracentrifugation, and SEC: 74 proteins (32.9%) out of 225 proteins categorized in 1) EV & plasma and 2) EV proteins. We found 25 proteins (11.1%) overlapped in all four methods, including DGUC. In comparison, 23 proteins (10.2%) were common in the hydrogel-based isolation and ultracentrifugation (Extended Data Fig. 5c). Notably, we identified 57 EV proteins out of 63 proteins from DGUC, substantially less than the other three methodologies (Extended Data Fig. 5d), which would be due to the limited yield of DGUC that could complicate the profiling by LC-MS/MS. These data suggest that the proteomic compositions originating from EVs are comparable regardless of the isolation methodology, including our hydrogel-based direct isolation with small portions of variations.

Secondly, RNA sequencing of off-gel recovered EVs revealed that plasma EVs carried 18.35% microRNA (miRNA), 18.23% transfer RNA (tRNA), 0.24% small nucleolar RNA (snoRNA), 1.93% small nuclear RNA (snRNA), 3.38% Y RNA, 0.04% vault RNA (vtRNA), and 1.94% PIWI-interacting RNA (piRNA), which are small non-coding RNAs with known and predicted functions[9], 41.29% ribosomal RNA (rRNA), and 7.75% genomic repeats (Extended Data Fig. 6a). By cross-checking our sequencing data with the two databases of Vesiclepedia[7] and EVmiRNA[10] providing miRNA evidence from a perspective of diagnostic potential, we

found 97 overlapping mature miRNAs, including those with top 10 reads, out of 706, accounting for one or more reads (Extended Data Fig. 6b, c and Supplementary Data). Unfortunately, several trials of RNA sequencing on EVs isolated by DGUC led to failures in either passing initial quality control or profiling RNAs. Our tests included pooling six batches to obtain EVs from 3 mL of plasma; total RNAs were 0.01 μg. The inherently low volumetric throughput (i.e., maximum process capacity per batch of 0.5 mL) and complexity of DGUC imposed the limited isolation efficiency in carrying out RNA sequencing despite the high purity of DGUC-isolated EVs[11] (Extended Data Fig. 3b, c); we have not identified studies reporting RNA sequencing of DGUC. Meanwhile, we could perform RNA sequencing with off-gel recovered EVs from single batches with 5 mL of plasma each, which implies superior volumetric throughput of our direct EV isolation with meso-macroporous hydrogel particles.

Regarding the recovery rate of EVs, we used an EV suspension with a known concentration of 2×10^9 particle/mL from human whole blood by ultracentrifugation, as shown in Supplementary Fig. 5. Our hydrogel-based isolation showed recovery rates of 40% to 60% without a change in size distributions compared with the EV suspension (Supplementary Fig. 5b, c, d, g, h). Because the hydrogel-based isolation accompanies no centrifugation, our approach resulted in no size-associated bias in the EV sub-population. Meanwhile, ultracentrifugation's recovery rates ranged from 21% to 27%, with a slight increase in the size of isolated EVs. This shift in the size distribution would be inevitable conceivably due to losses of relatively smaller (lighter) EVs during aspirating supernatants after ultracentrifugation. Notably, our meso-macroporous hydrogel-based isolation has consistently yielded at least twice as much as ultracentrifugation. Similarly, as shown in Extended Data Fig. 1f, we carried out multiple washes during isolation from the EV suspension and estimated corresponding recovery rates. We found that multiple washes reduced the recovery rate from 73% to 24% (Supplementary Fig. 5e) without changes in the size distribution (Supplementary Fig. 5f).

Supplementary Discussion

Distinct characteristics of hydrogel-based direct EV isolation compared with SEC

The EV isolation by conventional size exclusion chromatography (SEC) and the isolation with our cryo-photocrosslinked PEGDA hydrogel particles are distinct in terms of the physicochemical characteristics of the gel materials employed and their fundamental principles.

The Sephacryl resins comprise a crosslinked copolymer of ally dextran and N, N'-methylene bisacrylamide and possess pores that EVs cannot penetrate. Although it is difficult to define the pore sizes of these copolymeric matrices, we could infer the effective pore size ranges from fractionation ranges specified in molecular weights. In the case of Sephacryl S-400, a fractionation range for globular proteins is 20 ~ 8,000 kDa [https://www.cytivalifesciences.com/en/us/shop/chromatography/resins/size-exclusion], corresponding to the calculated hydrodynamic radii [Wu et al., RSC Advances, 2016] from 2 to 15 nm. Therefore, the effective pore size of Sephacryl S-400 would be approximately 30 nm; in other words, only smaller than 30 nm nanoparticles can flow through Sephacryl S-400 beads. Sephacryl S-500 has a more extensive fractionation range of 40 ~ 20,000 kDa for dextran, and its effective pore size would be approximately 40 nm (Stokes diameter of 20,000 kDa dextran). Conversely, EVs can penetrate our cryo-photocrosslinked PEGDA hydrogel with the effective pore size of ~400 nm.

The principle of EV isolation by SEC follows the ***passive sieving*** mechanism. Relatively smaller particles, corresponding to the fractionation range or size exclusion limit, flow through porous beads such as Sephacryl S-400, and particles larger than the fractionation range, including EVs, pass by the porous beads. Accordingly, EVs are eluted relatively quickly, whereas impurities are eluted later. Because the mechanism of SEC is solely size-based, one must harvest a portion of eluate, which contains EVs, at a specific retention time. Moreover, the source of EVs must be pre-processed to eliminate much larger impurities even before SEC, ensuring smooth elution without clogging.

In contrast, the uniqueness of exploiting the cryo-photocrosslinked PEGDA hydrogel matrix lies in the ***active (i.e., reversible but selective) capture*** of EVs by surface charge-selective interactions between EVs and the polymer chains in the gel phase under high ionic

strength. Therefore, this fundamentally disparate principle reflects the following distinct advantageous features unattainable by conventional methodologies, including SEC:

1) The rapidity of EV isolation: reducing isolation time to 15 min with smaller meso-macroporous PEGDA hydrogel particles (10 μL) by adjusting in-gel capture and wash times (Fig. 5c, Extended Data Fig. 8).

2) The custom enrichment of EVs on demand: concentrating isolated EVs up to ~10 times by adjusting the off-gel recovery volume (Fig. 5a, b).

3) The flexibility in EV isolation timing: pausing after the in-gel capture and storing lyophilized hydrogel particles as a solid-phase EV-preserving carrier for up to two months before the off-gel recovery (Fig. 4e).

4) The tunability of meso-macroporosity: isolating subpopulations of EVs by precisely tuning the effective pore size over a few hundred nanometers with smaller ice crystals (i.e., porogen) formed at -195 °C (Fig. 5d, e) or larger ice crystals.

Along with these compelling features, the size-exclusion feature, originating from the unprecedented pore size of our meso-macroporous hydrogel, enables EV isolation without requiring the tedious pre-processing steps essential for all other conventional methods.

The bind-elute SEC (BE-SEC) reported by Corso *et al.* used positively charged hydrophobic octylamine ligands within the core of beads to trap impurities smaller than ~14 nm (700 kDa)[12]. Due to this affinity-based trap, they highlighted enhancing the temporal resolution of eluate, leading to time-efficient isolation by SEC in 85 min while maintaining purity comparable to ultracentrifugation. The BE-SEC hampers primarily non-targeted impurities' elution. In short, the BE-SEC remains SEC, after all. However, because our approach, as stated above, utilized the surface charge-selective interactions between EVs and hydrogel, we could capture EVs (targets) specifically even in 15 min (feature 1 above).

Yang *et al.* presented a single-step approach using superabsorbent polymer (SAP) beads to "concentrate" EVs up to 5-7 times, not "isolate" them[13]. This method requires further isolation steps, such as SEC, to acquire purified EVs, fundamentally different from our methodology. Our methodology emphasizes the isolation of EVs without the need for any pre- or post-treatments while rendering enrichment 10 times (feature 2 above).

Elaboration on recently emerging technologies for EV isolation

Recently published emerging technologies include the utilization of Ca^{2+}-dependent affinity[14], asymmetrical flow field-flow fractionation (AF4)[15], tangential flow filtration (TFF)[16], superabsorbent polymer (SAP)[13], electrochemical fluidic stimulation[17] and double-coupled harmonic oscillation (i.e., EXODUS)[6].

The **Ca^{2+}-dependent affinity** utilizes T-cell membrane protein 4 (TIM4)-coated magnetic beads that bind specifically to the phosphatidylserine displayed on EV surfaces in the presence of Ca^{2+} ions. Therefore, the recovery of EVs is achievable by chelating Ca^{2+} with a chelator (e.g., EDTA). However, this approach merely allows for isolating specific sub-populations of EVs (i.e., phosphatidylserine-displayed EVs)[18].

The **AF4** relies on hydrodynamics in a microfluidic channel. Adjusting cross-flow rates through a membrane on the bottom of the microchannel controls the retention time of particles, leading to time-dependent elution of particles; earlier eluent contains relatively small particles. Because this methodology requires specialized instruments and skilled operators for precise microfluidic controls, its versatile utility would be limited for non-professionals who need to become more familiar with microfluidic techniques. Also, processing throughput would be low without massive parallelization.

The **TFF** employs hydrodynamics-aided dual filtration by sequentially cross-flowing through two membranes with 200 and 30 nm pore sizes. Similarly to the AF4, specialized devices and instruments are required. Despite the advantageous aspect of relatively high permeate rates, undesired clogging could occur during nanofiltration, for instance, when applied to viscous biofluids such as saliva.

The **SAP**, typically in the bead form, absorbs macro- and small molecules, including water, and focuses primarily on acquiring EV-enriched media. Therefore, this approach still requires additional isolation of EVs by other methodologies such as SEC.

The **electrochemical fluidic stimulation** utilizes antibody-coated microfluidic devices to capture EVs by affinity with EV-specific antibodies. Then, altering pH and applying voltage in the microchannel allows for the release of EVs. The critical disadvantage is approximately two orders of magnitude lower yields than the conventional methodology (e.g., UC). Also, this electrochemical stimulation may lead to physicochemical damage to the recovered EVs.

The **double-coupled harmonic oscillation** exploits a specialized instrument and a device integrated with coupled high- and low-frequency oscillators and nanoporous membranes to create membrane vibration by transverse harmonic waves. Upon the membrane vibration, EVs larger than the membrane's pore size reside in a chamber between the two membranes while proteins pass out of the membranes. This emerging technology renders EV isolation time-saving and suitable for a wide range of sample volumes, but the reliance on sophisticated devices and instruments could hinder widely impactful accessibility.

Key advantages of hydrogel-based direct EV isolation

Unlike conventional methods, which require labor-intensive pre-treatment steps, our method offers direct isolation of EVs without complex preprocessing. This feature drastically reduces the time and labor required for the isolation process. Additionally, despite the simplified process, the purity and yield of the isolated EVs remain comparable to or even exceed those obtained through conventional methods, indicating improved efficiency.

Our highly scalable methodology enables the purification of EVs from liter-scale samples (e.g., ascites and milk) in a single batch. This advancement holds promise for transitioning EV research from the laboratory scale to the clinical settings and the industrial scale. As an example from a clinical oncology perspective, ascites-derived EVs have recently gained attention in caring for gastric cancer patients because the ascites EVs played a pivotal role in autocrine signaling and immunomodulation, holding significant promise for diagnostic and therapeutic applications[19, 20]. Our approach will facilitate clinical research on understudied sources such as ascites EVs by overcoming the technical constraint of isolating EVs from liters of ascites. EV sources for therapeutic and cosmeceutical fields (representatively, stem cell culture media and milk) are available in volumes ranging from hundreds of milliliters to liters, requiring numerous iterations of small batches. The scale-up isolation of milk EVs could empower therapeutic utilities bridging industrial translation, for instance, for cell recovery, tissue regeneration, and wound healing. Another direct applicability would be to exploit our hydrogel for isolating large-scale amounts of group O-red blood cell (RBC) EVs as promising RNA drug delivery cargos[21], which may extend to access to autologous or even allogenic utility of immune-associated EVs from platelets[22] and white blood cells (WBCs).

Our meso-macroporous hydrogel is a stand-alone isolation material and is also cost-effective and reusable. This independence from specialized equipment not only increases accessibility to end users under technical or geographical constraints but also enhances practicality across various needs and settings. For instance, end users can choose when and where to recover EVs on demand with lyophilized EV-preserved hydrogel particles that serve as cold chain-free carriers and perform downstream analyses in distant places (e.g., different countries) after prolonged storage periods while guaranteeing long-term shelf life and sustained performance.

In addition to the unique features presented in this study, our approach's highly flexible customizability would make the proceeding of various downstream analyses[23] readily accessible with much more extended utilities. For example, we could explore the porosity tunability further by varying the freezing temperature for a refined investigation of size-specific biological functions[24]. Accordingly, finer size-selective isolation of EV subpopulations would become practically feasible and allow elaborating on the subpopulation-specific roles and functions of EVs' surface-displayed membrane proteins and packaged components inside (e.g., miRNAs and proteins)[24]. By customizing the particle size (e.g., 10 μL), end users can also tune isolation time depending on their purpose and the availability of EV sources.

Under the two distinct but interconnected domains of diagnostics and therapeutics, we demonstrated urinary EV-originated miRNA profiling for liquid biopsy of prostate cancer and the cosmeceutical potential of milk EVs as a skin rejuvenator and antioxidant. These capabilities would allow for more diverse applications, such as temporal genetic profiling of stem cells and their differentiation with EVs in culture media without sacrificing the stem cells and investigations of therapeutic aspects of EVs. Moreover, our technology can be exploited to isolate plant EVs that have recently drawn attention to their therapeutic[25, 26, 27] and protective[28] potential with more efficient and safer delivery.

Supplementary Methods

Nanoparticle tracking analysis (NTA)

EV size distribution and concentration were measured using NTA (NanoSight LM10 and NS300; Malvern) following the manufacturer's protocol. EV-depleted phosphate-buffered saline (dPBS) was prepared by ultracentrifugation at 120,000× g for 16 h and filtration through a 220 nm-membrane filter. Samples were diluted in dPBS (10× dilution for plasma and urine, 1× dilution for saliva and hESC/hiPSC culture media) to a final concentration of 1.0×10^8 to 2.5×10^9 particle/mL. A volume of 400 μL of diluted sample was injected into the instrument with a 1 mL syringe. Imaging settings were manually adjusted (camera level 12; analysis gain 4) according to technical guidelines. Acquired data were recalculated to determine EV concentrations before dilution.

Bradford protein assay for purity assessment

Protein concentrations of EV isolates were determined by the Bradford assay (Protein assay dye reagent; Bio-Rad). EV-recovered solutions (10 μL) were mixed with 500 μL of reagent and transferred into a 96-well microplate (Corning). Following 10 min of room temperature incubation, absorbance at 595 nm was recorded using a microplate reader (Synergy HTX; BioTek).

Western and dot blots

Total proteins from EV isolates were extracted using radio-immunoprecipitation assay (RIPA) buffer (1×; GenDEPOT). Proteins were separated by sodium dodecyl sulfate-polyacrylamide gel electrophoresis (SDS-PAGE) and transferred onto NitroPure Nitrocellulose Transfer Membrane (GenDEPOT). Immunolabeling was performed with the following primary and horseradish peroxidase (HRP)-conjugated secondary antibodies:

- anti-CD63 antibody (ab8219; Abcam; 1:1,000)
- anti-TSG101 antibody (ab125011; Abcam; 1:1,000)
- anti-calnexin antibody (ab22595; Abcam; 1:5,000)
- anti-APOA1 antibody (ab52945; Abcam; 1:1,000)
- anti-APOB antibody (ab139401; Abcam; 1:1,000)
- anti-ALB antibody (ab207327; Abcam)

- anti-GOLGA2 antibody (ab52649; Abcam; 1:1,000)
- anti-CSN1S1 antibody (ab166596; Abcam; 1:2,000)
- goat anti-mouse IgG H&L (ab205719; Abcam)
- goat anti-rabbit IgG H&L (ab205718; Abcam)

Chemiluminescence was developed using SuperSignal™ West Femto Maximum Sensitivity Substrate and Pico PLUS Chemiluminescent Substrate (Thermo Scientific) and imaged with Davinch-Chemi™ (Cas400MF; Davinch-K) or ChemiDoc™ Touch (BioRad). Dot blot immunolabeling and imaging were performed identically to western blotting, excluding electrophoresis and membrane transfer.

For ascites EVs, samples were mixed with 5× SDS sample buffer (GenScript) and boiled at 97 °C for 5 min. Proteins (70 μg per lane) were separated using Bolt™ Bis-Tris Plus Mini Protein Gels (12%; Invitrogen) and transferred to 0.2 μm nitrocellulose membranes (Whatman) for 90 min. Membranes were blocked in 5% skim milk (BD Bioscience) or 5% [w/v] bovine serum albumin (BSA; GenDEPOT) in tris-buffered saline with Tween 20 (TBST; Sigma) for 1 h on a shaker at room temperature, incubated overnight with primary antibodies, washed in TBST, then probed with HRP-conjugated secondary antibodies for 2 h at room temperature. Detection was performed using an electrochemiluminescence detection system (Invitrogen). Primary and secondary antibodies used included:

- anti-CD63 antibody (ab59479; Abcam; 1:1,000)
- anti-PDCD6IP (ALIX) antibody (sc-53540; Santa Cruz; 1:500)
- anti-CDH2 (N-cadherin) antibody (11039-R020; SinoBiological; 1:1,000)
- anti-CLDN1 (claudin-1) antibody (13255s; Cell Signaling; 1:500)
- anti-ANG (angiogenin) antibody (62224s; Cell Signaling; 1:500)
- mouse anti-rabbit IgG HRP (sc-2357; Santa Cruz; 1:1,000)
- mouse-IgGκ BP-HRP (sc-51602; Santa Cruz; 1:1,000)

To verify antibody specificity and detect EV-negative markers, western blots included the following positive controls (Extended Data Fig. 3e):

- Human plasma EVs: supernatant after sequential centrifugations (500× g 10 min, 2,000× g 20 min, 10,000× g 30 min)
- hiPSC culture media EVs: cell lysate[29]

- Bovine milk EVs: supernatant after sequential centrifugations (5,000× g 30 min, 12,000× g 1 h)

EV isolates were prepared using identical starting volumes (0.5 mL) for plasma, hiPSC culture media, and milk to ensure fair comparison. Hydrogel- and ultracentrifugation-based isolates were adjusted to 0.4 mL, while SEC isolates were concentrated from 1.5 mL (a default eluate volume[30]) to 0.4 mL using a 100 kDa MWCO centrifugal filter (Amicon Ultra-2; Merck Millipore).

To prepare hiPSC lysates as a positive control, culture media was removed at ~80% confluency. Cells were rinsed with 2 mL PBS, then dissociated with 1 mL Gentle Cell Dissociation Reagent (GCDR; StemCell Technologies, cat. #100-0485) for 10 min at room temperature. Cells were collected by scraping and centrifugation at 1,000 rpm for 3 min, resuspended in 1 mL RIPA buffer (1× RIPA Cell Lysis Buffer with EDTA; GenDEPOT; cat. # R4100-010) containing 100× protease and phosphatase inhibitor (Halt™ Protease and Phosphatase Inhibitor Cocktail; Thermo Fisher Scientific), vortexed for 10 min, and incubated at 4 °C for 1 h. After centrifugation at 13,000× g for 15 min at 4 °C, supernatant was collected as lysate.

Cryo-transmission electron microscopy (cryo-TEM)

Lacey carbon grids were glow-discharged at 25 mA for 25 s in a sputter coater (Cressington 108; Cressington Scientific Inc.). Samples (5 μL) were applied to grids, blotted for 5 s, and vitrified in liquid ethane (Vitrobot FP5350/60; FEI Company). Imaging was conducted on a scanning transmission microscope (S/TEM; Tecnai™ G2 F20; FEI Company) at an accelerating voltage of 200 kV, available at Advanced Analysis Center of Korea Institute of Science and Technology (KIST). Images were acquired at 14.5k and 25k magnifications under cryogenic conditions using a CMOS camera (RIO 16; GATAN).

For *in situ* imaging of EV-captured hydrogel (Supplementary Fig. 3b, c), plunge freezing, as described above, was followed by freeze substitution in a medium containing 5% [w/v] double-distilled water (ddH_2O), 0.1% [w/v] glutaraldehyde, and 0.2% [w/v] uranyl acetate or osmium tetroxide (OsO_4) in anhydrous acetone. Substitution steps, using a freeze substitution and low temperature embedding system (EM AFS2; Leica), included:

- Hold at -90 °C for 40 h

- Ramp to -50 °C over 20 h (2 °C/h)
- Hold at -50 °C for 12 h
- Ramp to -30 °C over 10 h (2 °C/h)
- Hold at -30 °C for 6 h

Samples were washed three times in pure acetone at -30 °C, infiltrated with Lowicryl HM20 resin for >1 day at -30 °C, and polymerized for >72 h at -30 °C. Ultrathin sections were prepared using an ultramicrotome (MTXL; RMC Products), placed on the TEM grid, and examined on the transmission microscope (Tecnai™ G2 F20).

For correlative light and electron microscopy (CLEM; Supplementary Fig. 3a), grids were prepared as above and immunostained with Alexa 674-conjugated anti-CD63 (ab233056; Abcam). Grids were washed with PBS, submerged in a blocking agent for 1 h, washed three times (5 min each) with wash buffer (PBS containing 0.1% [w/v] BSA-c), incubated with the antibody for 1 h at room temperature, washed with wash buffer, and post-fixed with 0.2% [w/v] glutaraldehyde, followed by final wash with DI water. Imaging was performed with EVOS M7000 (Thermo Fischer Scientific) using a Cy5 light cube, installed at the KIST Bio-imaging correlative analysis platform, followed by TEM (Tecnai™ G2 F20).

Negative-staining TEM

Plasma, urine, and hESC culture media samples were analyzed under three conditions: native biofluid before EV isolation, residual EV isolation medium after in-gel capture before washes (“remains”), and EV isolates. Following negative-staining with uranyl acetate, samples were imaged on a scanning transmission microscope (S/TEM; Tecnai™ G2 F20; FEI Company), available at Advanced Analysis Center of Korea Institute of Science and Technology (KIST).

Scanning electron microscopy (SEM)

Hydrogel particles were lyophilized at each stage (before in-gel capture, before, and after off-gel EV recovery). SEM imaging was performed using a scanning electron microscope (Nova Nano SEM 200; FEI Company), available at Advanced Analysis Center of Korea Institute of Science and Technology (KIST).

Fluorescence imaging of EVs captured in hydrogel *in situ*

Plasma EVs were pre-stained with the Vibrant™ DiO (1 μM final concentration), incubated at 37 °C for 30 min, and purified by centrifugation at 100,000× g for 120 min. Pre-stained EVs were then captured in meso-macroporous hydrogel particles (isolation steps 1-6). Confocal fluorescence images (Supplementary Fig. 3c) were acquired on a confocal microscope (LSM800; Zeiss).

EV isolation by ultracentrifugation

Biofluids were pre-cleared by sequential centrifugations at 500× g for 10 min, 2,000× g for 20 min, and 10,000× g for 30 min using a high-speed centrifuge (1236R; LaboGene), followed by filtration through a 450 nm-membrane filter (Millex). Ultracentrifugation was performed at 100,000× g for 120 min (Type 70 Ti rotor; Beckman Coulter). After removing the supernatant liquid, centrifuge tubes were inverted upside down for 5 min to remove further residual liquid. Then, pellets were resuspended in PBS, matching the recovery buffer volume used for hydrogel-based isolation. All centrifugations were performed at 4 °C.

EV isolation by density gradient ultracentrifugation (DGUC)

DGUC followed an established protocol[31]. Gradient layers were prepared in 0.25 M sucrose, 10 mM Tris-HCl (pH 7.4) with iodixanol concentrations of 5%, 10%, 20%, and 40% [w/v]. Gradients were formed sequentially in a 12 mL ultracentrifugation tube: 3 mL of 40% iodixanol buffer (40% layer) on the bottom, 3 mL of 20% layer, 3 mL of 10% layer, 2.5 mL of 5% layer. Plasma (500 μL) was layered atop the gradient (5% layer) and ultracentrifuged at 100,000× g for 18 h at 4 °C (SW 41 Ti swinging-bucket rotor; 331362; Beckman Coulter). Twelve 1 mL fractions from the top were transferred into microcentrifuge tubes. Fraction 7 was diluted with 11 mL PBS and ultracentrifuged at 100,000× g for 2 h at 4 °C to pellet EVs. Pellets were resuspended in PBS after removal of supernatant and residual liquid as described above.

EV isolation by size-exclusion chromatography (SEC)

Plasma was sequentially centrifuged at 1,500× g for 10 min and 10,000× g for 10 min using a high-speed centrifuge (1236R; LaboGene). The supernatant was loaded onto a column (qEV original Gen 2; Izon Science) pre-equilibrated with 10 mL PBS (200 nm filtered).

The first 3 mL was discarded as void volume, and the subsequent three eluted fractions (500 μL each) were collected.

EV isolation by precipitation (ExoQuick)

Plasma underwent identical pre-treatment as for ultracentrifugation. ExoQuick reagent (126 μL; System Biosciences) was mixed into plasma and incubated at 4 °C for 30 min. Samples were centrifuged at 1,500× g for 30 min at 4 °C, supernatants discarded, and pellets resuspended in PBS.

Proteomic analysis of EV proteins by LC-MS/MS

Two technical replicates were prepared for each isolation method. To EV suspensions, 20% [w/v] SDS, 1 M triethylammonium bicarbonate (TEAB; pH 7.55), 1 M $MgCl_2$, and 100× Halt™ protease and phosphatase inhibitor cocktail (ThermoFisher Scientific) were added to achieve 2% SDS, 50 mM TEAB, 2 mM $MgCl_2$, and 1× inhibitor concentrations. Samples were lysed by probe sonication, centrifuged, and supernatants transferred to fresh tubes. Proteins were reduced and alkylated using dithiothreitol (DTT; ThermoFisher Scientific) and iodoacetamide (IAA; ThermoFisher Scientific), then digested with trypsin using S-trap columns (PROTIFI). Peptides were eluted, vacuum-dried, and reconstituted in 0.1% [v/v] formic acid (FA) in H_2O for liquid chromatography-tandem mass spectrometry (LC-MS/MS).

By adjusting parameters for a mass spectrometer (Q-Exactive HF-X; ThermoFisher Scientific), as reported previously[32], proteomic analysis was performed using a system for high-performance liquid chromatography (HPLC; Ultimate 3000; ThermoFisher Scientific), connected to the mass spectrometer. Peptides were loaded into an EASY-Spray column (50 cm × 75 μm; with an inner diameter of 2 μm) and eluted with linear gradients of 1.6-32% [v/v] acetonitrile (ACN) in 0.1% [v/v] FA over 110 min, and 32-72% ACN over 10 min at 300 nL/min. Mass spectral data were acquired in data-dependent acquisition mode and processed using Proteome Discoverer v2.4 (ThermoFisher Scientific) for identification and label-free quantitation of proteins. Raw data have been deposited in a public repository, the Korea BioData Station (K-BDS; https://kbds.re.kr; data ID KPX10000051) with the support of the Proteome Data Curation Center[33].

Analysis of EV RNAs by RNA sequencing

EVs isolated from plasma with meso-macroporous hydrogel particles were lysed in TRIzol (Invitrogen). Total RNA was extracted and dissolved in 40 μL nuclease-free water, then sequenced by Macrogen. Libraries were prepared using the SMARTer smRNA-Seq Kit (Illumina), reverse-transcribed, and amplified. Sequencing reads were filtered for quality, and reads ≥ 50 bp were analyzed. rRNA reads were excluded. Remaining reads were aligned sequentially to miRbase (v22.1), non-coding RNA databases, and RNAcentral 14.0 to identify miRNAs and other RNA types. Read counts were extracted for miRNAs to determine relative abundance. Raw data have been deposited in a public repository, Zenodo[34].

References

1. Eitan, E., *et al.* Age-Related Changes in Plasma Extracellular Vesicle Characteristics and Internalization by Leukocytes. *Sci. Rep.* **7,** 1342 (2017).

2. György, B., *et al.* Detection and isolation of cell-derived microparticles are compromised by protein complexes resulting from shared biophysical parameters. *Blood* **117,** e39-e48 (2011).

3. Thery, C., *et al.* Minimal information for studies of extracellular vesicles 2018 (MISEV2018): a position statement of the International Society for Extracellular Vesicles and update of the MISEV2014 guidelines. *J. Extracell. Vesicles* **7,** 1535750 (2018).

4. Welsh, J. A., *et al.* Minimal information for studies of extracellular vesicles (MISEV2023): From basic to advanced approaches. *J. Extracell. Vesicles* **13,** e12404 (2024).

5. Brennan, K., *et al.* A comparison of methods for the isolation and separation of extracellular vesicles from protein and lipid particles in human serum. *Sci. Rep.* **10,** 1039 (2020).

6. Chen, Y., *et al.* Exosome detection via the ultrafast-isolation system: EXODUS. *Nat. Methods* **18,** 212-218 (2021).

7. Vesiclepedia. [cited 2023]Available from: http://microvesicles.org/

8. PeptideAtlas. [cited 2023]Available from: https://db.systemsbiology.net/sbeams/cgi/PeptideAtlas/GetProteins?atlas_build_id=559&organism_id=2&redundancy_constraint=4&presence_level_constraint=1&action=QUERY

9. O'Brien, K., Breyne, K., Ughetto, S., Laurent, L. C. & Breakefield, X. O. RNA delivery by extracellular vesicles in mammalian cells and its applications. *Nat. Rev. Mol. Cell. Biol.* **21,** 585-606 (2020).

10. EVmiRNA: the extracelluar vesicles miRNA database. [cited 2023]Available from: http://bioinfo.life.hust.edu.cn/EVmiRNA

11. Onódi, Z., *et al.* Isolation of high-purity extracellular vesicles by the combination of iodixanol density gradient ultracentrifugation and bind-elute chromatography from blood plasma. *Front. Physiol.* **9,** 1479 (2018).

12. Corso, G., *et al.* Reproducible and scalable purification of extracellular vesicles using combined bind-elute and size exclusion chromatography. *Sci. Rep.* **7,** 11561 (2017).

13. Yang, H. C., Ham, Y. M., Kim, J. A. & Rhee, W. J. Single-step equipment-free extracellular vesicle concentration using super absorbent polymer beads. *J. Extracell. Vesicles* **10,** e12074 (2021).

14. Nakai, W., *et al.* A novel affinity-based method for the isolation of highly purified extracellular vesicles. *Sci. Rep.* **6,** 33935 (2016).

15. Zhang, H. & Lyden, D. Asymmetric-flow field-flow fractionation technology for exomere and small extracellular vesicle separation and characterization. *Nat. Protoc.* **14,** 1027-1053 (2019).

16. Kim, K., *et al.* Cyclic tangential flow filtration system for isolation of extracellular vesicles. *APL Bioeng.* **5,** 016103 (2021).

17. Krivitsky, V., *et al.* Ultra-fast And Controlled Capturing, Loading, And Release of Extracellular Vesicles by A Portable Microstructured Electrochemical Fluidic Device. *Adv. Mater.* 2212000 (2023).

18. Matsumoto, A., *et al.* Phosphatidylserine-deficient small extracellular vesicle is a major somatic cell-derived sEV subpopulation in blood. *iScience* **24,** (2021).

19. Menay, F., *et al.* Exosomes isolated from ascites of T-cell lymphoma-bearing mice expressing surface CD24 and HSP-90 induce a tumor-specific immune response. *Front. Immunol.* **8,** 221425 (2017).

20. Cai, J., Gong, L., Li, G., Guo, J., Yi, X. & Wang, Z. Exosomes in ovarian cancer ascites promote epithelial–mesenchymal transition of ovarian cancer cells by delivery of miR-6780b-5p. *Cell Death Dis.* **12,** 210 (2021).

21. Usman, W. M., *et al.* Efficient RNA drug delivery using red blood cell extracellular vesicles. *Nat. Commun.* **9,** 2359 (2018).

22. Dai, Z., *et al.* Platelets and platelet extracellular vesicles in drug delivery therapy: A review of the current status and future prospects. *Front. Pharmacol.* **13,** 1026386 (2022).

23. Shah, R., Patel, T. & Freedman, J. E. Circulating Extracellular Vesicles in Human Disease. *N. Engl. J. Med.* **379,** 958-966 (2018).

24. Willms, E., *et al.* Cells release subpopulations of exosomes with distinct molecular and biological properties. *Sci. Rep.* **6,** (2016).

25. Boccia, E., *et al.* Plant hairy roots for the production of extracellular vesicles with antitumor bioactivity. *Commun. Biol.* **5,** (2022).

26. Corvigno, S., *et al.* Enhanced plant-derived vesicles for nucleotide delivery for cancer therapy. *npj Precis. Oncol.* **8,** (2024).

27. Moon, K., Hur, J., Kim, K. P., Lee, K. W. & Kang, J. Y. Surface-Functionalizable Plant-Derived Extracellular Vesicles for Targeted Drug Delivery Carrier Using Grapefruit. *Adv. Mater. Interfaces* **10,** (2023).

28. Weiberg, A., *et al.* Fungal Small RNAs Suppress Plant Immunity by Hijacking Host RNA Interference Pathways. *Science* **342,** 118-123 (2013).

29. Théry, C., Amigorena, S., Raposo, G. & Clayton, A. Isolation and characterization of exosomes from cell culture supernatants and biological fluids. *Curr. Protoc. Cell Biol.* **30,** 3.22.21-23.22.29 (2006).

30. Boing, A. N., van der Pol, E., Grootemaat, A. E., Coumans, F. A., Sturk, A. & Nieuwland, R. Single-step isolation of extracellular vesicles by size-exclusion chromatography. *J. Extracell. Vesicles* **3,** (2014).

31. Lobb, R. J., *et al.* Optimized exosome isolation protocol for cell culture supernatant and human plasma. *J. Extracell. Vesicles* **4,** 27031 (2015).

32. Park, N., *et al.* One-STAGE Tip Method for TMT-Based Proteomic Analysis of a Minimal Amount of Cells. *ACS Omega* (2023).

33. Lee C., Lee H. K. Extracellular vesicle extracted from human plasma by four isolation methods: hydrogel-based, ultracentrifugation, size-exclusion chromatography, density gradient ultracentrifugation. K-BDS. Zenodo. 10.5281/zenodo.15794986 (2024).

34. Macrogen. Raw dataset to analyze RNAs present in human plasma extracellular vesicles isolated with hydrogel particles. Zenodo. 10.5281/zenodo.15796138 (2025).
