# Supplementary material for: Meso–macroporous hydrogel for direct litre-scale isolation of extracellular vesicles
Source: Nat Nanotechnol. 2025 Sep 24;20(11):1678–87. doi: 10.1038/s41565-025-02011-1 (PMC12623240; doi:10.1038/s41565-025-02011-1)
Supplement: Supplementary file 2 — Reporting Summary [file 41565_2025_2011_MOESM2_ESM.pdf]

nature portfolio

Corresponding author(s): Ji Yoon Kang, Ki Wan Bong, Nakwon Choi

Last updated by author(s): May 1, 2025

Reporting Summary

Nature Portfolio wishes to improve the reproducibility of the work that we publish. This form provides structure for consistency and transparency in reporting. For further information on Nature Portfolio policies, see our Editorial Policies and the Editorial Policy Checklist.

Statistics

For all statistical analyses, confirm that the following items are present in the figure legend, table legend, main text, or Methods section.

n/a	Confirmed	
☐	☒	The exact sample size (*n*) for each experimental group/condition, given as a discrete number and unit of measurement
☐	☒	A statement on whether measurements were taken from distinct samples or whether the same sample was measured repeatedly
☐	☒	The statistical test(s) used AND whether they are one- or two-sided<br>*Only common tests should be described solely by name; describe more complex techniques in the Methods section.*
☒	☐	A description of all covariates tested
☐	☒	A description of any assumptions or corrections, such as tests of normality and adjustment for multiple comparisons
☐	☒	A full description of the statistical parameters including central tendency (e.g. means) or other basic estimates (e.g. regression coefficient) AND variation (e.g. standard deviation) or associated estimates of uncertainty (e.g. confidence intervals)
☐	☒	For null hypothesis testing, the test statistic (e.g. F, t, r) with confidence intervals, effect sizes, degrees of freedom and P value noted<br>*Give P values as exact values whenever suitable.*
☒	☐	For Bayesian analysis, information on the choice of priors and Markov chain Monte Carlo settings
☒	☐	For hierarchical and complex designs, identification of the appropriate level for tests and full reporting of outcomes
☒	☐	Estimates of effect sizes (e.g. Cohen's *d*, Pearson's *r*), indicating how they were calculated

Our web collection on statistics for biologists contains articles on many of the points above.

Software and code

Policy information about availability of computer code

Data collection	We did not use any previously unreported custom computer code, algorithm, or software to collect the data reported in this study.
Data analysis	We did not use any previously unreported custom computer code, algorithm, or software to analyze the data reported in this study. We used a custom MATLAB (ver. r2021a) code, previously reported in Biosensors and Bioelectronics (2021), to analyze fluorescence images of PEGDA hydrogel microparticles.

For manuscripts utilizing custom algorithms or software that are central to the research but not yet described in published literature, software must be made available to editors and reviewers. We strongly encourage code deposition in a community repository (e.g. GitHub). See the Nature Portfolio guidelines for submitting code & software for further information.

Data

Policy information about availability of data

All manuscripts must include a data availability statement. This statement should provide the following information, where applicable:

- Accession codes, unique identifiers, or web links for publicly available datasets
- A description of any restrictions on data availability
- For clinical datasets or third party data, please ensure that the statement adheres to our policy

The data supporting this study's findings are available from the manuscript, its supplementary information, or the corresponding authors upon request. The proteomic dataset for EV proteins in human plasma analyzed by chromatography-tandem mass spectrometry (LC-MS/MS) is available at Korea Biodata Station (K-

BDS), a public repository ref. 42]. The dataset for EV RNAs in human plasma analyzed by RNA sequencing is available at Zenodo, a public repository [ref. 43].
42. Lee C., Lee H. K. Extracellular vesicle extracted from human plasma by four isolation methods: hydrogel-based, ultracentrifugation, size-exclusion chromatography, density gradient ultracentrifugation. K-BDS. Zenodo. 10.5281/zenodo.15794986 (2024).
43. Macrogen. Raw dataset to analyze RNAs present in human plasma extracellular vesicles isolated with hydrogel particles. Zenodo. 10.5281/zenodo.15796138 (2025).

Research involving human participants, their data, or biological material

Policy information about studies with human participants or human data. See also policy information about sex, gender (identity/presentation), and sexual orientation and race, ethnicity and racism.

Reporting on sex and gender	This study does not focus on clinical studies (e.g., clinical trials) related to sex and gender. However, we used human clinical urine samples acquired from male participants who consented to detecting urinary EV-originated miRNAs associated with prostate cancer. We also used human clinical ascites samples acquired from two female and three male gastric cancer patients for the isolation of ascites EVs.
Reporting on race, ethnicity, or other socially relevant groupings	We did not use socially constructed or socially relevant categorization variables for this study.
Population characteristics	All the male subjects underwent transrectal ultrasound-guided prostate biopsies and were classified as prostate cancer patients or healthy controls after pathologic examination of specimens. We excluded patients with urothelial carcinoma or other malignant diseases from the patient group. All the five gastric cancer patients' ages ranged from 50 to 73.
Recruitment	All male subjects were referred to a urologist for cancer screening due to an elevated prostate-specific antigen (PSA) level, abnormal findings on digital rectal examination (DRE), or hypoechoic lesions in the prostate gland through transrectal ultrasonography. The five gastric cancer patients consented to provide biospecimens based on approval by the Institutional Review Board (IRB) of the Samsung Medical Center (SMC).
Ethics oversight	Korea University Anam Hospital (IRB approval no. 2017AN0036) for urine samples, Samsung Medical Center (SMC) (IRB#2021-09-052) for gastric cancer patients' ascites.

Note that full information on the approval of the study protocol must also be provided in the manuscript.

Field-specific reporting

Please select the one below that is the best fit for your research. If you are not sure, read the appropriate sections before making your selection.

☒ Life sciences ☐ Behavioural & social sciences ☐ Ecological, evolutionary & environmental sciences

For a reference copy of the document with all sections, see nature.com/documents/nr-reporting-summary-flat.pdf

Life sciences study design

All studies must disclose on these points even when the disclosure is negative.

Sample size	We determined the sample sizes based on previous studies cited in this study, which satisfies the requirement for statistical analyses to discuss the experimental data's degree of differences and variability.
Data exclusions	We did not exclude data from the analyses.
Replication	We could verify the reproducibility of the experimental data consistently through multiple experiments and specified technical and biological replicates in the figure captions.
Randomization	We assigned all samples to a randomized experimental group.
Blinding	Due to the proof-of-concept nature of this study, we could not be genuinely blinded to group allocation. However, we conducted data collection and analyses for all the experiments in a strictly identical way.

Reporting for specific materials, systems and methods

We require information from authors about some types of materials, experimental systems and methods used in many studies. Here, indicate whether each material, system or method listed is relevant to your study. If you are not sure if a list item applies to your research, read the appropriate section before selecting a response.

Materials & experimental systems

n/a	Involved in the study	
☐	☒	Antibodies
☐	☒	Eukaryotic cell lines
☒	☐	Palaeontology and archaeology
☒	☐	Animals and other organisms
☒	☐	Clinical data
☒	☐	Dual use research of concern
☒	☐	Plants

Methods

n/a	Involved in the study	
☒	☐	ChIP-seq
☒	☐	Flow cytometry
☒	☐	MRI-based neuroimaging

Antibodies

Antibodies used

We used antibodies to perform western blots: anti-CD63 antibody (ab8219, ab59479; Abcam), anti-TSG101 antibody (ab125011; Abcam), anti-CNX antibody (ab22595; Abcam), anti-APOA1 antibody (ab52945; Abcam), anti-APOB antibody (ab139401; Abcam), anti-GOLGA2 antibody (ab52649; Abcam), anti-CSN1S1 antibody (ab166596; Abcam), anti-PDCD6IP antibody (sc-53540; Santa Cruz), anti-CDH2 antibody (11039-R020; SinoBiological), anti-CLDN1 antibody (13255s; Cell Signaling), anti-ANG antibody (62224s; Cell Signaling), anti-GSH antibody (ab19534; Abcam), anti-GAPDH antibody (ab9485; Abcam), goat anti-mouse IgG H&L (ab205719; Abcam), goat anti-rabbit IgG H&L (ab205718; Abcam), mouse anti-rabbit IgG HRP (sc-2357; Santa Cruz), mouse-IgGκ BP-HRP (sc-51602; Santa Cruz), goat anti-mouse IgG (HRP) (1706516; Bio-Rad), and goat anti-rabbit IgG (HRP) (1706515; Bio-Rad). We used anti-CD63 antibody conjugated with Alexa 647 (ab233056; Abcam) to perform correlative light and electron microscopy (CLEM). We used anti-collagen II primary antibody (ab34712; Abcam) and a secondary antibody (ab205718; Abcam) for immunostaining human dermal fibroblasts.

Validation

The manufacturer's website provides validation of the antibodies used in this study.

Eukaryotic cell lines

Policy information about cell lines and Sex and Gender in Research

Cell line source(s)

Human Embryonic Stem Cell (H1ESC; male; WA01; WiCell), human induced pluripotent stem cell (hiPSC; male; GM25256; Coriell), human dermal fibroblast (hDF; PCS-201-012; ATCC), human keratinocyte cells (HaCaT cells; CRL-2404; ATCC)

Authentication

Human pluripotent stem cell lines (H1 lines) were authenticated in their lab of origin through the expression of pluripotency-associated markers. These cell lines are registered on the NIH Human Embryonic Stem Cell Registry (NIHhESC-10-0043). The GM25256 hiPSC line was authenticated by the Coriell Institute through standard characterization procedures, including karyotyping, surface antigen expression analysis, and alkaline phosphatase activity. Pluripotency was confirmed by embryoid body (EB) formation and real-time PCR analysis of pluripotency-associated gene expression. All characterization data are included in the Certificate of Analysis provided by Coriell.

Mycoplasma contamination

All cell lines used in this study were confirmed to be free of mycoplasma contamination by routine PCR-based testing.

Commonly misidentified lines
(See ICLAC register)

The cell lines used in this study are not listed in the ICLAC Database of Cross-contaminated or Misidentified Cell Lines.

Plants

Seed stocks

We did not use any plant materials.

Novel plant genotypes

Describe the methods by which all novel plant genotypes were produced. This includes those generated by transgenic approaches, gene editing, chemical/radiation-based mutagenesis and hybridization. For transgenic lines, describe the transformation method, the number of independent lines analyzed and the generation upon which experiments were performed. For gene-edited lines, describe the editor used, the endogenous sequence targeted for editing, the targeting guide RNA sequence (if applicable) and how the editor was applied.

Authentication

Describe any authentication procedures for each seed stock used or novel genotype generated. Describe any experiments used to assess the effect of a mutation and, where applicable, how potential secondary effects (e.g. second site T-DNA insertions, mosiacism, off-target gene editing) were examined.
