# Supplementary figures and images for: Meso–macroporous hydrogel for direct litre-scale isolation of extracellular vesicles
Source: Nat Nanotechnol. 2025 Sep 24;20(11):1678–87. doi: 10.1038/s41565-025-02011-1 (PMC12623240; doi:10.1038/s41565-025-02011-1)

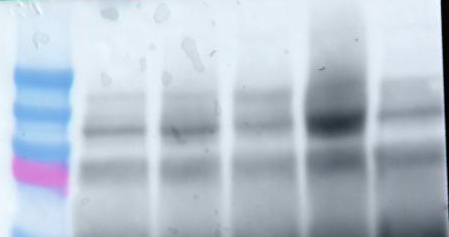

Supplement: Supplementary file 5 — Unprocessed western blots. [file 41565_2025_2011_MOESM5_ESM.zip › Source Data Fig 3b/Fig 3b_Alix.jpg]

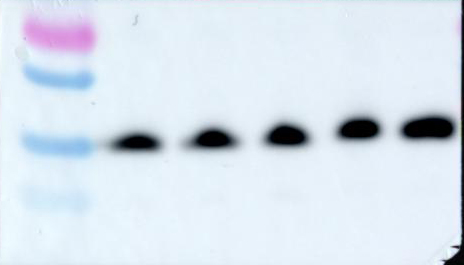

Supplement: Supplementary file 5 — Unprocessed western blots. [file 41565_2025_2011_MOESM5_ESM.zip › Source Data Fig 3b/Fig 3b_Angiogenin.jpg]

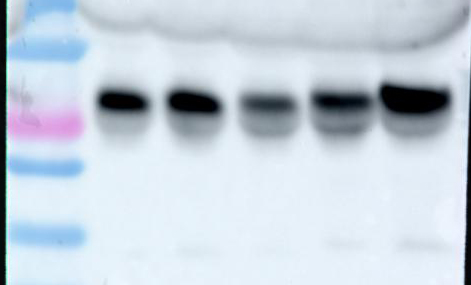

Supplement: Supplementary file 5 — Unprocessed western blots. [file 41565_2025_2011_MOESM5_ESM.zip › Source Data Fig 3b/Fig 3b_CD63.jpg]

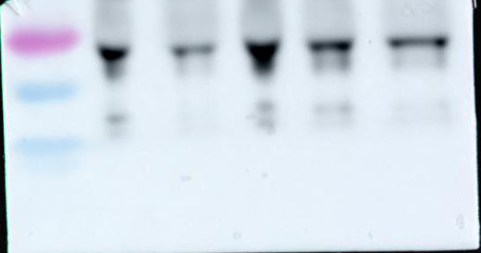

Supplement: Supplementary file 5 — Unprocessed western blots. [file 41565_2025_2011_MOESM5_ESM.zip › Source Data Fig 3b/Fig 3b_Claudin1.jpg]

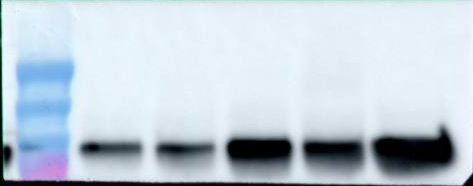

Supplement: Supplementary file 5 — Unprocessed western blots. [file 41565_2025_2011_MOESM5_ESM.zip › Source Data Fig 3b/Fig 3b_N-cadherin.jpg]

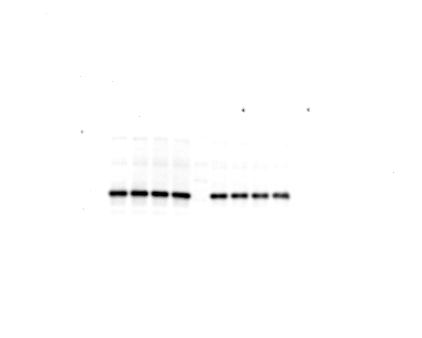

Supplement: Supplementary file 9 — Unprocessed western blots. [file 41565_2025_2011_MOESM9_ESM.zip › Source Data Fig 6g/Fig 6g_GAPDH_Chemiluminescence.tif]

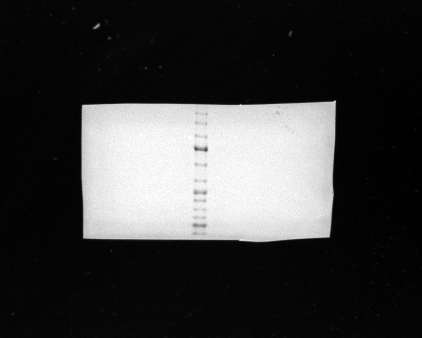

Supplement: Supplementary file 9 — Unprocessed western blots. [file 41565_2025_2011_MOESM9_ESM.zip › Source Data Fig 6g/Fig 6g_GAPDH_Colorimetric.tif]

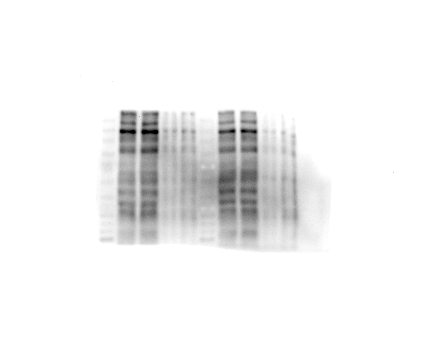

Supplement: Supplementary file 9 — Unprocessed western blots. [file 41565_2025_2011_MOESM9_ESM.zip › Source Data Fig 6g/Fig 6g_GSH_Chemiluminescence.tif]

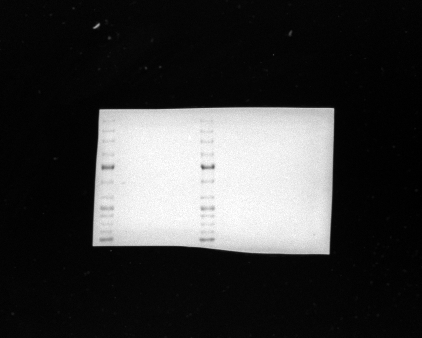

Supplement: Supplementary file 9 — Unprocessed western blots. [file 41565_2025_2011_MOESM9_ESM.zip › Source Data Fig 6g/Fig 6g_GSH_Colorimetric.tif]

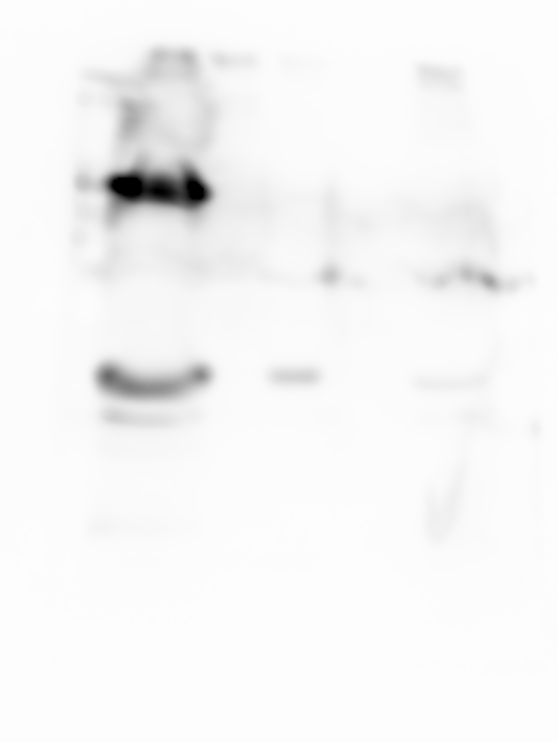

Supplement: Supplementary file 13 — Unprocessed western blots. [file 41565_2025_2011_MOESM13_ESM.zip › Source Data Extended Data Fig 3a_3e/Fig E3a_APOB_Chemiluminescence.tif]

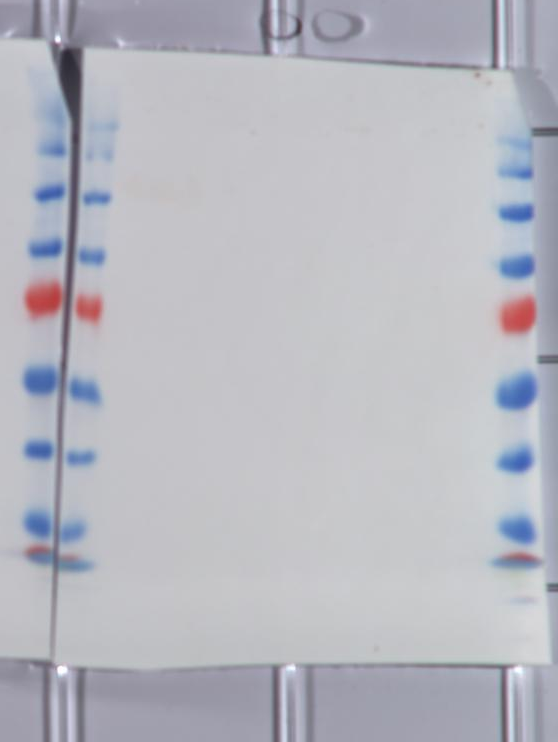

Supplement: Supplementary file 13 — Unprocessed western blots. [file 41565_2025_2011_MOESM13_ESM.zip › Source Data Extended Data Fig 3a_3e/Fig E3a_APOB_Colorimetric.tif]

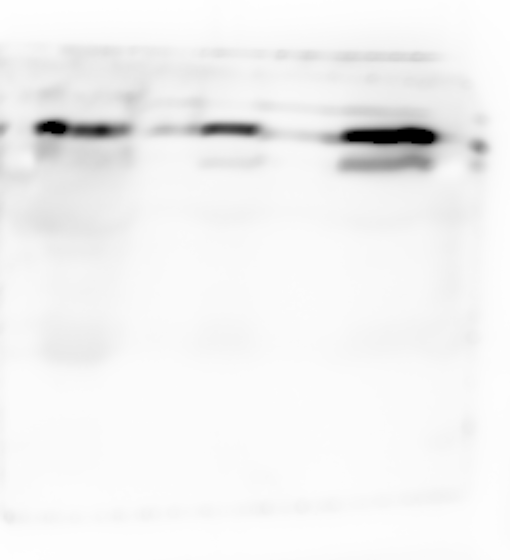

Supplement: Supplementary file 13 — Unprocessed western blots. [file 41565_2025_2011_MOESM13_ESM.zip › Source Data Extended Data Fig 3a_3e/Fig E3a_CD63_Chemiluminescence.tif]

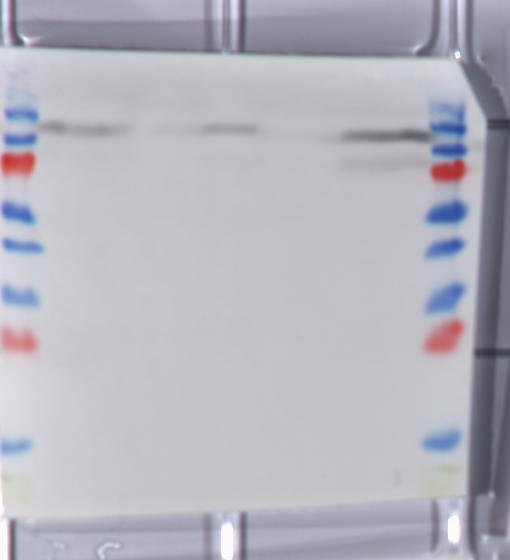

Supplement: Supplementary file 13 — Unprocessed western blots. [file 41565_2025_2011_MOESM13_ESM.zip › Source Data Extended Data Fig 3a_3e/Fig E3a_CD63_Colorimetric.tif]

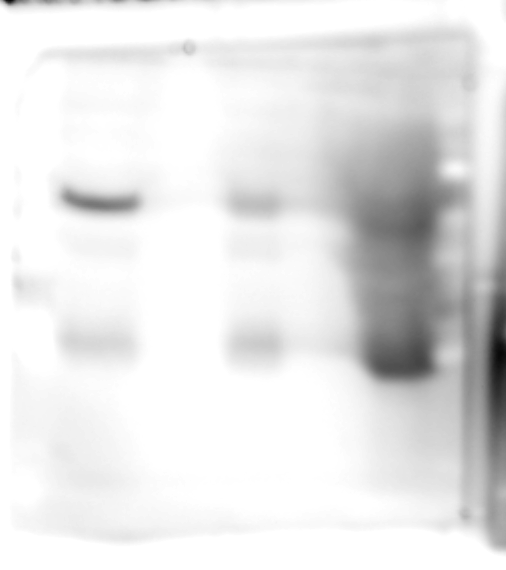

Supplement: Supplementary file 13 — Unprocessed western blots. [file 41565_2025_2011_MOESM13_ESM.zip › Source Data Extended Data Fig 3a_3e/Fig E3a_CD9_Chemiluminescence.tif]

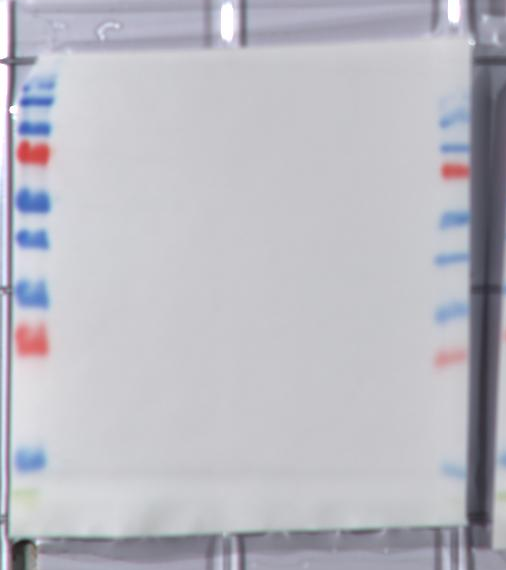

Supplement: Supplementary file 13 — Unprocessed western blots. [file 41565_2025_2011_MOESM13_ESM.zip › Source Data Extended Data Fig 3a_3e/Fig E3a_CD9_Colorimetric.tif]

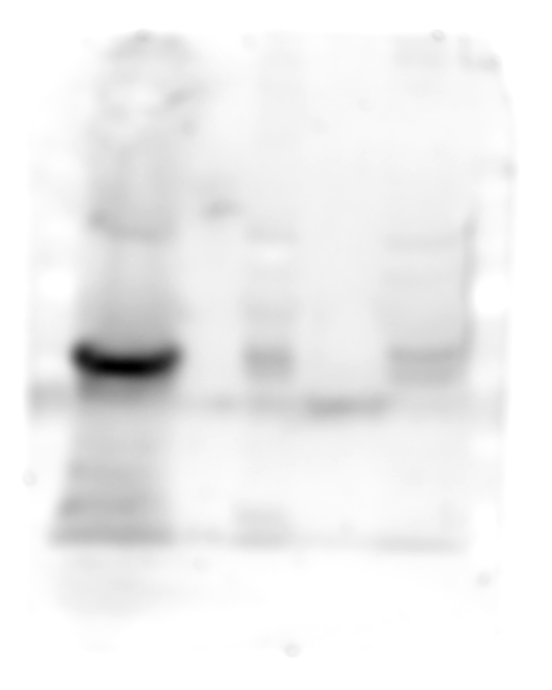

Supplement: Supplementary file 13 — Unprocessed western blots. [file 41565_2025_2011_MOESM13_ESM.zip › Source Data Extended Data Fig 3a_3e/Fig E3a_CNX_Chemiluminescence.tif]

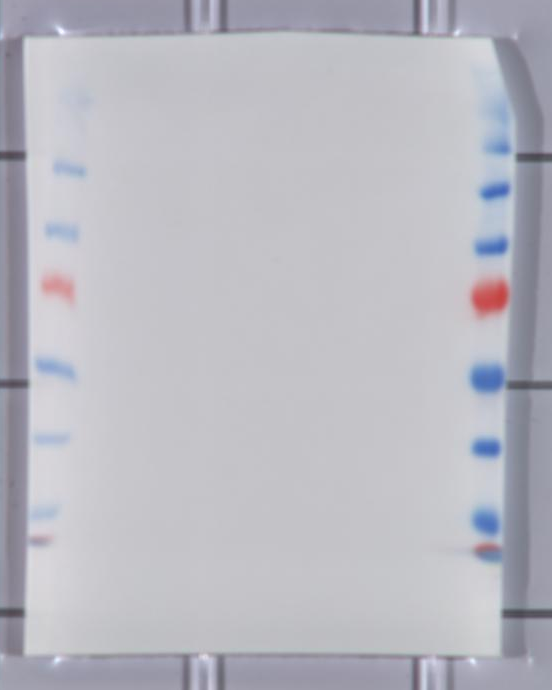

Supplement: Supplementary file 13 — Unprocessed western blots. [file 41565_2025_2011_MOESM13_ESM.zip › Source Data Extended Data Fig 3a_3e/Fig E3a_CNX_Colorimetric.tif]

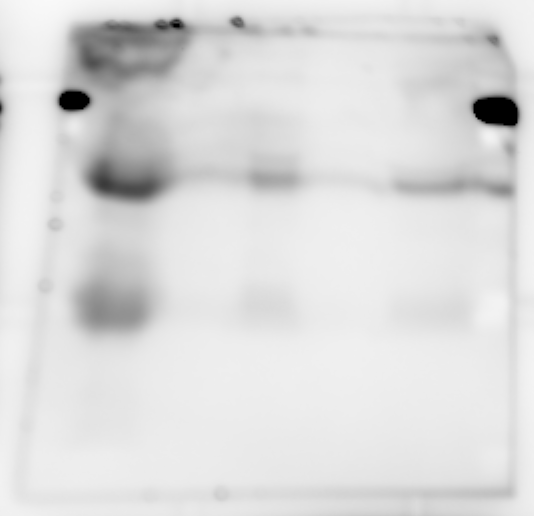

Supplement: Supplementary file 13 — Unprocessed western blots. [file 41565_2025_2011_MOESM13_ESM.zip › Source Data Extended Data Fig 3a_3e/Fig E3a_TSG101_Chemiluminescence.tif]

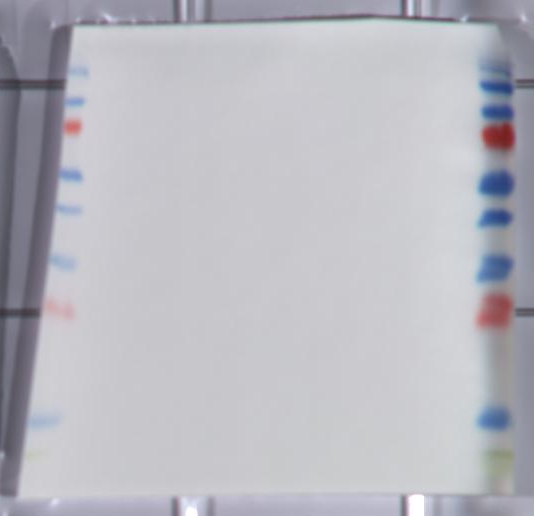

Supplement: Supplementary file 13 — Unprocessed western blots. [file 41565_2025_2011_MOESM13_ESM.zip › Source Data Extended Data Fig 3a_3e/Fig E3a_TSG101_Colorimetric.tif]

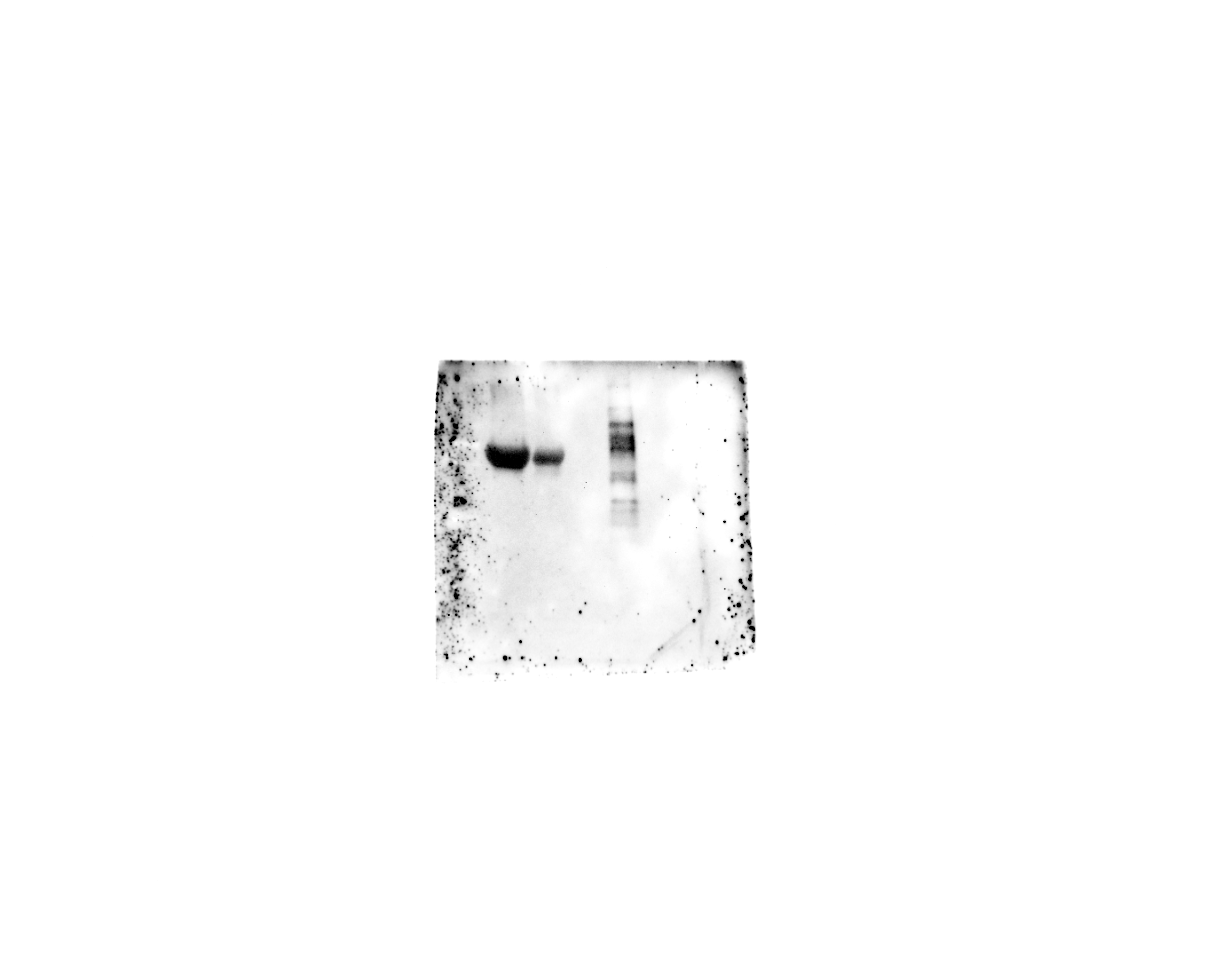

Supplement: Supplementary file 13 — Unprocessed western blots. [file 41565_2025_2011_MOESM13_ESM.zip › Source Data Extended Data Fig 3a_3e/Fig E3e_hiPSC_CD63_Chemiluminescence.jpg]

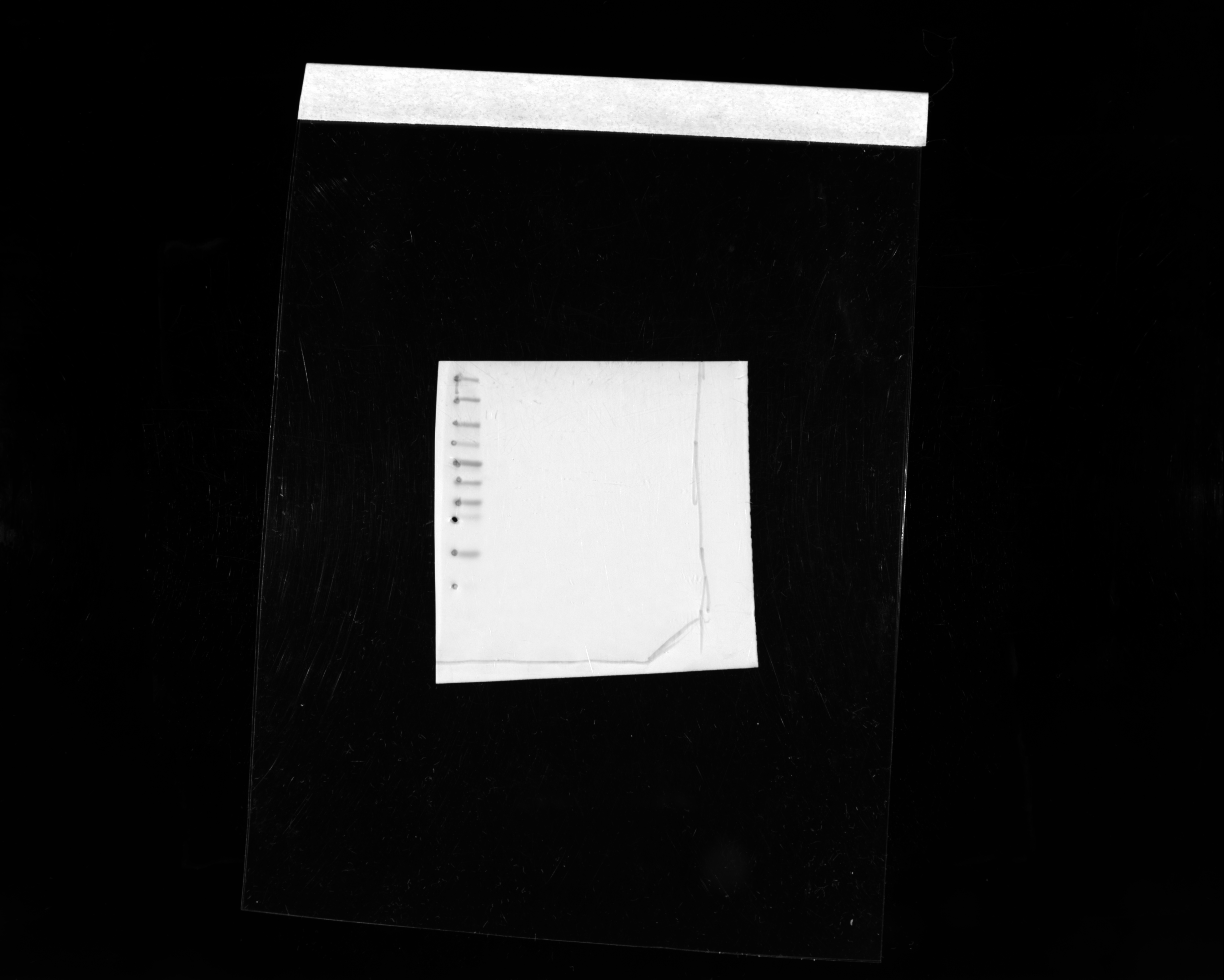

Supplement: Supplementary file 13 — Unprocessed western blots. [file 41565_2025_2011_MOESM13_ESM.zip › Source Data Extended Data Fig 3a_3e/Fig E3e_hiPSC_CD63_Colorimetric.jpg]

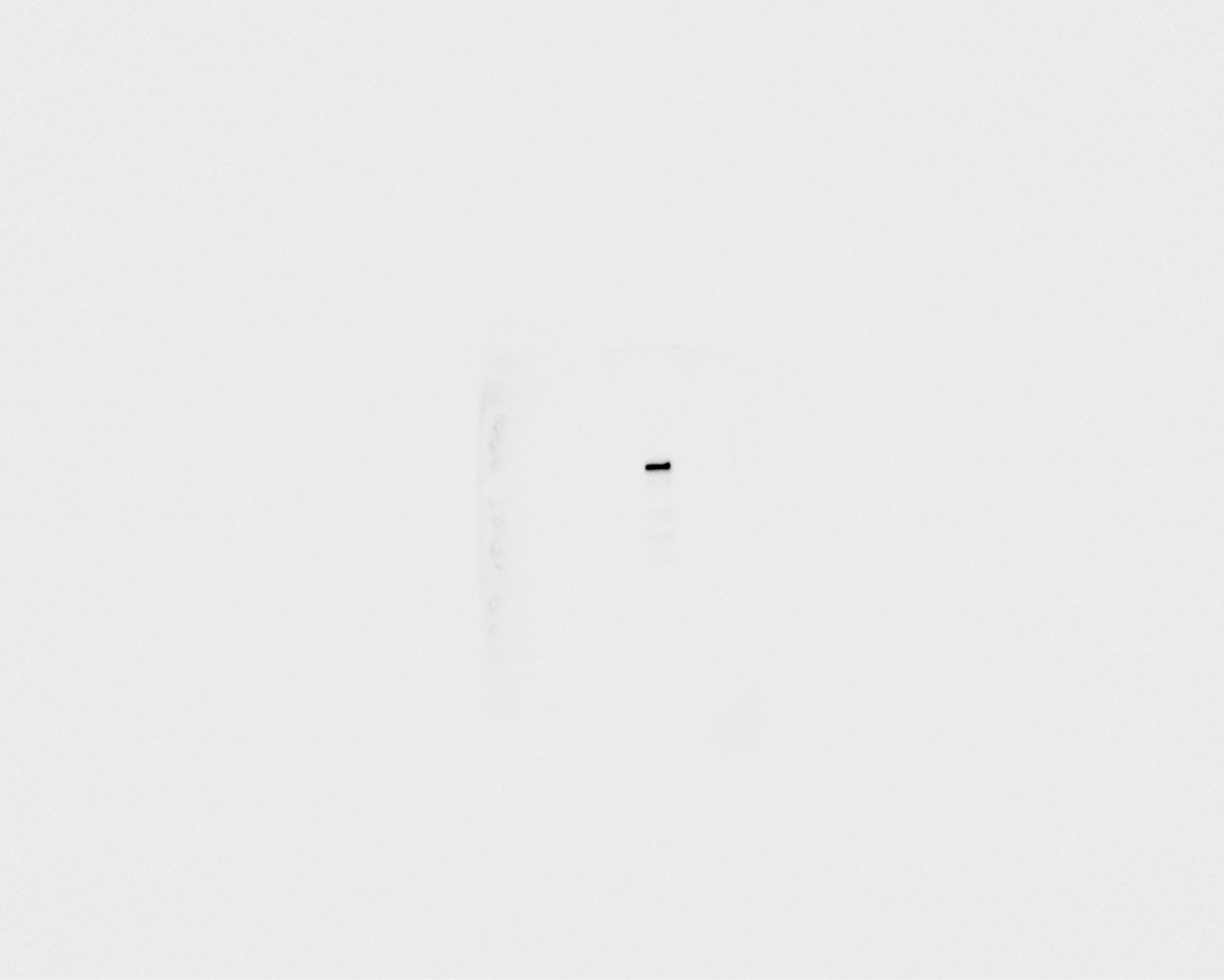

Supplement: Supplementary file 13 — Unprocessed western blots. [file 41565_2025_2011_MOESM13_ESM.zip › Source Data Extended Data Fig 3a_3e/Fig E3e_hiPSC_CNX_Chemiluminescence.jpg]

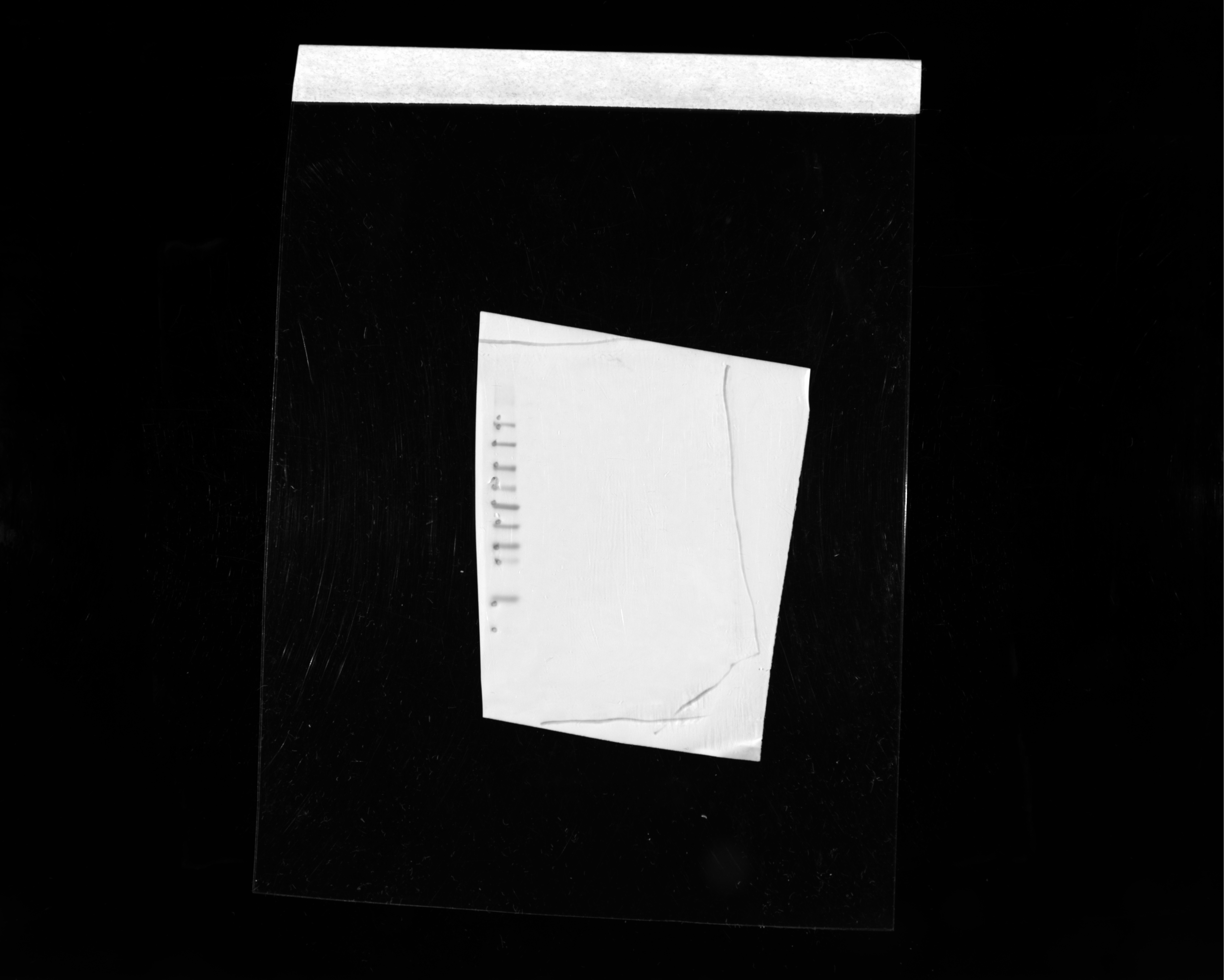

Supplement: Supplementary file 13 — Unprocessed western blots. [file 41565_2025_2011_MOESM13_ESM.zip › Source Data Extended Data Fig 3a_3e/Fig E3e_hiPSC_CNX_Colorimetric.jpg]

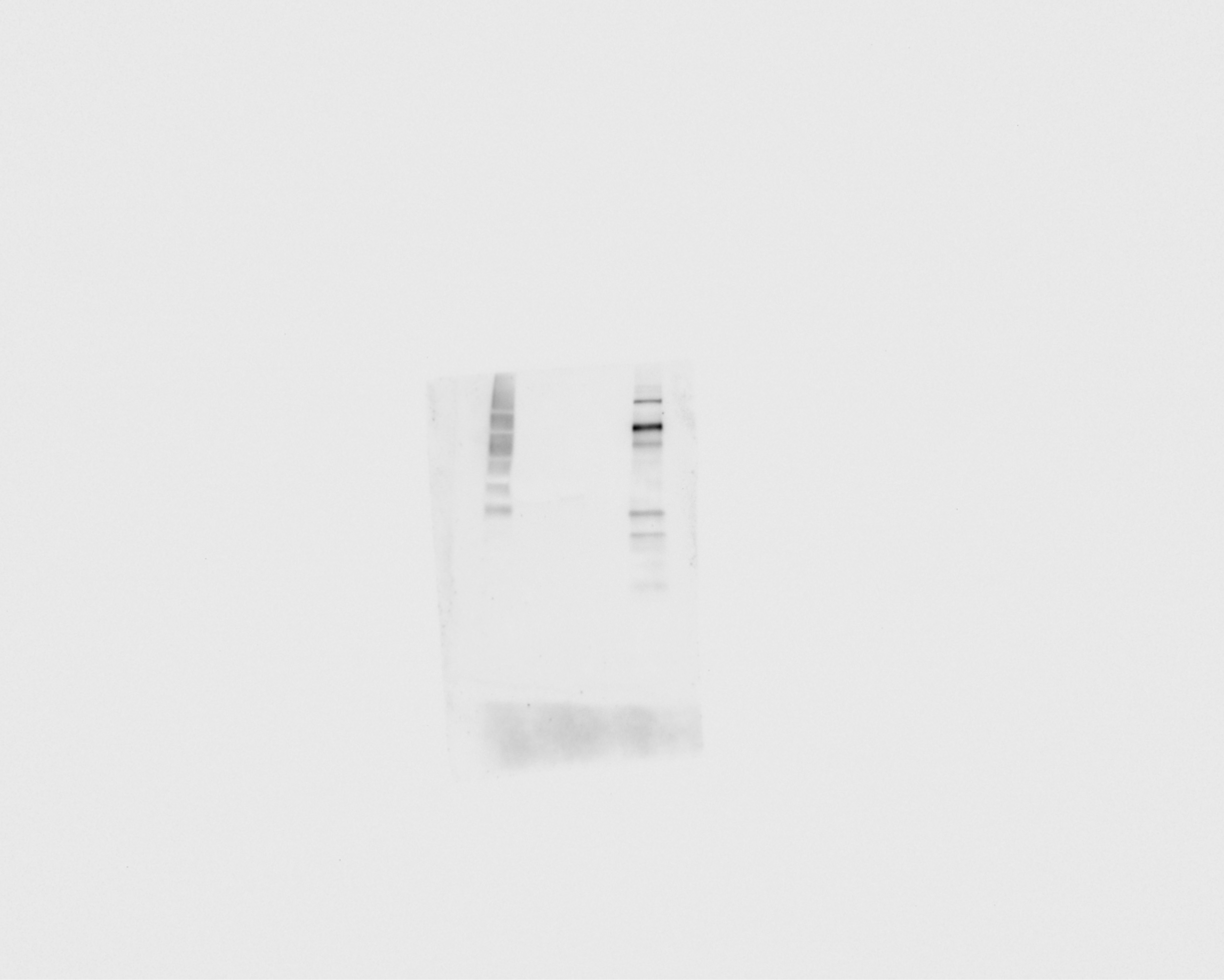

Supplement: Supplementary file 13 — Unprocessed western blots. [file 41565_2025_2011_MOESM13_ESM.zip › Source Data Extended Data Fig 3a_3e/Fig E3e_hiPSC_GOLGA2_Chemiluminescence.jpg]

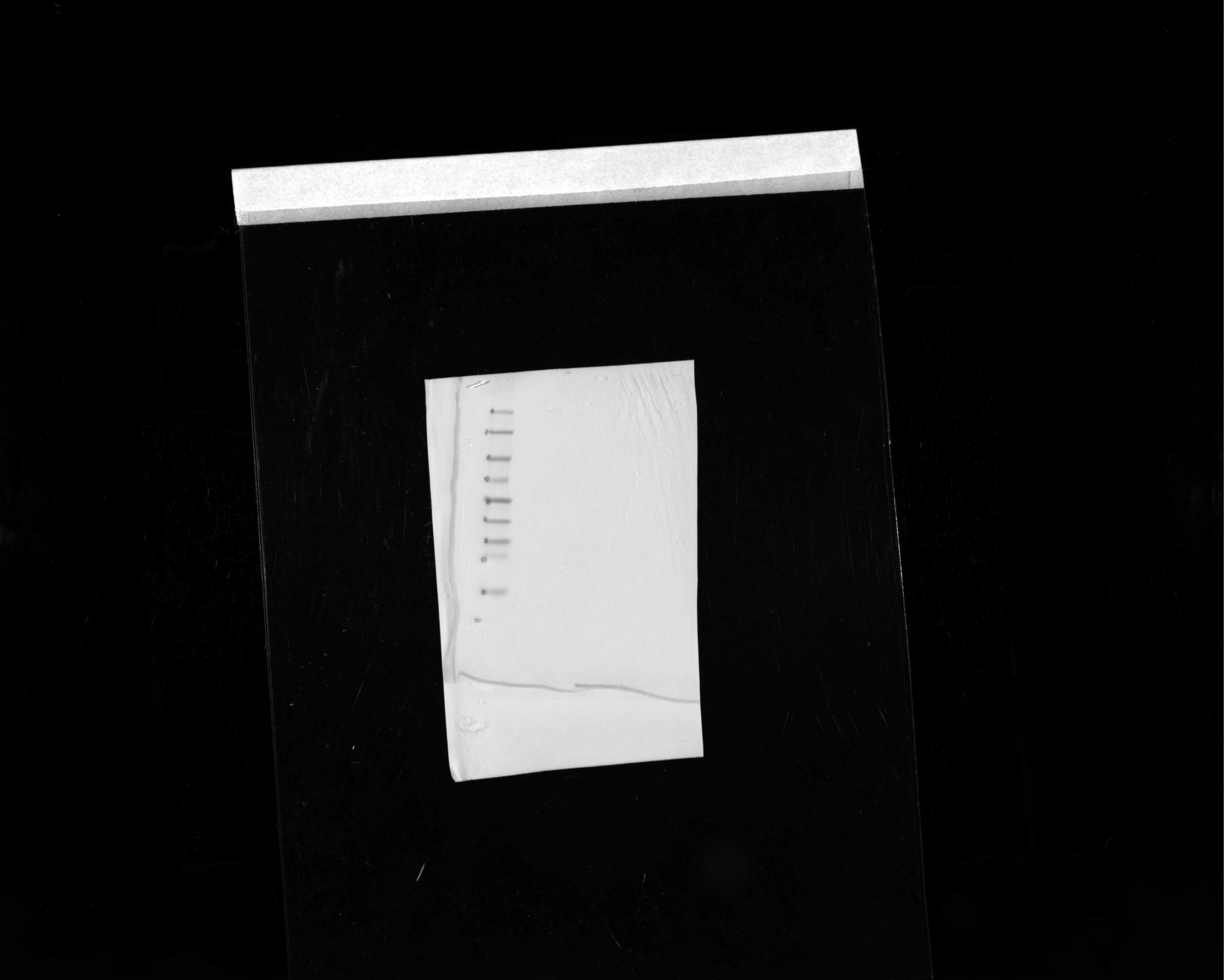

Supplement: Supplementary file 13 — Unprocessed western blots. [file 41565_2025_2011_MOESM13_ESM.zip › Source Data Extended Data Fig 3a_3e/Fig E3e_hiPSC_GOLGA2_Colorimetric.jpg]

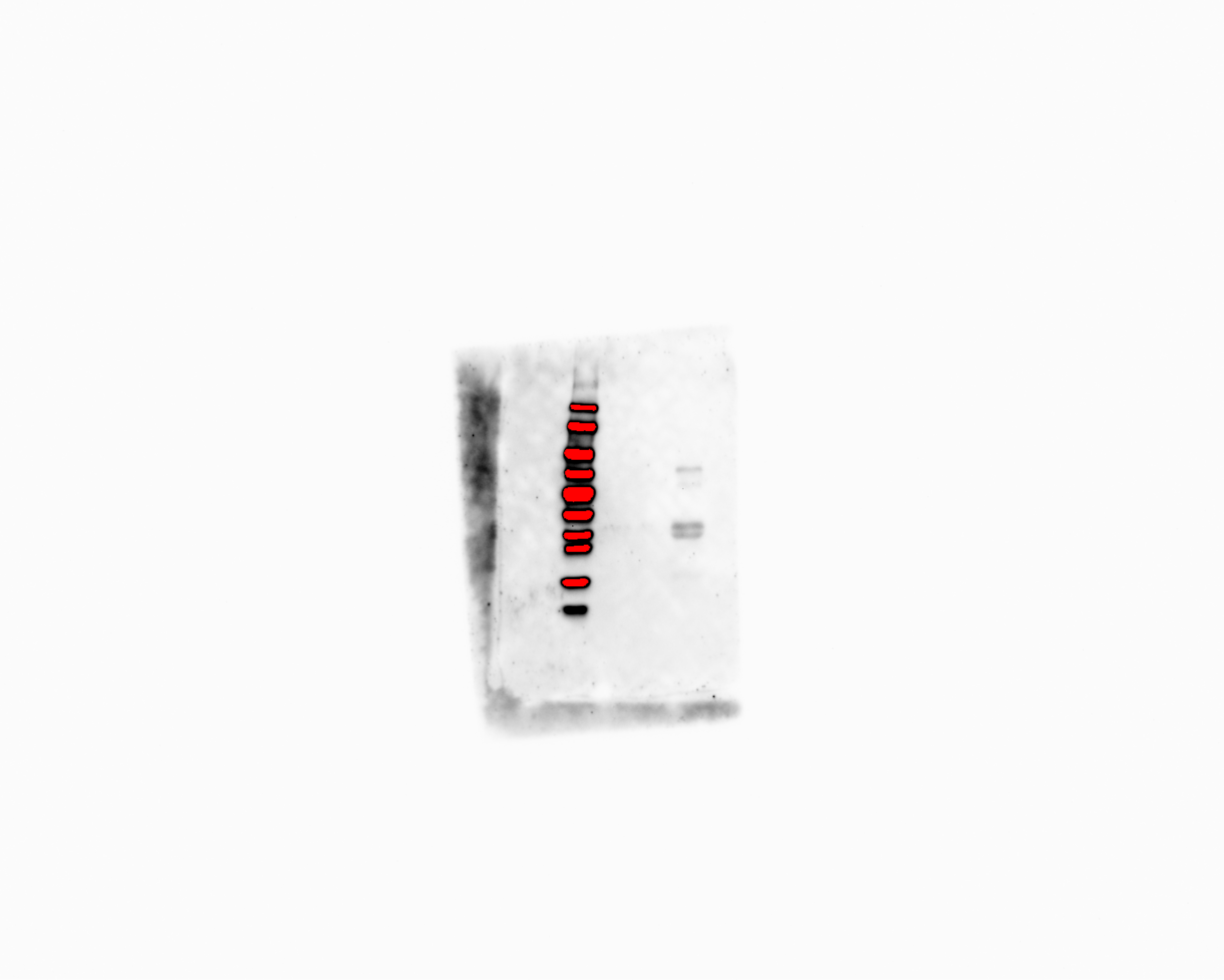

Supplement: Supplementary file 13 — Unprocessed western blots. [file 41565_2025_2011_MOESM13_ESM.zip › Source Data Extended Data Fig 3a_3e/Fig E3e_milk_CSN1S1_Chemiluminescence.jpg]

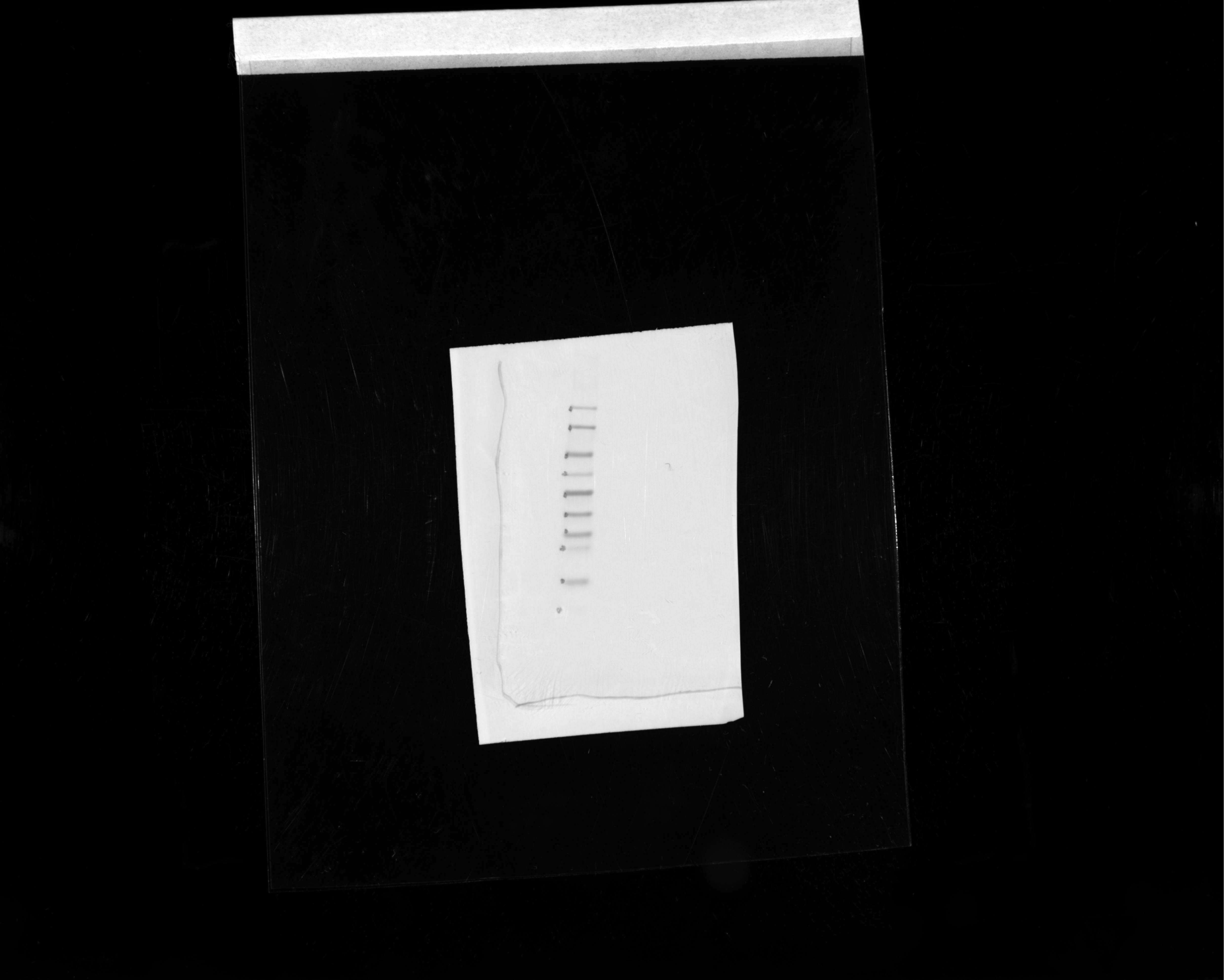

Supplement: Supplementary file 13 — Unprocessed western blots. [file 41565_2025_2011_MOESM13_ESM.zip › Source Data Extended Data Fig 3a_3e/Fig E3e_milk_CSN1S1_Colorimetric.jpg]

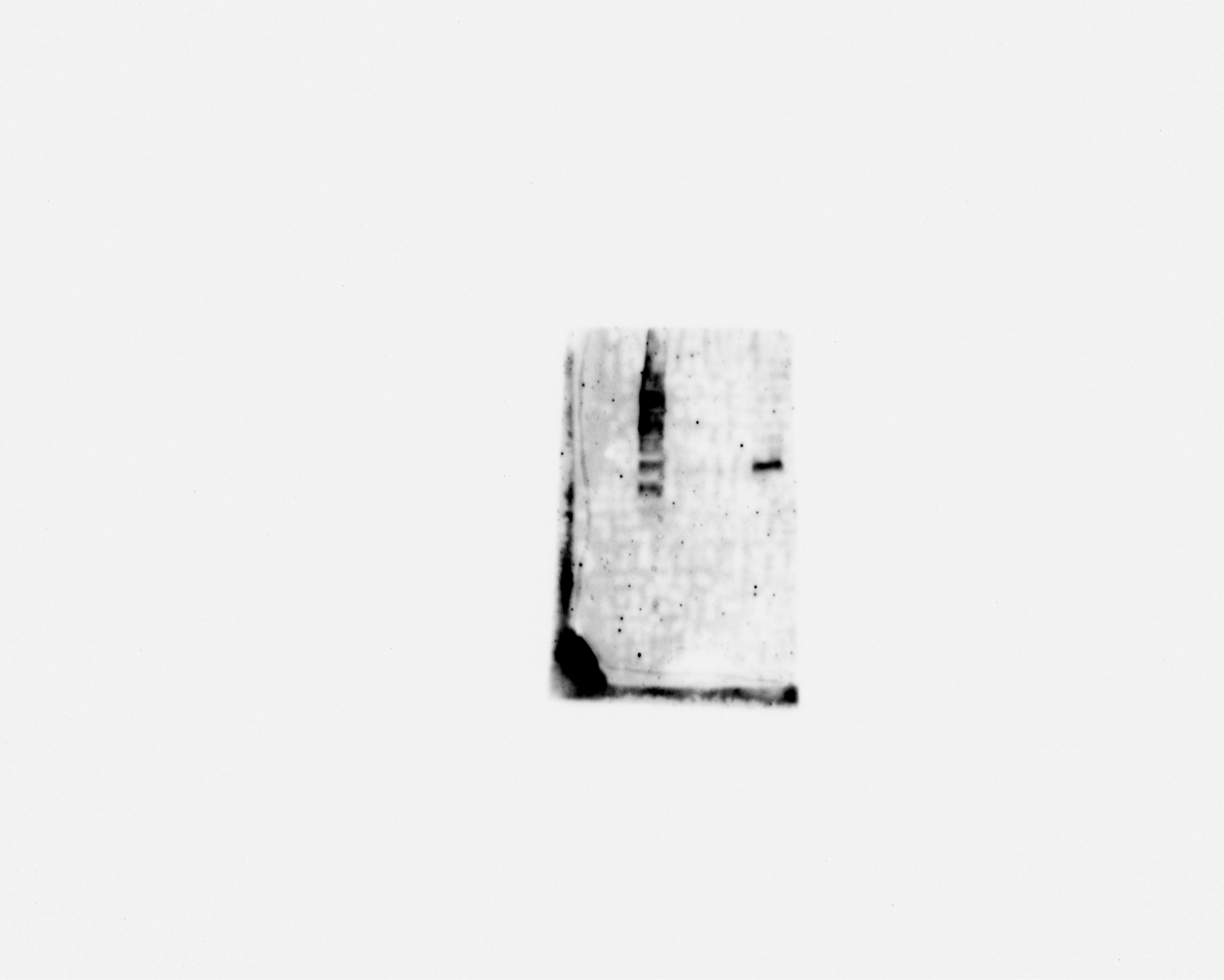

Supplement: Supplementary file 13 — Unprocessed western blots. [file 41565_2025_2011_MOESM13_ESM.zip › Source Data Extended Data Fig 3a_3e/Fig E3e_milk_GOLGA2_Chemiluminescence.jpg]

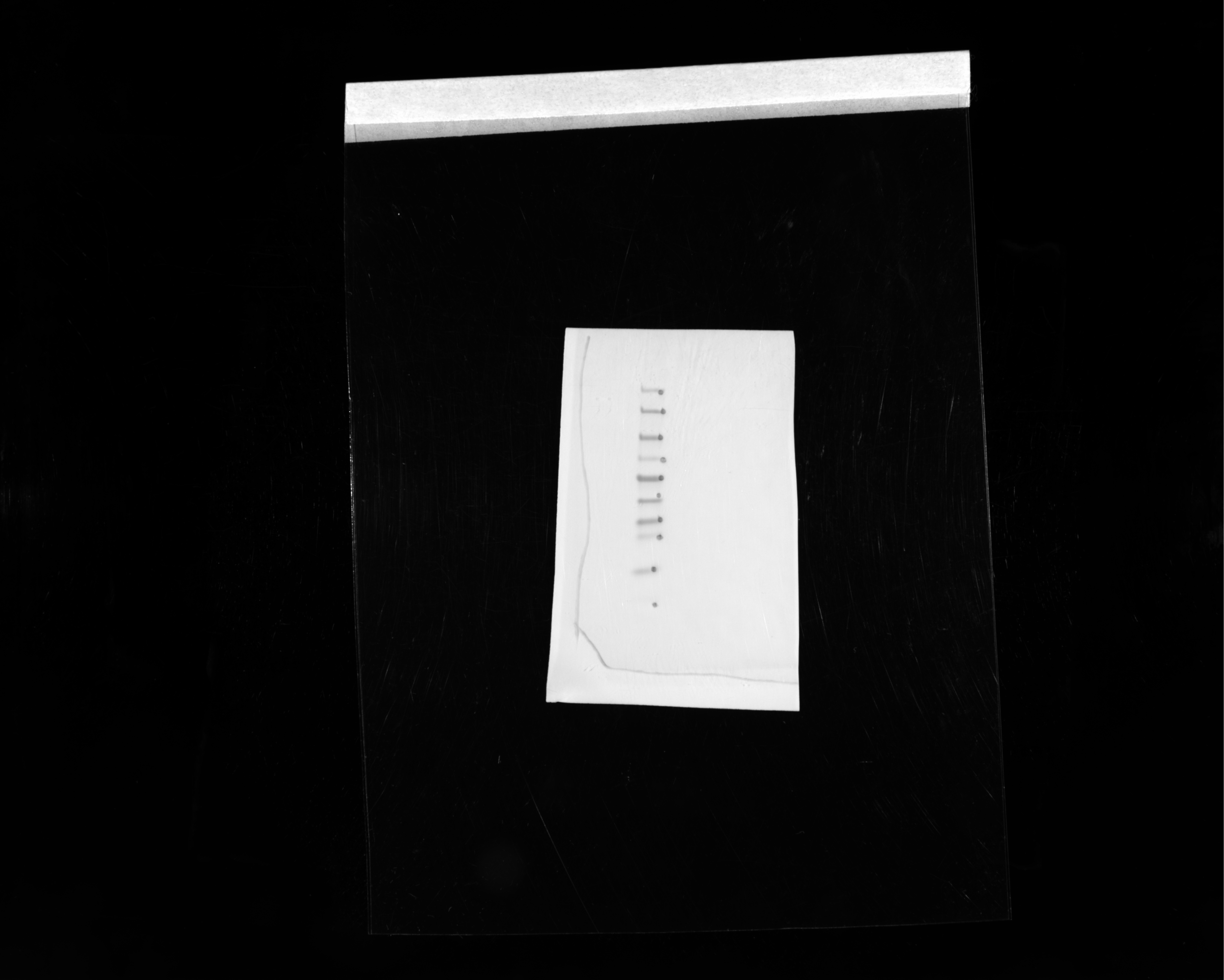

Supplement: Supplementary file 13 — Unprocessed western blots. [file 41565_2025_2011_MOESM13_ESM.zip › Source Data Extended Data Fig 3a_3e/Fig E3e_milk_GOLGA2_Colorimetric.jpg]

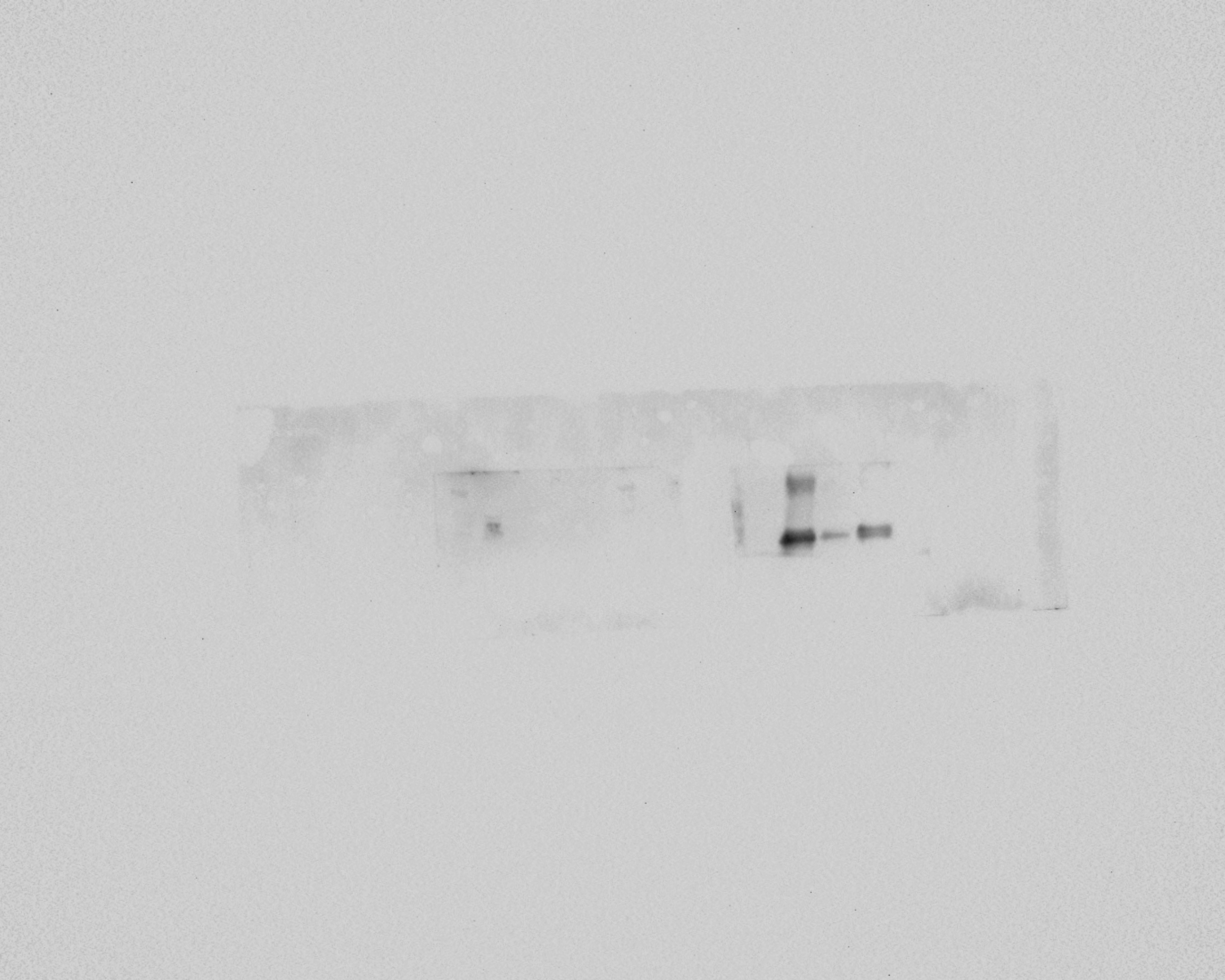

Supplement: Supplementary file 13 — Unprocessed western blots. [file 41565_2025_2011_MOESM13_ESM.zip › Source Data Extended Data Fig 3a_3e/Fig E3e_milk_TSG101_Chemiluminescence.jpg]

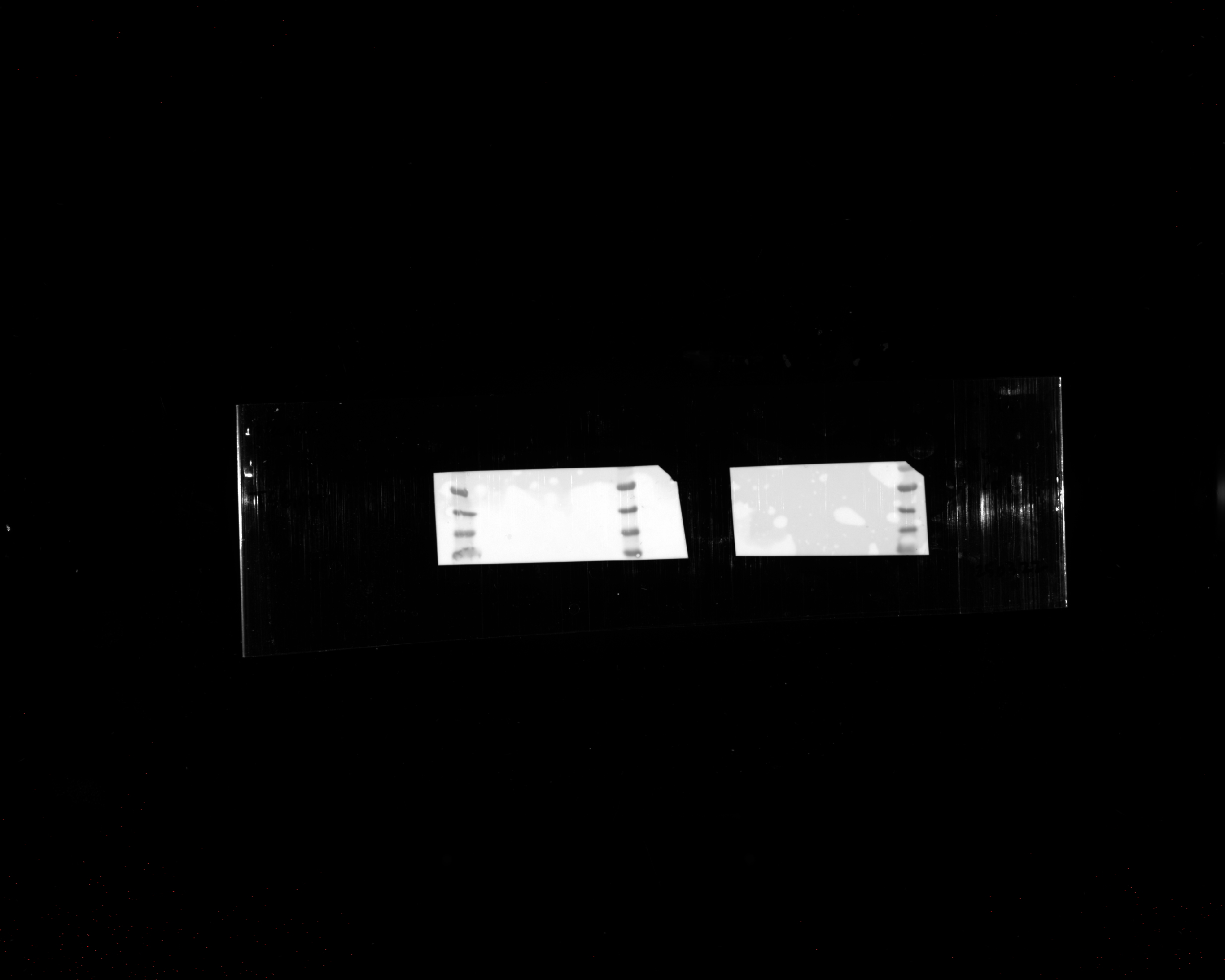

Supplement: Supplementary file 13 — Unprocessed western blots. [file 41565_2025_2011_MOESM13_ESM.zip › Source Data Extended Data Fig 3a_3e/Fig E3e_milk_TSG101_Colorimetric.jpg]

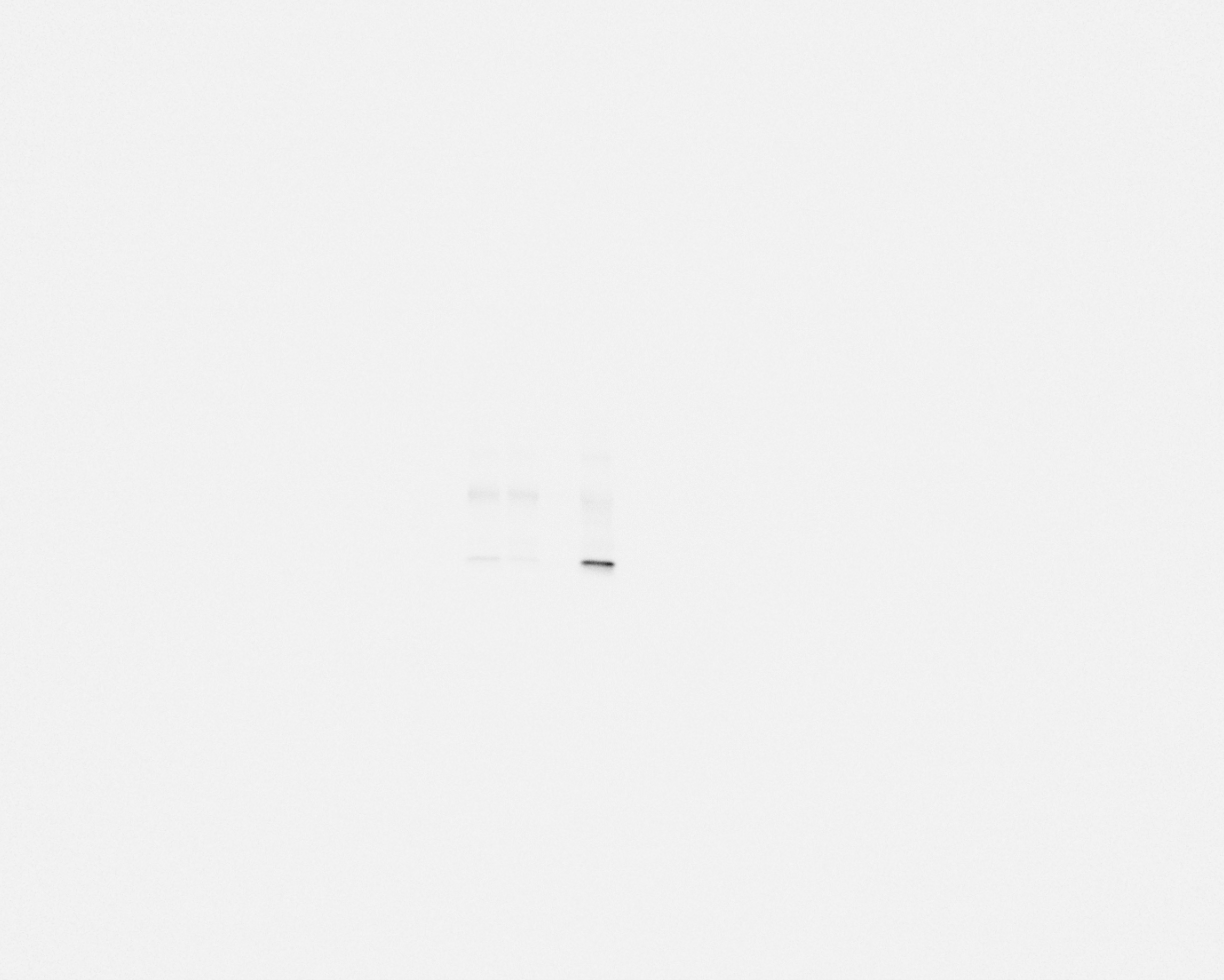

Supplement: Supplementary file 13 — Unprocessed western blots. [file 41565_2025_2011_MOESM13_ESM.zip › Source Data Extended Data Fig 3a_3e/Fig E3e_plasma_APOA1_Chemiluminescence.jpg]

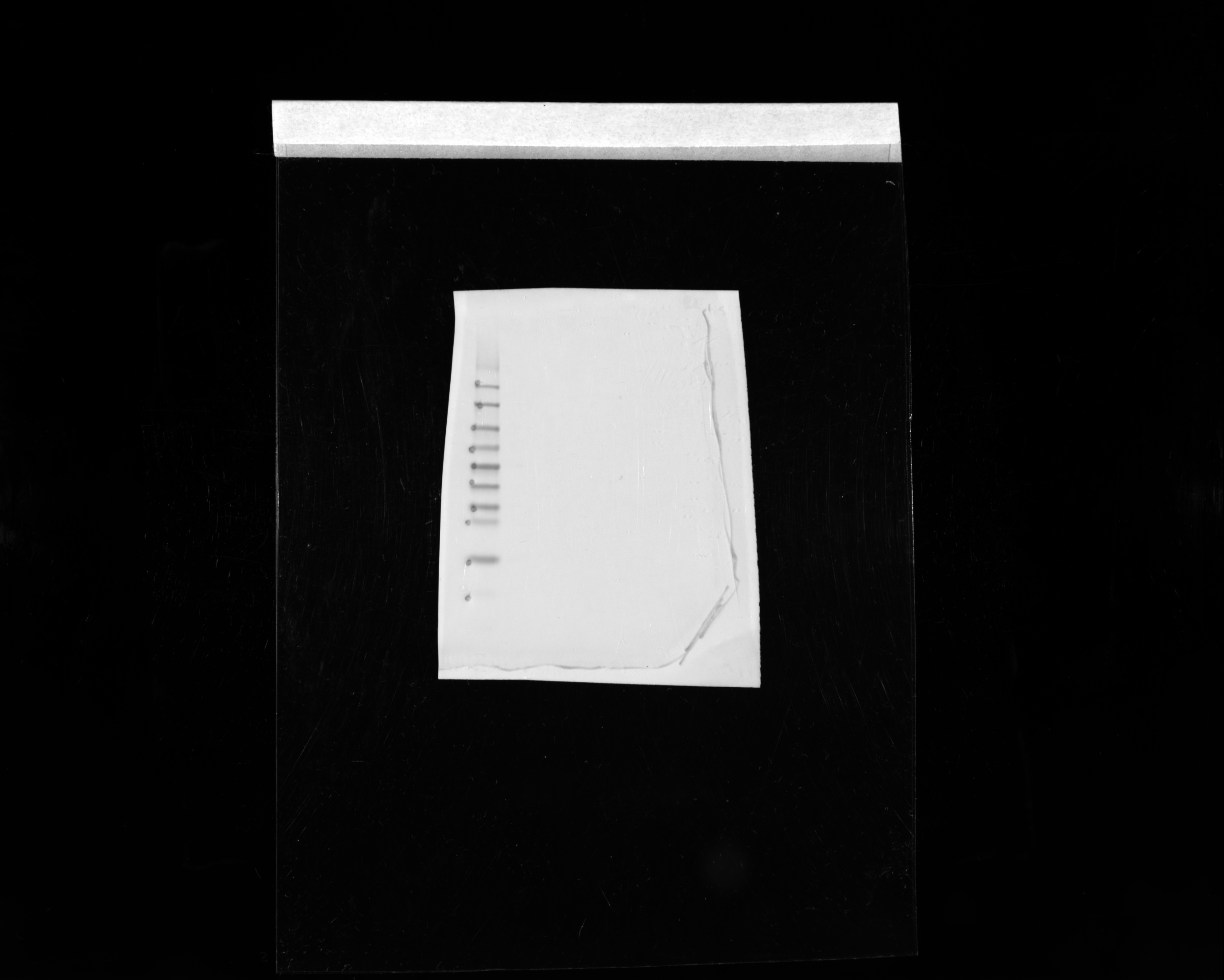

Supplement: Supplementary file 13 — Unprocessed western blots. [file 41565_2025_2011_MOESM13_ESM.zip › Source Data Extended Data Fig 3a_3e/Fig E3e_plasma_APOA1_Colorimetric.jpg]

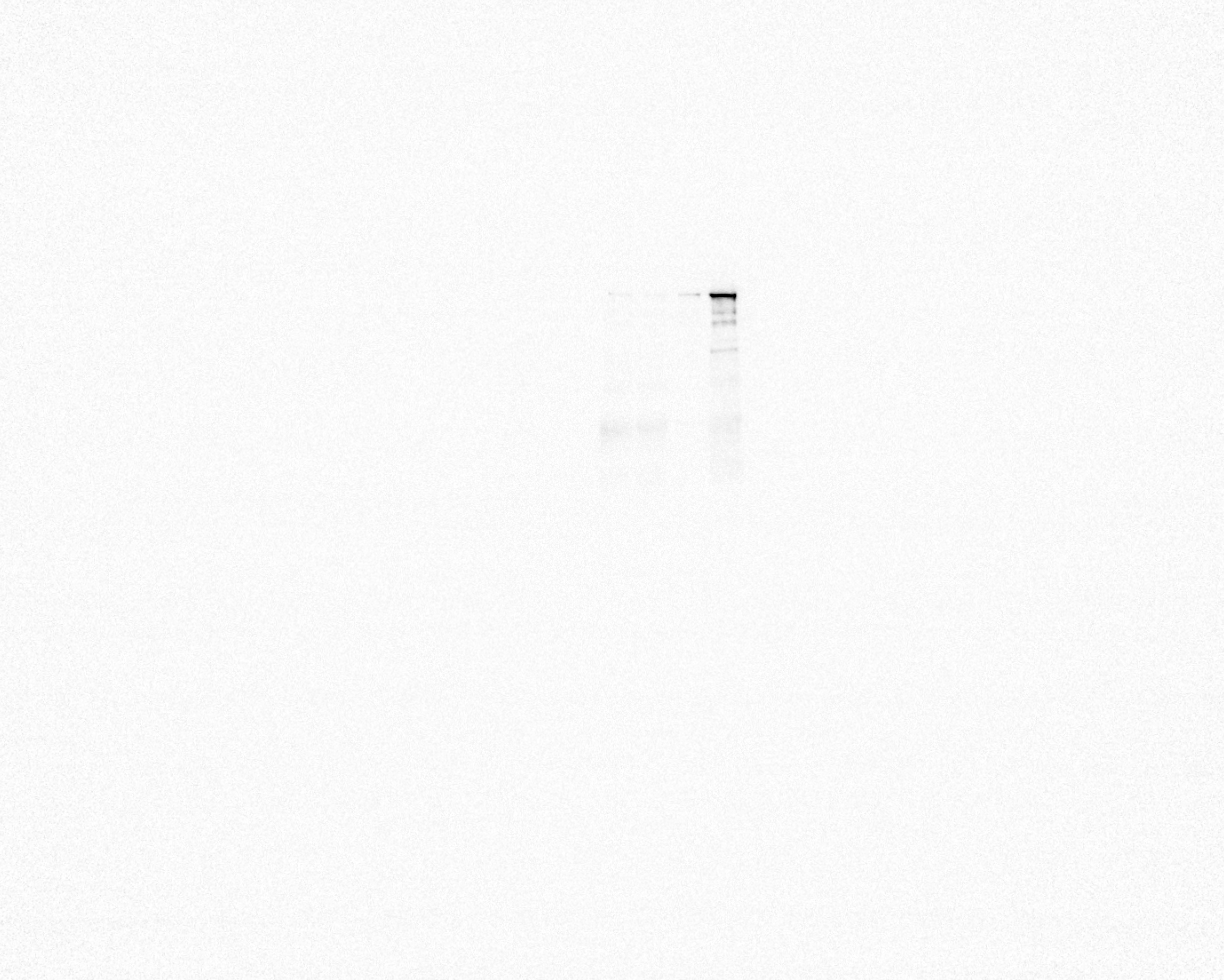

Supplement: Supplementary file 13 — Unprocessed western blots. [file 41565_2025_2011_MOESM13_ESM.zip › Source Data Extended Data Fig 3a_3e/Fig E3e_plasma_APOB_Chemiluminescence.jpg]

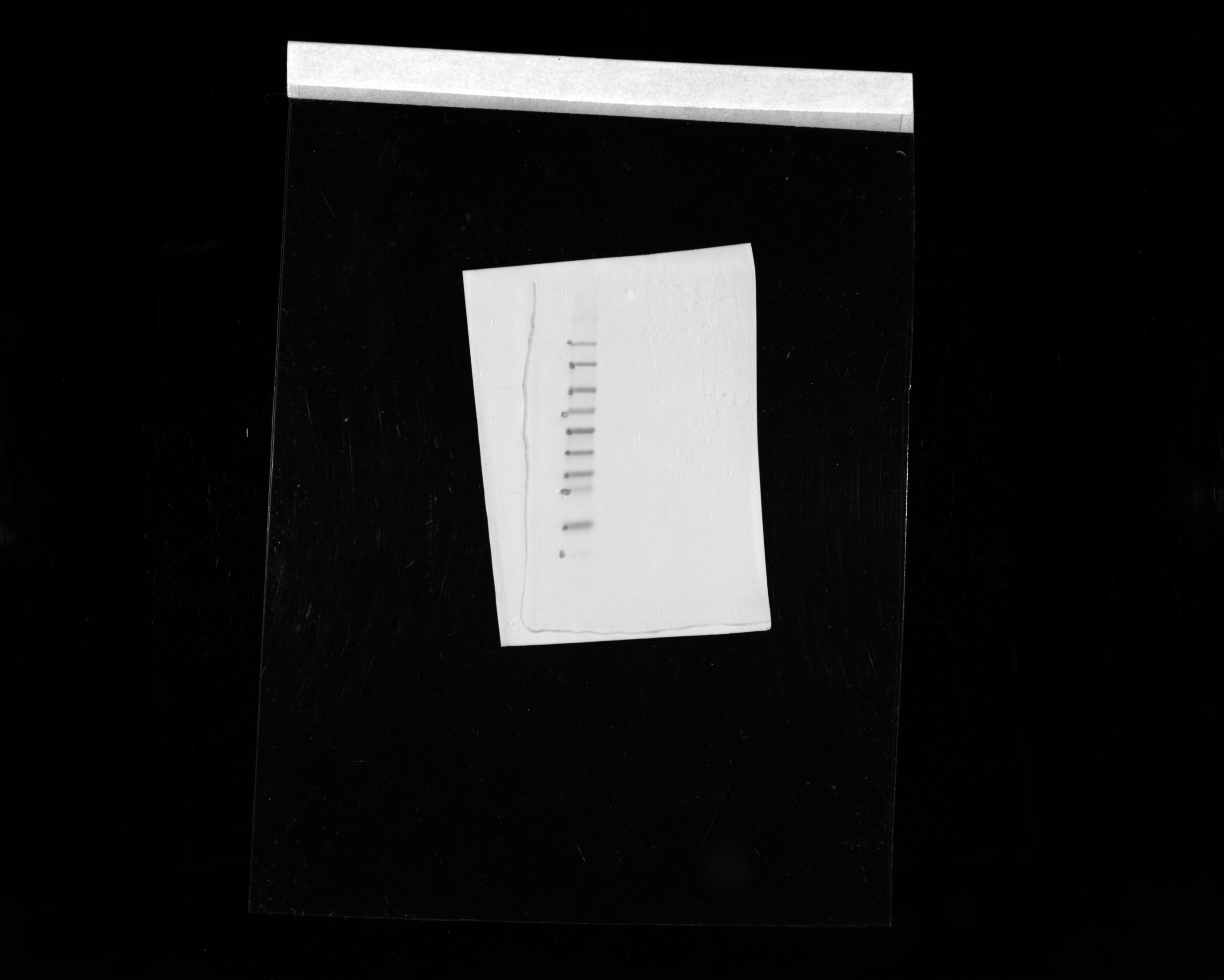

Supplement: Supplementary file 13 — Unprocessed western blots. [file 41565_2025_2011_MOESM13_ESM.zip › Source Data Extended Data Fig 3a_3e/Fig E3e_plasma_APOB_Colorimetric.jpg]

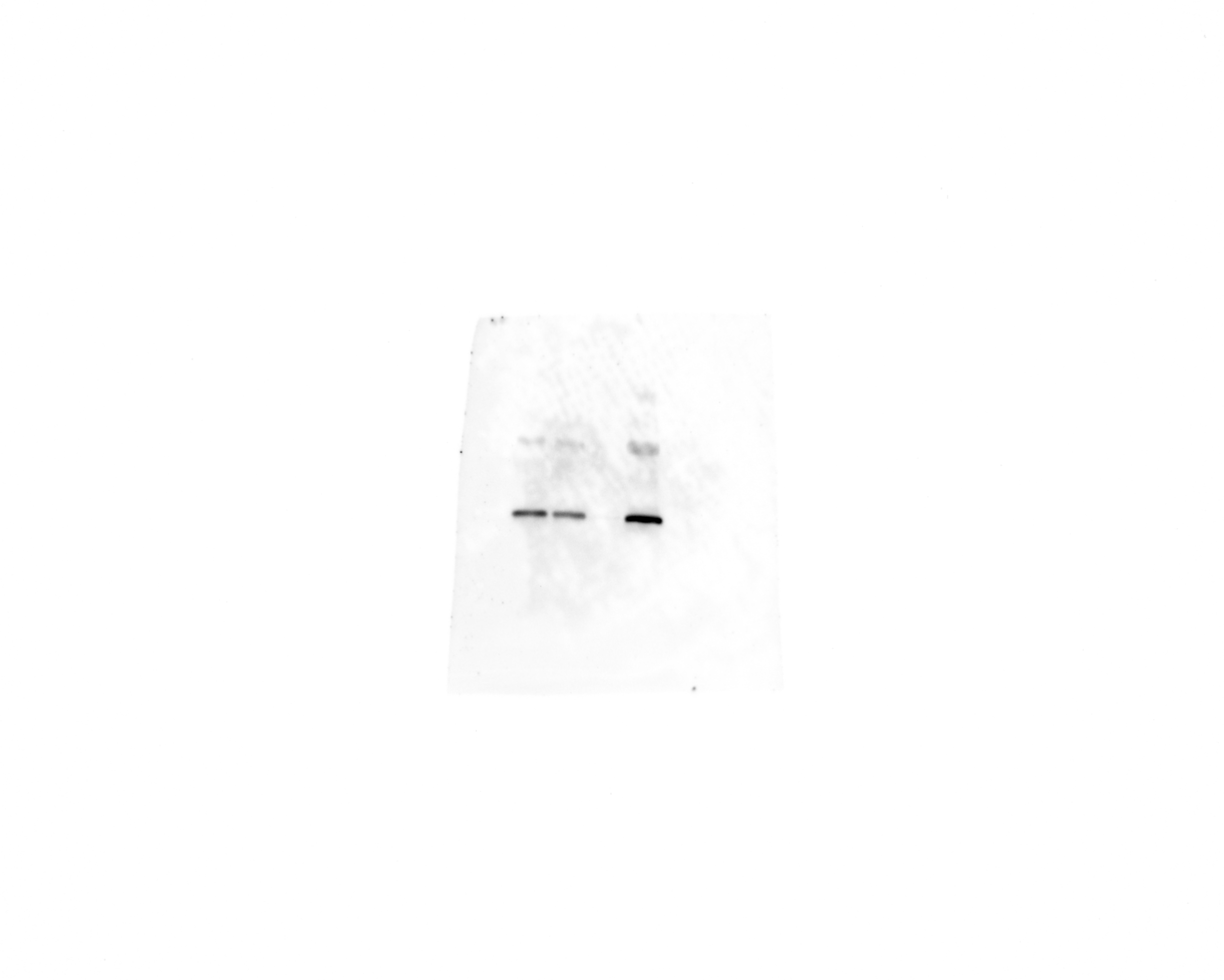

Supplement: Supplementary file 13 — Unprocessed western blots. [file 41565_2025_2011_MOESM13_ESM.zip › Source Data Extended Data Fig 3a_3e/Fig E3e_plasma_CD63_Chemiluminescence.jpg]

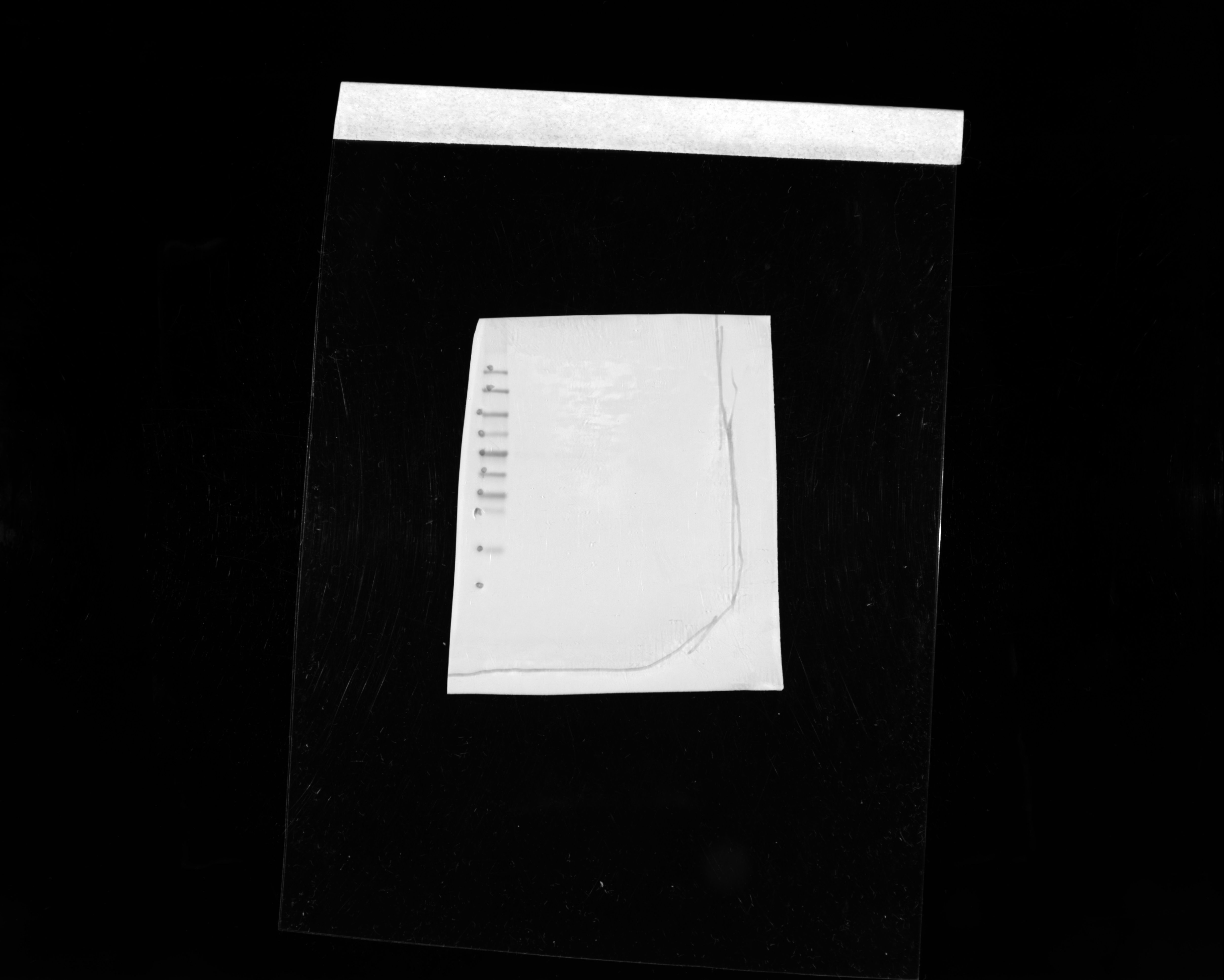

Supplement: Supplementary file 13 — Unprocessed western blots. [file 41565_2025_2011_MOESM13_ESM.zip › Source Data Extended Data Fig 3a_3e/Fig E3e_plasma_CD63_Colorimetric.jpg]

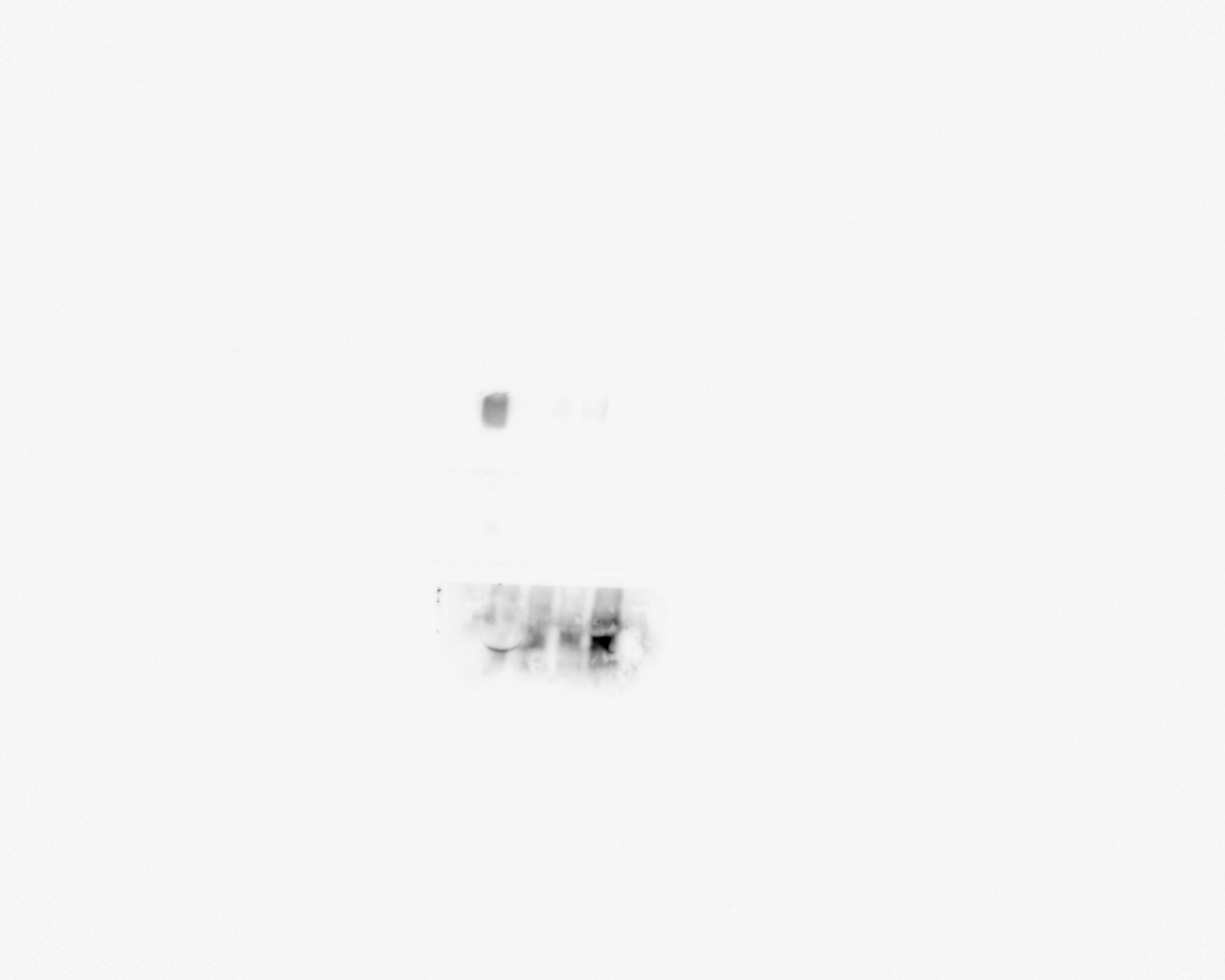

Supplement: Supplementary file 13 — Unprocessed western blots. [file 41565_2025_2011_MOESM13_ESM.zip › Source Data Extended Data Fig 3a_3e/Fig E3e_plasma_CNX_Chemiluminescence.jpg]

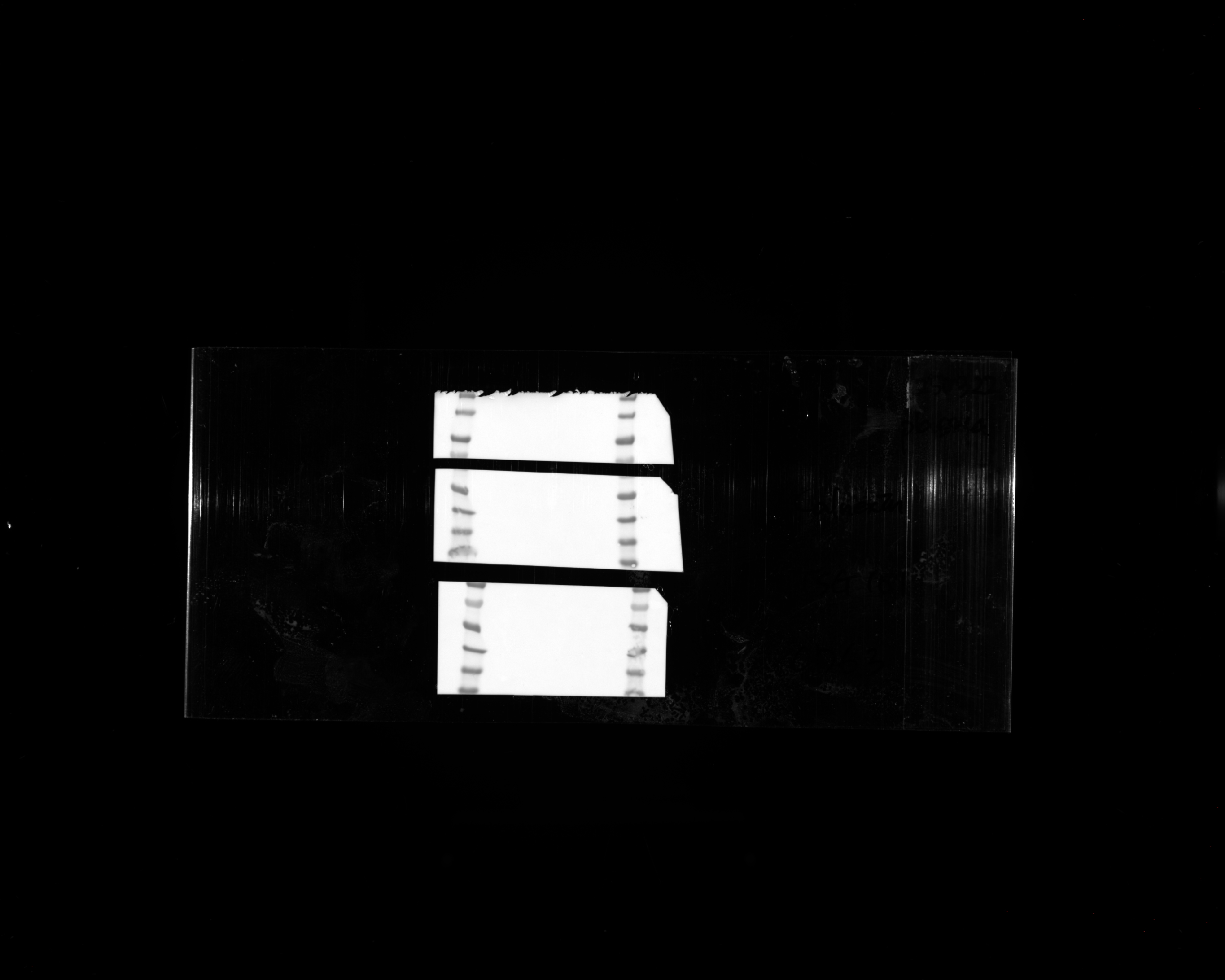

Supplement: Supplementary file 13 — Unprocessed western blots. [file 41565_2025_2011_MOESM13_ESM.zip › Source Data Extended Data Fig 3a_3e/Fig E3e_plasma_CNX_Colorimetric.jpg]

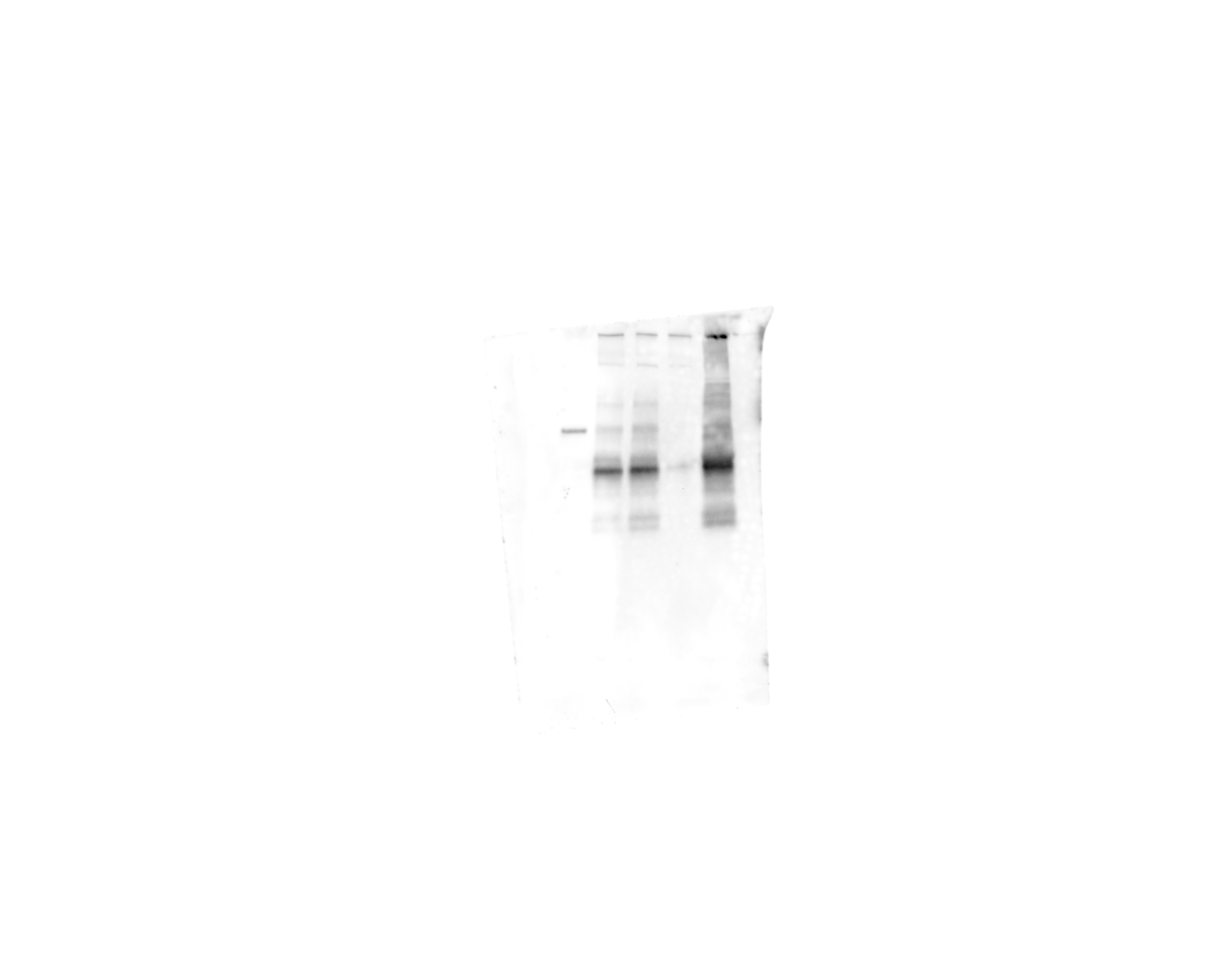

Supplement: Supplementary file 13 — Unprocessed western blots. [file 41565_2025_2011_MOESM13_ESM.zip › Source Data Extended Data Fig 3a_3e/Fig E3e_plasma_TSG101_Chemiluminescence.jpg]

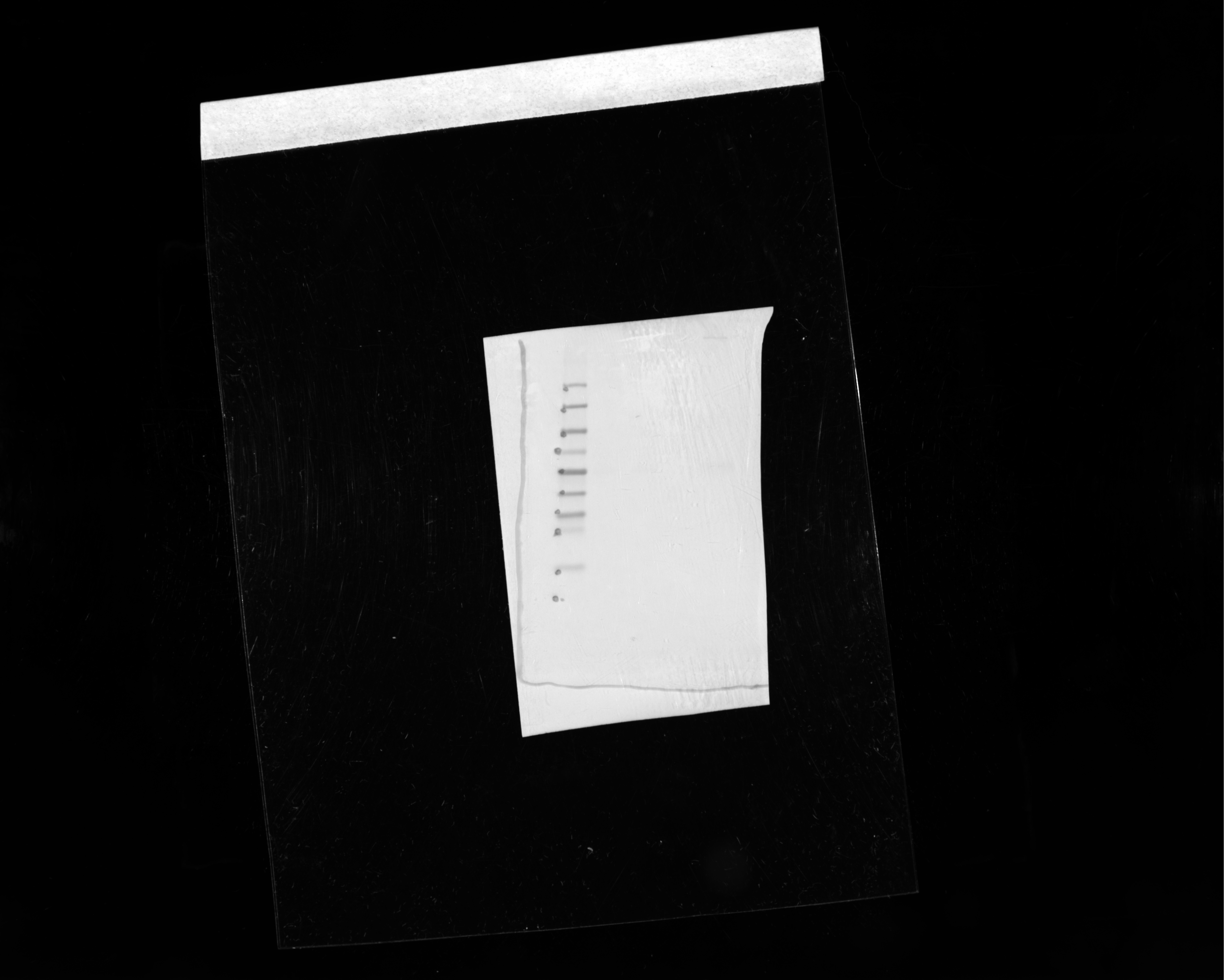

Supplement: Supplementary file 13 — Unprocessed western blots. [file 41565_2025_2011_MOESM13_ESM.zip › Source Data Extended Data Fig 3a_3e/Fig E3e_plasma_TSG101_Colorimetric.jpg]

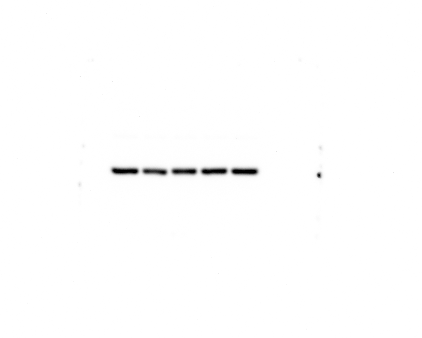

Supplement: Supplementary file 20 — Unprocessed western blots. [file 41565_2025_2011_MOESM20_ESM.zip › Source Data Extended Data Fig 10d_10f/Fig E10f_GAPDH_Chemiluminescence.tif]

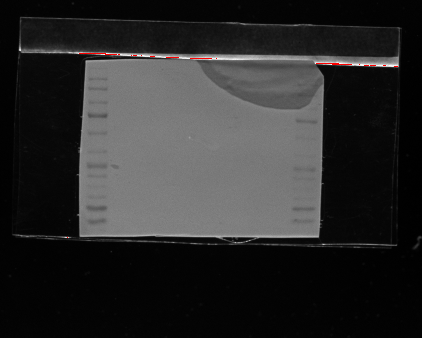

Supplement: Supplementary file 20 — Unprocessed western blots. [file 41565_2025_2011_MOESM20_ESM.zip › Source Data Extended Data Fig 10d_10f/Fig E10f_GAPDH_Colorimetric.tif]

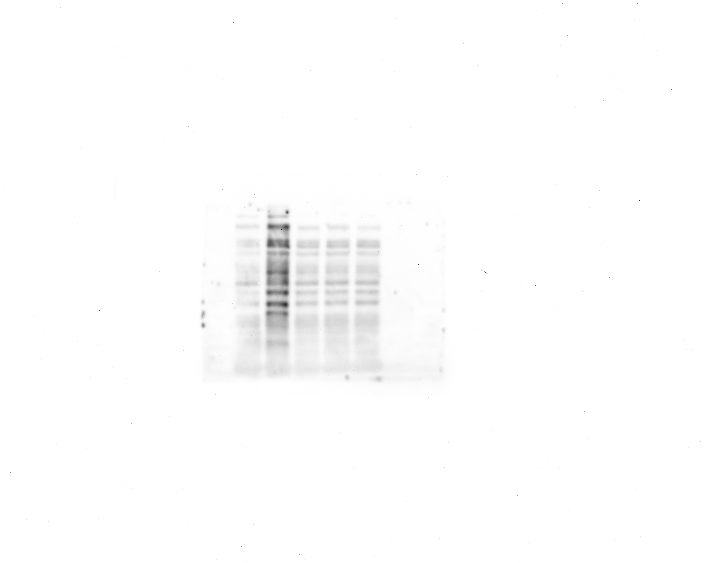

Supplement: Supplementary file 20 — Unprocessed western blots. [file 41565_2025_2011_MOESM20_ESM.zip › Source Data Extended Data Fig 10d_10f/Fig E10f_GSH_Chemiluminescence.tif]

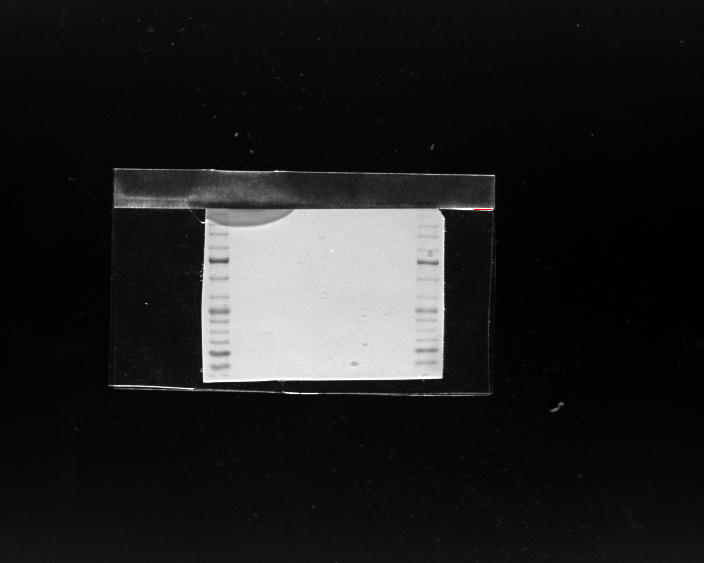

Supplement: Supplementary file 20 — Unprocessed western blots. [file 41565_2025_2011_MOESM20_ESM.zip › Source Data Extended Data Fig 10d_10f/Fig E10f_GSH_Colorimetric.tif]
